# The safety, feasibility, and oncological outcomes of laparoscopic completion total gastrectomy for remnant gastric cancer: a prospective study with 3-year follow-up (FUGES-004 study)

**DOI:** 10.1097/JS9.0000000000001388

**Published:** 2024-04-09

**Authors:** Qing Zhong, Dong Wu, Yi-ming Jiang, Qing-Liang He, Xue-Yi Dang, Dong-Bo Xu, Yu-Qin Sun, Guo-Qiang Su, Kai-Qing Guo, Li-Sheng Cai, Hao-xiang Zhang, Wen Ye, Guang-tan Lin, Ping Li, Jian-Wei Xie, Qi-Yue Chen, Chao-Hui Zheng, Jun Lu, Chang-Ming Huang, Jian-Xian Lin

**Affiliations:** a Department of Gastric Surgery, Fujian Medical University Union Hospital; b Key Laboratory of Ministry of Education of Gastrointestinal Cancer, Fujian Medical University; c Department of Gastrointestinal Surgery, The First Affiliated Hospital of Fujian Medical University; d Department of General Surgery, Shanxi Provincial Cancer Hospital, Shanxi Province; e Department of Gastrointestinal Surgery, Longyan First Hospital Affiliated to Fujian Medical University; f Department of General Surgery Unit 4, ZhangZhou Affiliated Hospital of Fujian Medical University, Zhangzhou; g Department of Gastrointestinal Surgery, The First Affiliated Hospital of Xiamen University, Xiamen, Fujian Province, People’s Republic of China

**Keywords:** disease-free survival, laparoscopic completion total gastrectomy, morbidity, open completion total gastrectomy, remnant gastric cancer

## Abstract

**Background:**

The efficacy of laparoscopic completion total gastrectomy (LCTG) for remnant gastric cancer (RGC) remains controversial.

**Methods:**

The primary outcome was postoperative morbidity within 30 days after surgery. Secondary outcomes included 3-year disease-free survival (DFS), 3-year overall survival (OS), and recurrence. Inverse probability treatment weighted (IPTW) was used to balance the baseline between LCTG and OCTG.

**Results:**

Final analysis included 46 patients with RGC who underwent LCTG at the FJMUUH between June 2016 and June 2020. The historical control group comprised of 160 patients who underwent open completion total gastrectomy (OCTG) in the six tertiary teaching hospitals from CRGC-01 study. After IPTW, no significant difference was observed between the LCTG and OCTG groups in terms of incidence (LCTG vs. OCTG: 28.0 vs. 35.0%, *P*=0.379) or severity of complications within 30 days after surgery. Compared with OCTG, LCTG resulted in better short-term outcomes and faster postoperative recovery. However, the textbook outcome rate was comparable between the two groups (45.9 vs. 32.8%, *P*=0.107). Additionally, the 3-year DFS and 3-year OS of LCTG were comparable to those of OCTG (DFS: log-rank *P*=0.173; OS: log-rank *P*=0.319). No significant differences in recurrence type, mean recurrence time, or 3-year cumulative hazard of recurrence were observed between the two groups (all *P*>0.05). Subgroup analyses and concurrent comparisons demonstrated similar trends.

**Conclusions:**

This prospective study suggested that LCTG was noninferior to OCTG in both short-term and long-term outcomes. In experienced centers, LCTG may be considered as a viable treatment option for RGC.

## Graphical abstract

**Figure:**
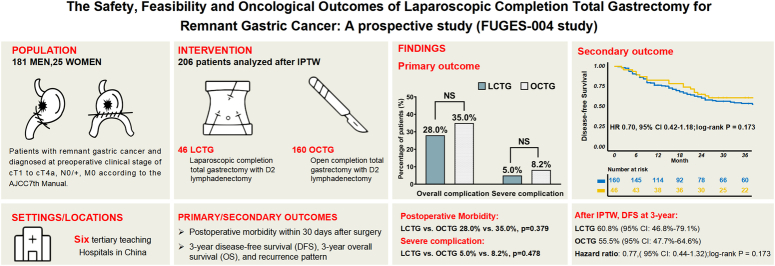

## Introduction

Highlights

In this prospective study, no significant difference was observed between the laparoscopic completion total gastrectomy (LCTG) and open completion total gastrectomy (OCTG) groups in terms of incidence or severity of complications within 30 days after surgery.

Moreover, the 3-year disease-free survival and 3-year overall survival of LCTG were comparable to those of OCTG.

In experienced centers, LCTG may be considered as a viable treatment option for remnant gastric cancer based on 3-year outcome of FUGES-004 study.

Recently, remnant gastric cancer (RGC) has been defined as cancer arising from the remnant stomach following initial gastrectomy, regardless of previous disease or surgical types. RGC is rare, accounting for only 1–8% of all gastric cancers^1,2^. Its early detection rate is increasing owing to increased endoscopic screening and intensive follow-up of primary gastric cancer. Compared with primary gastric cancer, RGC tends to be more aggressive and often diagnosed at a relatively advanced stage, resulting in a poorer prognosis. Currently, radical gastrectomy is one of the main treatment strategies for RGC^3^, making the choice of surgical approach a critical consideration for both patients and surgeons.

Compared with standard radical gastrectomy, it is more technically demanding for RGC due to prior gastrectomy-related adhesion and anatomical alterations, leading to inadvertent injury to surrounding tissues during surgery, which increases the incidence of intraoperative complications and mortality rate. Moreover, RGC is often diagnosed at an advanced stage, invading adjacent organs and necessitating multiviceral resections. Therefore, open completion total gastrectomy (OCTG) has become the primary surgical approach for treating RGC at the beginning. However, with the advent of minimally invasive surgery, Yamada *et al*.^4^ firstly reported the feasibility of laparoscopic completion total gastrectomy (LCTG) for RGC in 2005. Subsequently, other researchers have also explored its safety and feasibility. However, most previous studies were limited by their retrospective designs and small-sample sizes, thereby restricting the clinical applicability. Therefore, higher level of evidence is required to support the clinical use of LCTG. The FJMUUH Gastric Surgery Study Group (FUGES) launched the first prospective phase II clinical study (FUGES-004) to investigate the safety, feasibility, and long-term oncological outcomes of LCTG for resectable RGC. This study was registered at ClinicalTrials.gov as NCT02792881.

## Methods

### Study design

The FUGES-004 was a prospective, single-arm, open-label study conducted at the FJMUUH, a tertiary referral teaching hospital in China, from June 2016 to June 2020. The approved study protocol is available in Supplemental Digital Content 1, http://links.lww.com/JS9/C355. An independent safety and data monitoring committee monitored the progress of this trial. This study adhered to the Transparent Reporting of Evaluations with Non-Randomized Designs (TREND) reporting guidelines (Supplemental Digital Content 2, http://links.lww.com/JS9/C356).

### Study patients

The FUGES-004 study enrolled patients scheduled to undergo radical LCTG with D2 lymphadenectomy. The details on eligibility criteria are summarized in eTable 1 (Supplemental Digital Content 4, http://links.lww.com/JS9/C358). Finally, 46 patients were included in this study (eFigure 1, Supplemental Digital Content 5, http://links.lww.com/JS9/C359). Due to the low incidence and budget limitations, a randomized study was unfeasible; therefore, we selected patients who underwent radical OCTG with D2 lymphadenectomy between January 2003 and January 2017 in a retrospective multi-institution cohort study (Chinese remnant gastric cancer study, CRGC-01 study)^5^ as the control group (Details seen in eTable 2, Supplemental Digital Content 4, http://links.lww.com/JS9/C358), which was conducted to compare the surgical outcomes between LCTG and OCTG for RGC. To ensure inclusion and exclusion criteria were consistent with the FUGES-004 study, 14 patients were excluded from the CRGC-01 study, finally including 160 patients in the OCTG group (eFigure 1, Supplemental Digital Content 5, http://links.lww.com/JS9/C359). This study was approved by the institutional review board of FJMUUH (IRB number: 2016YF009-02) and followed the STROCSS criteria and guidelines^6^ (Supplemental Digital Content 6, http://links.lww.com/JS9/C360). The study was registered at Clinical Trials.gov (website is as following: https://classic.clinicaltrials.gov/ct2/show/study/NCT02792881).

### Surgical procedures, quality control, and follow-up

Both LCTG and OCTG adhered to the principles of total gastrectomy and lymphadenectomy from the Japanese gastric cancer treatment guidelines 2014 (ver.4)^7^. All patients were operated by either of the two surgeons (C.H.Z. or C.M.H.), both with over 100 LCTG experiences before this trial. Centers participating in the CRGC-01 study were all high-volume tertiary referral teaching hospitals in China with experienced gastric cancer surgeons.

Extracorporeal digestive reconstruction was performed. The surgical procedures of LCTG and OCTG were separated into five steps^8^ in the FJMUUH cohort (Details were observed in Video S1, Supplemental Digital Content 15, http://links.lww.com/JS9/C369). FUGES devised a checklist to assess the success of D2 lymphadenectomy (eTable 3, Supplemental Digital Content 4, http://links.lww.com/JS9/C358). In order to reduce the inherent limitations of multicenter study, the experienced surgeons participated in this study were selected from tertiary teaching hospitals based on strict standards (all had experience with more than 100 cases of laparoscopic gastrectomy per year). Additionally, respecting the principle of maximizing patient benefits^9,10^, surgeons must meet the strict qualifications set by the hospital safety monitoring committee, FUGES, and CRGC research group before conducting clinical studies about complicated surgery, especially radical gastrectomy of RGC. To avoid unnecessary injury to the patients and the difference of surgical methods due to surgeon-specific techniques and preferences, unedited videos (LCTG) or photographs (OCTG) of the participants were submitted to the Research Committee data center within 1 week after surgery and reviewed by another group of surgeons once a week for quality control. Finally, consistency analysis demonstrated no significant differences were seen in short-term and long-term outcomes between the FJMUUH and other external cohorts in the OCTG group (eTable 4, Supplemental Digital Content 4, http://links.lww.com/JS9/C358 and eFigure 2, Supplemental Digital Content 7, http://links.lww.com/JS9/C361, all *P*>0.05).

Resected specimens were evaluated pathologically following standardized criteria. Morbidity and mortality were assessed within 30 days after surgery. Patients in the LCTG and OCTG groups underwent uniform perioperative management and follow-up. Consistent with our previous study^11^, patients with pathological stage II or greater advanced disease were recommended to undergo a 6-month fluorouracil-based adjuvant chemotherapy.

The final follow-up date was 01st September 2023, with a minimum postoperative follow-up period of 36 months for each patient. Follow-up appointments were conducted every 3 months for the first 2 years and every 6 months for the subsequent 3 years (Routine follow-up procedures were observed in Supplemental Digital Content 3, http://links.lww.com/JS9/C357 named ‘Supplementary of Definition and Statistical method’).

### Outcomes and definitions

According to the 2nd and 3rd edition Japanese classification of gastric carcinoma^12,13^, RGC was defined as the carcinoma in the remnant stomach, which encompasses all carcinomas arising in the remnant stomach following a gastrectomy, irrespective of the histology of the primary lesion (benign or malignant) or its risk of recurrence, the extent of resection, or method of reconstruction. The LCTG and the historic OCTG groups adopted the same definition.

The primary endpoint was the overall postoperative morbidity rate, and the secondary endpoints were the 3-year overall survival (OS), 3-year disease-free survival (DFS), and recurrence pattern. Safety and efficacy outcomes included postoperative mortality rate (≤30 days), postoperative recovery, and textbook outcome (TO). OS was defined as the time from surgery to death from any cause or the last follow-up. DFS was defined as the time from surgery to recurrence, death from any cause, or the last follow-up.

Postoperative complications were graded according to the Clavien–Dindo classification. TO was defined as the same as previous literature^14^, including outcomes such as complete curative status; no intraoperative complications; no severe postoperative complications (Clavien–Dindo grade greater than III)^15,16^; at least 15 harvested lymph nodes; hospital stay less than 21 days; and no reintervention (surgical, radiological, or endoscopic), no readmission to the ICU, no 30-day postoperative mortality, and no hospital readmission within 30 days postdischarge. When above nine health outcomes were met, TO was achieved.

### Previous studies searching

English-language papers were searched in the databases including the PubMed, Embase, OVID, Web of Science, Cochrane Library, and ClinicalTrials.gov. The final search was performed in June 2023. Previous retrospective studies^3–5,17–39^ on ‘laparoscopic gastrectomy, open gastrectomy, and remnant gastric cancer’ are listed in eTable 5 (Supplemental Digital Content 4, http://links.lww.com/JS9/C358).

### Statistical analysis

The modified intention-to-treat (mITT) analysis set was used for conducting all analyses. Data management and site visit monitoring were performed by the data manager (M.L.). Continuous variables were displayed as mean±SD, and categorical variables as numbers. For each continuous and categorical variable, Student’s *t*-test, Pearson’s correlation test, or Fisher’s exact test were used to compare their distributions as appropriate.

### Inverse probability of treatment weighting

Inverse probability of treatment weighting (IPTW) was used to adjust for confounding between the intervention and control groups^40–42^. In this study, 28 covariates (eTable 6, Supplemental Digital Content 4, http://links.lww.com/JS9/C358), including patient characteristics and tumor findings, were identified. Investigators blinded to the outcome reviewed and checked the medical records, stored images, and laboratory data of all patients. A blinded bio-statistician (LY.Z.) performed the IPTW using generalized boosted method (GBM)^43^ to estimate the propensity score of each patient. Standardized mean difference (SMD) was used to evaluate the balance of covariates. Factors with SMD>0.1 were defined as imbalance (Details of IPTW were observed in Supplemental Digital Content 3, named ‘Supplementary of Definition and Statistical method’).

The adjusted Kaplan–Meier curves, log-rank test, and multivariable Cox regression analyses were used to compare OS, DFS, and recurrence between the two groups. Additionally, subgroup analyses and concurrent comparisons were performed to eliminate grouping and time bias between LCTG and OCTG, respectively.

Statistical analysis was analyzed through *SPSS statistical* software, version 25.0 (SPSS Inc), and the *R software* version 4.2.0 (R Foundation for Statistical Computing) from March to September 2023.

## Results

### Patient characteristics

After excluding four patients who underwent exploratory surgery, 46 patients who underwent LCTG were included in the mITT analysis, including 41 (89.1%) male and 5 (10.9%) female patients. Most patients (54.3%) were aged >65 years. Among them, open conversion occurred in two patients (4.3%) because of technical difficulty (one patient) and adhesion (one patient). A total of 160 patients who underwent OCTG were included in the historical control group, including 140 (87.5%) male and 20 (12.5%) female patients, with 75 patients (46.9%) aged >65 years old. After IPTW, baseline characteristics were comparable between the LCTG and OCTG groups (SMD<0.1, all *P*>0.05; Table 1).

**Table 1:** Clinical characteristics of LCTG and OCTG before and after IPTW.

	Before IPTW				After IPTW			
Clinical characteristics	OCTG (*N*=160)	LCTG (*N*=46)	SMD	*P*	OCTG (*N*=158.9)	LCTG (*N*=43.4)	SMD	*P*
Age *n* (%)			0.150	0.371			0.060	0.723
<65 years	85 (53.1)	21 (45.7)			83.6 (52.6)	24.1 (55.6)		
≥65 years	75 (46.9)	25 (54.3)			75.3 (47.4)	19.3 (44.4)		
Sex *n* (%)			0.051	0.765			0.090	0.586
Male	140 (87.5)	41 (89.1)			139.8 (88.0)	36.8 (84.9)		
Female	20 (12.5)	5 (10.9)			19.1 (12.0)	6.6 (15.1)		
Comorbidity *n* (%)			0.078	0.640			0.093	0.581
No	100 (62.5)	27 (58.7)			99.2 (62.4)	25.1 (57.8)		
Yes	60 (37.5)	19 (41.3)			59.7 (37.6)	18.3 (42.2)		
ASA scores *n* (%)			0.011	0.946			0.068	0.682
I	135 (84.4)	39 (84.8)			133.5 (84.0)	35.3 (81.4)		
II–III	25 (15.6)	7 (15.2)			25.4 (16.0)	8.1 (18.6)		
Previous surgical type *n* (%)			/	/				
Distal gastrectomy + Billroth-I	46 (28.8)	9 (19.6)						
Distal gastrectomy + Billroth-II	102 (63.8)	37 (80.4)						
Distal gastrectomy + Roux-en-y	2 (1.3)	/						
Proximal gastrectomy + Gastroesophageal anastomosis	7 (4.4)	/						
Partial gastrectomy + Billroth-I	2 (1.3)	/						
Partial gastrectomy + Billroth-II	1 (0.6)	/						
Oncological characteristics								
Histology *n* (%)			0.329	0.090			0.035	0.979
Differentiated	63 (39.4)	18 (39.1)			62.3 (39.2)	17.7 (40.8)		
Undifferentiated	88 (55.0)	21 (45.7)			84.7 (53.3)	22.4 (51.6)		
Gx	9 (5.6)	7 (15.2)			11.8 (7.4)	3.3 (7.6)		
Pathologic T Stage *n* (%)			0.350	0.025			0.059	0.725
T1	20 (12.5)	12 (26.1)			24.8 (15.6)	7.7 (17.8)		
≥T2	140 (87.5)	34 (73.9)			134.1 (84.4)	35.7 (82.2)		
Pathologic N Stage *n* (%)			0.062	0.711			0.062	0.715
N0	89 (55.6)	27 (58.7)			89.3 (56.2)	23 (53.1)		
N+	71 (44.4)	19 (41.3)			69.6 (43.8)	20.4 (46.9)		
Tumor location *n* (%)			0.264	0.118			0.099	0.560
Anastomoic site	73 (45.6)	27 (58.7)			77.9 (49.0)	23.4 (53.9)		
Nonanastomoic site	87 (54.4)	19 (41.3)			81 (51.0)	20 (46.1)		
Tumor size, mm	45.5±20.3	37.3±21.7	0.388	0.012	43.4±20.3	42.3±23.4	0.047	0.772
LVI n (%)			0.022	0.896			0.087	0.606
No	106 (66.3)	30 (65.2)			103.3 (65.0)	26.4 (60.9)		
Yes	54 (33.8)	16 (34.8)			55.6 (35.0)	17 (39.2)		
Neoadjuvant chemotherapy *n* (%)			0.326	0.035			0.050	0.767
No	144 (90.0)	36 (78.3)			137.8 (86.7)	36.8 (84.9)		
Yes	16 (10.0)	10 (21.7)			21.1 (13.3)	6.6 (15.1)		
Adjuvant chemotherapy *n* (%)			0.138	0.414			0.013	0.937
No	77 (48.1)	19 (41.3)			73.1 (46.0)	19.7 (45.4)		
Yes	83 (51.9)	27 (58.7)			85.8 (54.0)	23.7 (54.6)		
Open conversion *n* (%)	/	2 (4.3)	/	/				
Technique difficulty	/	1 (2.2)						
Severe adhesion	/	1 (2.2)						

### Postoperative morbidity (Primary outcomes)

No significant difference in the incidence (LCTG vs. OCTG: 28.0 vs. 35.0%, *P*=0.379; Table 2) or severity of complications within 30 days after surgery was observed between the two groups. Additionally, each subtype of postoperative morbidity was comparable between the two groups (all *P*>0.05).

**Table 2:** Postoperative morbidity of patients with RGC after IPTW.

	OCTG (*N*=158.9)	LCTG (*N*=43.4)	*P*
Overall morbidity, *n* (%)	55.6 (35.0)	12.2 (28.0)	0.379
Surgical morbidity			
Anastomotic leak, *n* (%)	10.0 (6.3)	2.5 (5.7)	0.889
Wound infection, *n* (%)	17.3 (10.9)	1.4 (3.2)	0.121
Ileus and gastroparesis, *n* (%)	6.7 (4.2)	2.6 (5.9)	0.623
Abdominal or anastomotic bleeding, *n* (%)	5.1 (3.2)	3.7 (8.6)	0.118
Abdominal abcess, *n* (%)	7.5 (4.7)	1.0 (2.4)	0.505
Medical morbidity			
Urinary complications, *n* (%)	1.9 (1.2)	0.7 (1.5)	0.863
Respiratory complication, *n* (%)	35.8 (22.5)	6.0 (13.9)	0.215
Cerebrocardiovascular complication, *n* (%)	1.7 (1.1)	0 (0)	0.489
Clavien–Dindo Grade≥III, *n* (%)	13.0 (8.2)	2.2 (5.0)	0.478

### Secondary outcomes

#### Surgical outcomes and recovery from surgery

Compared with OCTG, LCTG resulted in less estimated blood loss (59.7 vs. 220.4 ml, *P*<0.001; eTable 7, Supplemental Digital Content 4, http://links.lww.com/JS9/C358), shorter operation time (163.9 vs. 225.7 min, *P*<0.001), and more harvested lymph nodes (19.2 vs. 15.6 nodes, *P*=0.030). Additionally, the time to intra-abdominal drain removal and postoperative hospital stay were significantly shorter in the LCTG group (both *P*<0.05; eTable 7, Supplemental Digital Content 4, http://links.lww.com/JS9/C358); however, no significant differences in the time to flatus and time to initiation of solid food intake were observed (both *P*>0.05).

#### Textbook outcome

The LCTG group demonstrated a comparable incidence of curative status, intraoperative complications, reintervention, unplanned ICU treatment, hospital readmission, severe complications, and postoperative mortality (eFigure 3A, Supplemental Digital Content 8, http://links.lww.com/JS9/C362). Although the TO rate was not significantly different (*P*=0.107), a higher proportion of patients in the LCTG group achieved TO than that of patients in the OCTG group (45.9 vs. 32.8%).

#### Disease-free survival

**Figure 1:**
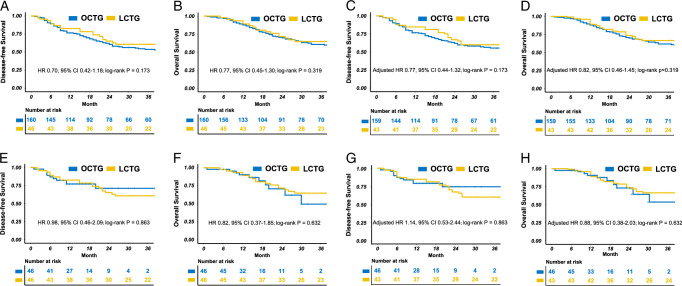
Kaplan–Meier curves of overall survival and disease-free survival for patients with remnant gastric cancer before (A, B) and after (C, D) IPTW. Concurrent comparison of survival curves of overall survival and disease-free survival for patients with remnant gastric cancer before (E, F) and after IPTW (G, H).

The median follow-up time was 61.0 months (range: 47–72 months). Before IPTW, the 3-year DFS were 63.2% (95% CI: 51.0–78.3%) and 51.7% (95% CI: 44.0–60.6%) in the LCTG and OCTG groups, respectively (log-rank *P*=0.173; Fig. 1A). After IPTW, LCTG still had 3-year DFS rates comparable to that of OCTG [60.8% (95% CI: 46.8–79.1%) vs. 55.5% (95% CI: 47.7–64.6%), log-rank *P*=0.173; Fig. 1C). Univariable analysis revealed associations between DFS and histology, pathological T and N stages, tumor size, LVI, and adjuvant chemotherapy. Multivariable analysis identified histology and pathological N stage as independent risk factors for DFS, with the exception of LCTG (HR 0.70; 95% CI: 0.42–1.18; *P*=0.179; Table 3).

**Table 3:** Uni-variate and multivariate analysis of the disease-free survival for RGC patients.

	Univariate analysis		Multivariate analysis	
	HR (95% CI)	*P*	HR (95% CI)	*P*
Age				
<65 years	1			
≥65 years	1.04 (0.71–1.52)	0.848		
Sex				
Male	1			
Female	1.62 (0.96–2.72)	0.068		
Comorbidity				
No	1			
Yes	1.02 (0.70–1.50)	0.916		
ASA scores				
I	1			
II–III	0.80 (0.47–1.36)	0.400		
Surgical approaches				
OCTG	1			
LCTG	0.70 (0.42–1.18)	0.179		
Histology				
Differentiated	1		1	
Undifferentiated	2.00 (1.31–3.05)	**0.001**	1.61 (1.03–2.52)	**0.036**
Pathologic T stage				
T1	1		1	
≥T2	3.90 (1.71–8.89)	**0.001**	2.42 (0.99–5.94)	0.053
Pathologic N stage				
N0	1		1	
N+	0.44 (0.29–0.66)	**<0.001**	0.63 (0.40–0.99)	**0.044**
Tumor location				
Anastomoic site	1			
Nonanastomoic site	1.02 (0.70–1.50)		0.902	
Tumor size	1.02 (1.01–1.03)	**<0.001**	1.01 (1.00–1.02)	0.207
LVI				
No	1			
Yes	1.62 (1.11–2.38)	**0.013**	1.03 (0.68–1.57)	0.882
Neoadjuvant chemotherapy				
No	1			
Yes	1.21 (0.70–2.09)	0.492		
Adjuvant chemotherapy *n* (%)				
No	1			
Yes	1.50 (1.02–2.22)	**0.041**	1.06 (0.70–1.60)	0.792

The datasets used and/or analyzed during the current study are available from the corresponding author on reasonable request.

#### Overall survival

Before and after IPTW, no difference in 3-year OS (log-rank *P*=0.319; Fig. 1B, D) was observed between the groups. Univariable analysis revealed associations between OS and histology, pathological T and N stages, tumor size, and LVI. Multivariable analysis identified histology and pathological N stage as independent risk factors for OS, with the exception of LCTG (HR 0.77; 95% CI: 0.45–1.30; *P*=0.323; eTable 8, Supplemental Digital Content 4, http://links.lww.com/JS9/C358).

#### Recurrence

Before IPTW, the 3-year cumulative hazard of recurrence was comparable between the two groups [33.3% (95% CI: 18.5–46.6%) vs. 37.8% (95% CI: 28.8–45.8%), log-rank *P*=0.695; eFigure 4A, Supplemental Digital Content 9, http://links.lww.com/JS9/C363]. No significant differences in local recurrence, peritoneal recurrence, or distant metastasis were observed between the two groups (eFigure 4B–D, Supplemental Digital Content 9, http://links.lww.com/JS9/C363, log-rank *P*>0.05). Further analysis revealed that the mean recurrence times in both the groups were comparable in terms of overall recurrence or different types of recurrence (*P*>0.05; eTable 9, Supplemental Digital Content 4, http://links.lww.com/JS9/C358). After IPTW, no significant differences were observed in recurrence types, mean recurrence time, or 3-year cumulative hazard of recurrence between the two groups (all *P*>0.05; eFigure 4I–L, Supplemental Digital Content 9, http://links.lww.com/JS9/C363 and eTable 9, Supplemental Digital Content 4, http://links.lww.com/JS9/C358). Additionally, LCTG was not identified as an independent factor associated with recurrence-free survival (RFS) (HR 0.89, 95% CI: 0.50–1.59, *P*=0.697; eTable 10, Supplemental Digital Content 4, http://links.lww.com/JS9/C358). Recurrences were observed in 70 patients (70/206, 34.0%). eFigure 5A (Supplemental Digital Content 10, http://links.lww.com/JS9/C364) illustrates the distribution of the recurrence sites in all patients. Most patients (47/70, 67.1%) had an initial recurrence involving only a single site, 21 (21/70, 30.0%) had an initial recurrence involving two sites, and two patients (2/70, 2.9%) had an initial recurrence involving all three sites. Recurrences were observed in 15 patients in the LCTG group. Distant metastasis was the most common site of recurrence (7/15, 46.7%), followed by multifocal (3/15, 20.0%), peritoneal (3/15, 20.0%), and locoregional (2/15, 13.3%) recurrence (eFigure 5B, Supplemental Digital Content 10, http://links.lww.com/JS9/C364). Similarly, in the OCTG group, distant metastasis was the most common (21/55, 38.2%), followed by multifocal (20/55, 36.4%), peritoneal (10/55, 18.2%), and locoregional (4/55, 7.3%) recurrence (eFigure 5C, Supplemental Digital Content 10, http://links.lww.com/JS9/C364).

#### Concurrent comparison

In concurrent comparison, the incidence of overall or specific types of complications was similar between the two groups (*P*>0.05; eTable 11, Supplemental Digital Content 4, http://links.lww.com/JS9/C358). Although intraoperative blood loss and postoperative recovery (time to removal of intra-abdominal drains, time to initiation of solid food intake, and postoperative hospital stay) in the LCTG group were significantly better than those in the OCTG group (all *P*<0.05; eTable 12, Supplemental Digital Content 4, http://links.lww.com/JS9/C358), no significant differences in TO rates were observed between the two groups (*P*>0.05; eFigure 3B, Supplemental Digital Content 8, http://links.lww.com/JS9/C362). The 3-year DFS in the LCTG group was 61.6% (95% CI: 49.2–77.2%) and 60.8% (95% CI: 46.7–79.2%) before and after IPTW, respectively, which was similar to those of the OCTG group (log-rank *P*=0.863; Fig. 1E, G). Similarly, the 3-year OS of the LCTG group was comparable to that of the OCTG group (HR 0.88, 95% CI: 0.38–2.03, log-rank *P*=0.632; Fig. 1F, H). Additionally, the cumulative hazard was comparable between the two groups for overall recurrence or any specific recurrence type. Meanwhile, the mean recurrence times were comparable in both the groups (*P*>0.05) (eFigure 4 E–H, M–P, Supplemental Digital Content 9, http://links.lww.com/JS9/C363 and eTable 13, Supplemental Digital Content 4, http://links.lww.com/JS9/C358).

#### Subgroup analysis

Compared with mITT population, subgroup analysis showed similar trends in the operation time, intraoperative bleeding loss, and postoperative rehabilitation process (time to first flatus, initiation of solid food intake, removal of intra-abdominal drains, and postoperative hospital duration) between the LCTG and OCTG groups (eFigure 6, Supplemental Digital Content 11, http://links.lww.com/JS9/C365).

**Figure 2:**
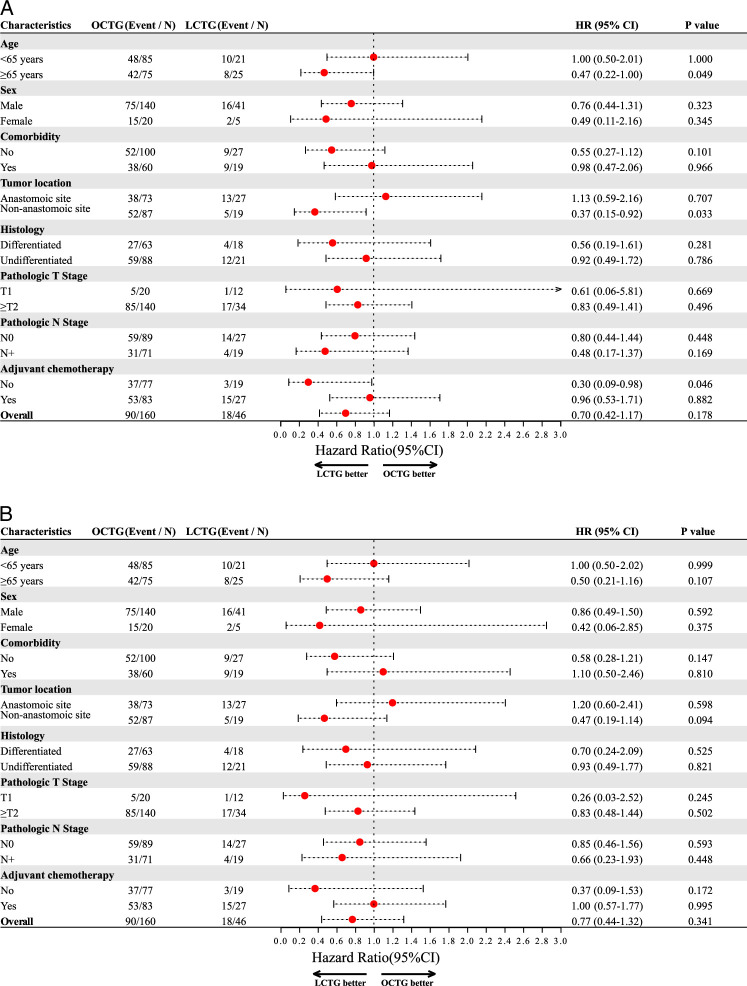
Subgroup analysis of association between disease-free survival and LCTG. (A) Univariate analysis. (B) Multivariate analysis adjusted for age, sex, comorbidities, tumor location, histology, pathological T and N stages, and adjuvant chemotherapy.

Figure 2 illustrates the subgroup analysis of DFS between the LCTG and OCTG groups, demonstrating comparable DFS in each subgroup (all *P*>0.05) (Fig. 2A, B). Similar trend was observed in subgroup analyses of OS and RFS (eFigure 7, Supplemental Digital Content 12, http://links.lww.com/JS9/C366 and 8, Supplemental Digital Content 13, http://links.lww.com/JS9/C367).

#### Per-protocol analysis

In per-protocol analysis, the incidence of overall or specific types of complications was also similar between the two groups (*P*>0.05; eTable 14, Supplemental Digital Content 4, http://links.lww.com/JS9/C358). Intraoperative blood loss and postoperative recovery (time to removal of intra-abdominal drains, time to initiation of solid food intake, time to flatus, and postoperative hospital stay) in the LCTG group were significantly better than those in the OCTG group (all *P*<0.05; eTable 15, Supplemental Digital Content 4, http://links.lww.com/JS9/C358). The 3-year DFS was similar between the LCTG and OCTG groups before and after IPTW (log-rank *P*=0.176; eFigure 9A, C, Supplemental Digital Content 14, http://links.lww.com/JS9/C368). Similarly, the 3-year OS of the LCTG group was comparable to that of the OCTG group (log-rank *P*=0.307; eFigure 9B, D, Supplemental Digital Content 14, http://links.lww.com/JS9/C368). Additionally, the cumulative hazard was comparable between the two groups for overall recurrence or any specific recurrence type (eFigure 9E, L, Supplemental Digital Content 14, http://links.lww.com/JS9/C368).

## Discussion

Most previous studies on RGC were limited to case reports or small-sample retrospective studies, which were mainly comprised by early-stage cancer cases^4,17,23,26^. Thus, high-level evidence regarding the application of laparoscopic surgery for RGC remains scarce. The FUGES-004 trial is the first prospective cohort study to report the safety, feasibility, and long-term efficacy of LCTG for treating RGC. Over a 3-year follow-up, LCTG exhibited less intraoperative blood loss, faster postoperative recovery, and comparable long-term prognosis than those of OCTG. These results indicated promising clinical prospects of this relatively complicated procedure.

With the growing acceptance of minimally invasive surgery, surgeons have been exploring the use of laparoscopic techniques for the radical resection of RGC. However, LCTG is a relatively complicated procedure that requires higher skills and its safety and feasibility remain controversial. Previous studies have reported 20–42% complication rates with LCTG^4,25,26,33,36,44–46^. In this study, the complication rates in both the groups exceeded 25%, consistent with previously reported rates. The dense adhesion between the remnant stomach and liver’s lateral segment, as well as potential adhesion to the pancreatic head after reconstruction, can make distinguish between adhesion and tumor invasion more challenging. Moreover, improper surgical procedures may cause accidental damage and complications, such as pancreatic fistula or abdominal infection^47^. Furthermore, multiviceral resection is necessary when advanced RGC invades other surrounding organs, which increases surgical trauma and operation duration. This would aggravate systemic immune and inflammatory responses, potentially leading to higher incidences of postoperative complications, such as pneumonia. Although abdominal adhesions make LCTG challenging, many researchers remain hopeful that minimally invasive surgery can improve short-term outcomes through precise anatomical dissection^26,28^. A recent retrospective study reported lower overall and severe postoperative complication rates in patients with RGC who underwent minimally invasive surgery^17^. Consistent with the present study, previous studies^4,18^ have demonstrated that LCTG is associated with less intraoperative blood loss, fast postoperative recovery, and short hospital stay, suggesting that LCTG offer advantages in terms of short-term outcomes, facilitating rapid recovery of patients with RGC. We also observed a higher rate of achieving TO in the LCTG group, indicating that LCTG can enhance quality control and standardize treatment for RGC. These advantages could be attributed to the following reasons. LCTG provided surgeons with a magnified view and stable maneuverability, facilitating meticulous dissection, particularly in difficult-to-access areas, such as the perisplenic region. Additionally, LCTG may improve patient compliance with and tolerance for postoperative adjuvant chemotherapy, potentially improving clinical outcomes.

Regarding long-term oncological efficacy, several retrospective studies have reported comparable OS rates between the LCTG and OCTG groups^4,18,21,22,25,26^. However, these studies were limited by sample sizes and short follow-up durations (less than 3-year), making the results less reliable and robust for further clinical application. Therefore, we evaluated the long-term oncological efficacy of LCTG to confirm its noninferiority. And we also observed that the OS, DFS, and cumulative recurrence hazard were comparable between LCTG and OCTG. Additionally, Aoyama *et al*.^17^ reported no significant differences in the recurrence pattern between LCTG and OCTG. This trend was validated by our study with a long-term surveillance, moreover, we also observed comparable mean recurrence time between the two groups. Stratified analyses further confirmed comparable DFS, OS, and RFS rates between the two groups in each subgroup, indicating that LCTG can achieve a similar degree of radicality as that of OCTG without affecting the recurrence pattern or long-term survival. Although the prognosis of LCTG was comparable to that of OCTG, RGC still exhibited poorer prognosis than that of primary gastric cancer due to differences in biological behaviors, cancer stages, and a lower radical gastrectomy rate^48,49^. Therefore, more attention should be paid to the recurrence surveillance, and standardized adjuvant therapy should be emphasized after LCTG.

To reduce time bias, a concurrent comparison was conducted in this study. After concurrent comparison, the differences in operation time and number of harvested lymph nodes between the two groups lost their significance. This could be attributed to the learning curve associated with surgical skills. In the early period, surgeons had insufficient experience with radical resection of RGC (both LCTG and OCTG), resulting in long operation time and insufficient number of harvested lymph nodes. However, after overcoming the learning curve, operation times decreased and the number of harvested lymph nodes increased. When this prospective study was launched, surgeons had already overcome the learning curve, performing ≥100 laparoscopic gastrectomies annually. With the well-established surgical procedures and expertise, surgeons could perform both LCTG and OCTG with similar proficiency. Other short-term outcomes of the concurrent comparisons were similar to those of the above-mentioned results, suggesting that in experienced centers, LCTG is safe and feasible for treating RGC.

This study had several limitations. First, due to the low incidence of RGC, conducting large-scale randomized controlled clinical was challenging. To reduce bias, we used a multicenter cohort from the CRGC-01 study as a historical control and employed IPTW to minimize clinicopathological characteristics differences between the LCTG and OCTG groups, which can simulate the design of RCT as much as possible, so as to obtain more reliable results and conclusions. Likewise, several previous high-quality studies adopted the same method^50–55^, which can solve the relevant clinical issue in some extend without increasing additional risks to patients or increasing extra medical expenses, thus maximizing the utilization of existing research data^56,57^. Additionally, we performed a concurrent comparison to address the time bias from the historical control. Second, although this represents the first prospective cohort study to explore the efficacy of LCTG for RGC, the sample size was relatively small because of its phase II clinical exploratory study design. Therefore, large-scale randomized controlled clinical trials are required. Third, since the OCTG group is a historical control cohort, it is hard to accurately evaluate the operator level and operation difficulty. It is meaningful to explore the impact of the operation level and operation difficulty on the short-term and long-term efficacy of RGC in the future studies. Finally, because RGC are rare, and most LCTGs are performed in experienced high-volume centers, the conclusions of this study should be reappraised when extrapolated to other centers. Even so, this is the first prospective study with a long follow-up period to confirm the safety and feasibility of LCTG for treating RGC.

## Conclusion

In conclusion, compared with OCTG, LCTG demonstrated comparable postoperative complications and long-term oncological efficacy, as well as enhanced the postoperative rehabilitation process in patients with resectable RGC. In high-volume centers, LCTG is recommended as a treatment option for these patients. However, large-scale, prospective, randomized controlled trials are required to further validate our study findings.

## Ethical approval

This study was approved by the institutional review board of FJMUUH (IRB number: 2016YF009-02).

## Consent for publication

This article does not report an individual participant’s data in any form.

## Sources of funding

This study was supported by Construction Project of Fujian Province Medical ‘Create Double High’ ([2021]76).

## Author contribution

C.-M.H., J.-X.L., Q.Z., and D.W.: conception/design; Q.Z., Y.J., Q.-L.H., X.-Y.D., D.-B.X., Y.S., G.-Q.S., K.-Q.G., H.Z., L.-S.C., W.Y., G.L., P.L., J.-W.X., Q.-Y.C., and J.L.: collection and/or assembly of data; Q.Z., D.W., Y.J., C.-M.H., and J.-X.L.: data analysis and interpretation; D.W., Q.Z., J.-X.L., and C.-M.H.: manuscript writing.

## Conflicts of interest disclosure

All authors declare that they have no conflict of interest.

## Research registration unique identifying number (UIN)

1. Name of the registry: Clinical Trials.gov.
2. Unique identifying number or registration ID: NCT02792881.
3. Hyperlink to your specific registration (must be publicly accessible and will be checked): https://classic.clinicaltrials.gov/ct2/show/NCT02792881?term=NCT02792881&draw=2&rank=1

## Guarantor

Jian-Xian Lin.

## Availability of data and materials

The datasets used and/or analyzed during the current study are available from the corresponding author on reasonable request.

## Human rights statement and informed consent

All procedures followed were in accordance with the ethical standards of the responsible committee on human experimentation (institutional and national) and with the Helsinki Declaration of 1964 and later versions. Informed consent or substitute for it was obtained from all patients for being included in the study.

## Supplementary Material
