# Supplementary material for: The safety, feasibility, and oncological outcomes of laparoscopic completion total gastrectomy for remnant gastric cancer: a prospective study with 3-year follow-up (FUGES-004 study)
Source: Int J Surg. 2024 Apr 9;110(6):3382–91. doi: 10.1097/JS9.0000000000001388 (PMC11175827; doi:10.1097/JS9.0000000000001388)
Supplement: Supplementary file 1 [file js9-110-3382-s001.pdf]

The Safety, Feasibility, and Oncological Outcomes of Laparoscopic Completion Total Gastrectomy for Remnant Gastric Cancer: A Prospective Trial (FUGES-004)

Study protocol

Research center: Fujian Medical University Union Hospital

Principle Investigator:

Prof. Chang-Ming Huang, M.D.

Department of Gastric Surgery, Fujian Medical University Union Hospital,

Address: No. 29 Xinquan Road, Fuzhou 350001 Fujian Province, China.

Telephone: +86-591-83363366, Fax: +86-591-83363366

E-mail: hcmlr2002@163.com

No. of edition: V1.3 (Latest update date: 2020.06.30)

Summary

Protocol Title	The Safety, Feasibility and Oncological Outcomes of Laparoscopic Completion Total Gastrectomy for Remnant Gastric Cancer: A Prospective Trial (FUGES-004)
Protocol Version	V1.3
PI	Chang-Ming Huang
Research Centers	Fujian Medical University Union Hospital
Indications	Patients with remnant gastric cancer (cT1-4a, N+/-, M0)
Research purpose	To investigate the safety and feasibility of laparoscopic completion total gastrectomy (LCTG) for remnant gastric cancer (RGC)
Research Design	Single center, prospective, open-label, single arm
Case Grouping	Study group: LCTG with D2 lymphadenectomy
Determination of Sample Size	According to the retrospective study, the complication rate of LCTG in our center was 15.1% (expected value) [1], whereas the incidence of complication of open completion total gastrectomy (OCTG) for RGC was about 34.3% (target value) [2-4]. Therefore, the threshold level was set as 34.3%, and the expected level was set as 15.1%. Based on a one-sided alpha of 0.05 and a power of 80%, at least 38 patients needed to be enrolled for this phase II clinical trial. Considering an expected dropout rate of 20%, the sample size required for the study group were 50 cases. The sample size was calculated using nQuery Advisor 7.0 (Statistical Solutions, Cork, Ireland).
Inclusion Criteria	● Age between 18 and 75 years<br>● Carcinomas arise in the remnant stomach following distal gastrectomy, irrespective of the histology of the primary lesion (benign or malignant) or its risk of recurrence, the extent of resection, or method of reconstruction (Billorth-I or Billorth-II).

	● cT1-4a, N-/+, M0 at preoperative evaluation according to the AJCC Cancer Staging Manual, 7th Edition<br>● Performance status of 0 or 1 on Eastern Cooperative Oncology Group scale (ECOG)<br>● American Society of Anesthesiology score (ASA) class I, II, or III<br>● Underwent radical LCTG with D2 lymphadenectomy<br>● Written informed consent
Exclusion Criteria	● Distant metastasis in the preoperative examinations<br>● Previous upper abdominal surgery (except laparoscopic cholecystectomy, previous gastrectomy, endoscopic mucosal resection or endoscopic submucosal dissection)<br>● Other malignant disease (except gastric cancer) within the past 5 years<br>● Enlarged or bulky regional lymph node (diameter over 3cm) supported by preoperative imaging<br>● Women during pregnancy or breast-feeding<br>● Severe mental disorder<br>● Unstable myocardial infarction, angina, or cerebrovascular accident within the past 6 months<br>● History of continuous systematic administration of corticosteroids within one month<br>● FEV1<50% of predicted values<br>● Requirement of simultaneous surgery for other disease<br>● Emergency surgery due to complication (bleeding, obstruction or perforation) caused by gastric cancer
Withdraw criteria	● M1 tumor confirmed intraoperatively or postoperatively: distant metastasis only found by intraoperative exploration or postoperative pathological biopsy or a positive postoperative peritoneal lavage cytology examination.

	● Patients intraoperatively confirmed as unable to complete D2 lymph node dissection/R0 resection due to tumor: unable to complete R0 resection due to regional lymph node integration into a mass or surrounded with important blood vessels, which cannot be resected<br>● Patients converted to OCTG intraoperatively<br>● Simultaneous surgical treatment of other diseases<br>● Sudden severe complications during the perioperative period (intolerable surgery or anesthesia), which renders it unsuitable or unfeasible to implement the study treatment protocol as scheduled<br>● Patients confirmed to require emergency surgery by attending physicians due to changes in the patient's condition after inclusion in this study<br>● Patients who voluntarily quit or discontinue treatment for personal reasons at any stage after inclusion in this study<br>● Treatment implemented is proven to violate study protocol
Intervention	LCTG with D2 lymph node dissection
Endpoints	**Primary Outcome Measures**：<br>● Overall postoperative morbidity rates<br>**Secondary Outcome Measures**：<br>● 3-year disease free survival rate<br>● 3-year overall survival rate<br>● 3-year recurrence pattern<br>● Mortality<br>● Rates of combined organ resection<br>● Intraoperative morbidity rates<br>● Number of retrieved lymph nodes<br>● Operation time<br>● Postoperative recovery course<br>● Intraoperative blood loss

	● Inflammatory and immune response<br>● Textbook Outcome<br>● Technical performance<br>● The Surgery Task Load Index (SURG-TLX)
Statistical Considerations	All data analyses will be performed using SPSS statistical software, version 18.0 (SPSS Inc), and the R software environment (R Foundation for Statistical Computing).<br>All the statistical tests were tested by two sides. A p-value <0.05 is considered statistically significant. The confidence interval of the parameters is estimated with a 95% confidence interval. Baseline data and validity analyses will be conducted on a modified intent-to-treat (MITT) basis, and the primary endpoint will also be analyzed on a per-protocol (PP) basis, but the conclusions of the PP analysis were the main ones. SAP analysis is used for safety assessment, and this study does not fill in missing values. Normally distributed continuous variables will be presented as mean and standard deviation and compared using the t-test if normally distributed, or as median and interquartile range and compared using the Wilcoxon rank-sum test if non-normally distributed; while categorical data will be presented as number and percentages and compared using the Pearson $\chi 2$ test or the Fisher exact test, as appropriate. Survival data will be analyzed using the Kaplan-Meier method and Cox's proportional hazards model. The historic control cohort will be added as comparison. Additionally, subgroup analyses and concurrent comparisons were performed to eliminate grouping and time bias between LCTG and OCTG, respectively. Sensitivity analysis is used for extreme outlier data. The central effect analysis and subgroup analysis are conducted according to the specific situation. Interim analysis will not be conducted in this study.

1. Research background

Nearly 103 million new cases and 782,685 deaths due to gastric cancer occur annually worldwide[5]. The incidence of gastric cancer in China was 67.9/100,000 in 2015, more than 60% of all gastric cancer cases globally, with a mortality rate of up to 48.9/100,000, making gastric cancer the second most common malignant tumour and the third leading cause of tumour-related deaths[6]. For patients with a history of gastric surgery, the incidence of remnant gastric cancer (RGC) is approximately 2-3% and accounts for 1.8% of all gastric malignancies[7-9]. In recent years, the incidence of remnant gastric cancer has been increasing, which is mainly due to the prolonged survival of patients with previous gastrectomy for gastric ulcer and primary gastric cancer [10]. With the popularity of endoscopic screening, more patients with RGC were detected at the early stage. RGC has gradually become a major public health concern.

In 2005, since Yamada reported the first case of laparoscopic completion total gastrectomy (LCTG) for RGC[11], laparoscopic technology was widely used in the treatment of RGC. However, due to the low incidence and rapid aggravation of RGC [12], and adhesion, anatomic changes and more complex lymphatic pathway caused by the previous operation [9,13], the popularity of LCTG is limited. So far, only a few retrospective studies have demonstrated the feasibility and safety of LCTG for RGC [2,3,14-17]. Nagai et al. [15] reported that LCTG was feasible and safe in the treatment of RGC. Subsequently, Kim et al. [2] also found the similar results. However, the above reports are retrospective studies and the sample sizes are relatively small. Additionally, because lacking of prospective studies and technical difficulties in performing such operations, the application of LCTG still remains controversy in these patients. Therefore, higher level evidence is warrant to further confirm the safety, feasibility and oncological outcomes of LCTG in patients with RGC.

2. Objective

The purpose of this prospective trial is to investigate the safety, feasibility and Oncological Outcomes of LCTG for remnant gastric cancer (cT1-T4a).

3. Research design

Single center, prospective, open-control, Phase 2, single-arm,

3.1 Single center

Department of gastric surgery in Fujian Medical University Union Hospital

3.2 Case group

Group A (Study Group): LCTG Group

3.3 Estimate sample size

According to the retrospective study, the complication rate of LCTG in our center was 15.1% (expected value) [1], whereas the incidence of complication of open completion total gastrectomy (OCTG) for RGC was about 34.3% (target value) [2-4]. Therefore, the threshold level was set as 34.3%, and the expected level was set as 15.1%. Based on a one-sided alpha of 0.05 and a power of 80%, at least 38 patients needed to be enrolled for this phase II clinical trial. Considering an expected dropout rate of 20%, the sample size required for the study group were 50 cases. The sample size was calculated using nQuery Advisor 7.0 (Statistical Solutions, Cork, Ireland).

3.4 Blind method： This research adopts an open design

3.5 Research cycle

Estimated enrollment cycle: complete enrollment within 5 years

Follow-up period: begin at the enrollment of the first case and end 3 years after the enrollment of the last case.

Actual time: 2016.06-2020.06 (to complete enrollment)-2023.09 (to complete follow-up)

4. Study objects

All patients who meet the inclusion criteria and not conform to the exclusion criteria are qualified for this study.

4.1 Inclusion criteria

(1) Age between 18 and 75 years

(2) Carcinomas arise in the remnant stomach following distal gastrectomy, irrespective of the histology of the primary lesion (benign or malignant) or its risk of recurrence, the extent of resection, or method of reconstruction (Billorth-I or Billorth-II)

(3) cT1-4a, N-/+, M0 at preoperative evaluation according to the AJCC Cancer Staging

Manual, 7th Edition

(4) Performance status of 0 or 1 on Eastern Cooperative Oncology Group scale (ECOG)

(5) American Society of Anesthesiology score (ASA) class I, II, or III

(6) Underwent radical LCTG with D2 lymphadenectomy

(7) Written informed consent

4.2 Exclusion criteria

(1) Distant metastasis in the preoperative examinations

(2) Previous upper abdominal surgery (except laparoscopic cholecystectomy, previous gastrectomy, endoscopic mucosal resection or endoscopic submucosal dissection)

(3) Other malignant disease (except gastric cancer) within the past 5 years

(4) Enlarged or bulky regional lymph node (diameter over 3cm) supported by preoperative imaging

(5) Women during pregnancy or breast-feeding

(6) Severe mental disorder

(7) Unstable myocardial infarction, angina, or cerebrovascular accident within the past 6 months

(8) History of continuous systematic administration of corticosteroids within one month

(9) FEV1<50% of predicted values

(10) Requirement of simultaneous surgery for other disease

(11) Emergency surgery due to complication (bleeding, obstruction or perforation) caused by gastric cancer

4.3 Withdraw criteria

(1) M1 tumor confirmed intraoperatively or postoperatively: distant metastasis only found by intraoperative exploration or postoperative pathological biopsy or a positive postoperative peritoneal lavage cytology examination

(2) Patients intraoperatively confirmed as unable to complete D2 lymph node dissection/R0 resection due to tumor: unable to complete R0 resection due to regional lymph node integration into a mass or surrounded with important blood vessels, which cannot be resected

(3) Patients converted to OCTG intraoperatively

(4) Simultaneous surgical treatment of other diseases

(5) Sudden severe complications during the perioperative period (intolerable surgery or anesthesia), which renders it unsuitable or unfeasible to implement the study treatment protocol as scheduled

(6) Patients confirmed to require emergency surgery by attending physicians due to changes in the patient's condition after inclusion in this study

(7) Patients who voluntarily quit or discontinue treatment for personal reasons at any stage after inclusion in this study

(8) Treatment implemented is proven to violate study protocol

4.4 Case screening

(1) When Patients admitted to hospital should meet the following criteria: Age between 18 and 75 years old; Performance status of 0 or 1 on the ECOG scale;None-pregnant or no lactating women; Not suffering from a severe mental disorder; No history of previous upper abdominal surgery (except for laparoscopic cholecystectomy and ESD/EMR/gastrectomy for gastric cancer); No History of other malignant disease (except for gastric cancer) within the past five years; No history of unstable angina or myocardial infarction within the past six months; No history of continuous systematic administration of corticosteroids within one month; No requirement of simultaneous surgery for another disease; FEV1≥50% of the predicted values; No history of a cerebrovascular accident within the past six months.

(2) Endoscopic examination of the primary lesion in the patient (ultrasound endoscopy is recommended) and histopathological biopsy showed gastric adenocarcinoma (papillary adenocarcinoma [pap], tubular adenocarcinoma [tub], mucinous adenocarcinoma [muc], signet ring cell carcinoma [sig], and poorly differentiated adenocarcinoma [por]). Total abdominal CT was performed on the patient, and no enlarged lymph nodes (maximum diameter ≥ 3 cm) were found in the periplasmic area or local invasion or distant metastasis.

(3) Patient is explicitly diagnosed with remnant gastric cancer, has a preoperative clinical staging assessment of T1-4a, N0-3, M0 and is expected to undergo laparoscopic

total gastrectomy and D2 lymph node dissection to obtain R0 surgical results.

(4) ASA class I to III.

(5) No requirement for emergency surgery.

(6) At this point the patient becomes a potential selected case and enters the 9.1 case selection procedure.

5. Outcome Measures

5.1 Primary Outcome Measures

- Overall postoperative morbidity rates

Secondary Outcome Measures:

- 3-year disease free survival rate
- 3-year overall survival rate
- 3-year recurrence pattern
- Mortality
- Rates of combined organ resection
- Intraoperative morbidity rates
- Number of retrieved lymph nodes
- Operation time
- Postoperative recovery course
- Intraoperative blood loss
- Inflammatory and immune response
- Textbook Outcome
- Technical performance
- The Surgery Task Load Index (SURG-TLX)

6. Diagnostic criteria for this study

(1) The AJCC-7th TNM tumor staging system will be used for this study.

(2) Diagnostic criteria and classification of gastric cancer: According to the histopathological international diagnostic criteria, classification will be divided into papillary adenocarcinoma (pap), tubular adenocarcinoma (tub), mucinous adenocarcinoma (muc), signet ring cell carcinoma (sig), and poorly differentiated

adenocarcinoma (por).

(3) Definition of remnant gastric cancer: carcinomas arising in the remnant stomach following gastrectomy, irrespective of the histology of the primary lesion (benign or malignant) or its risk of recurrence, the extent of resection, or method of reconstruction.

7 Qualifications of the participated Surgeons

7.1 Basic principle

All candidate surgeons in this study met the following criteria:

Performed at least 50 laparoscopic total gastrectomies.

Pass the blind surgical video examination.

7.2 Checklist for determination of success about D2 lymphadenectomy [18]

Scoring Method for D2 Lymph Node Dissection (if available, not all following steps are included in the LCTG for patients with RGC)	Complete 10	Incomplete 5	None 0
1. Properly full omentectomy	□	□	□
2. Ligation of left gastroepiploic artery at origin	□	□	□
3. Ligation of right gastroepiploic artery at origin	□	□	□
4. Full exposure of common hepatic artery	□	□	□
5. Ligation of right gastric artery at origin	□	□	□
6. Exposure of portal vein	□	□	□
7. Exposure of splenic artery to branch of posterior gastric artery	□	□	□
8. Identification of splenic vein	□	□	□
9. Ligation of left gastric artery at origin	□	□	□
10. Exposure of gastroesophageal junction	□	□	□

1. Properly full omentectomy
   a. Omentectomy was performed close to transverse colon
   b. Omentectomy was performed from hepatic flexure to splenic flexure
   c. Anterior layer of transverse colonic mesentery and pancreatic anterior peritoneum was dissected.
2. Ligation of left gastroepiploic artery at origin
3. Ligation of right gastroepiploic artery at origin
4. Full exposure of common hepatic artery
   a. More than half of anterior part in the common hepatic artery were exposed.
5. Ligation of right gastric artery at origin
6. Exposure of portal vein

7. Exposure of splenic artery
   a. Anterior part in splenic artery was exposed.
   b. Splenic artery was exposed from celiac trunk to the terminal branch of the splenic artery (if available)
8. Identification of splenic vein
9. Ligation of left gastric artery at origin
10. Exposure of gastroesophageal junction
   a. Anterior, posterior, left and right side of the abdominal esophagus were exposed.

- D2 lymphadenectomy was accepted if all randomly assigned three investigators rated more than 80% of total points regarding checklists in unedited video review.

8. End point and definition of related result determination

8.1 Incidence of operative complications

The number of all patients treated with surgery as the denominator and the number of the patients with any intraoperative and postoperative complications as the numerator are used to calculate the proportions. The criteria for the intraoperative complications refer to the descriptions of intraoperative complications in the observation project (in **9.3.3**).

8.1.1 Incidence of intraoperative complications

With the number of patients undergoing surgery as the denominator, the number of patients with any of the following intraoperative complications is calculated as numerator. Intraoperative complications are based on the intraoperative complications mentioned in the intraoperative observations (in **9.3.3**).

8.1.2 Incidence of postoperative complications

The number of all patients treated with surgery as the denominator and the number of the patients with any postoperative complications as the numerator are used to calculate the proportions.

8.1.3 Incidence of overall postoperative complications: The postoperative complication criteria refer to short-term complications after surgery in the postoperative observation project (see 9.4.5). The time is defined as within 30 days after surgery, or the first discharge time if the days of hospital stay more than 30 days.

8.1.4 Incidence of postoperative major complications: The standard for postoperative major complications refers to the short-term complications in the postoperative observation project (see 9.4.5) according to the Clavien–dindo grade, IIIA level and above for severe complications, and when multiple complications occur simultaneously, the most severe one is recorded.

8.2 Mortality

• The number of all the patients receiving surgery as the denominator and the number of the patients in any of the following situations as the numerator are used to calculate proportions. This proportion indicated the operative mortality ratio.

• Situations: patients whose death was identified according to documented intraoperative observation items, including patients who die within 30 days after the surgery (including 30 days) regardless of the causality between the death and the surgery, and patients who die more than 30 days after the surgery (whose death is proved to have a direct causal relationship with the first operation).

8.3 Disease-free survival

Disease-free survival is calculated from the day of surgery to the day of recurrence or death (When the specific date of recurrence of the tumor is unknown, the ending point is the date of death due to tumor causes). In the event that neither death nor recurrence of the tumor are observed, the end point is the final date that a patient is confirmed as relapse-free. (The final date of DFS： The last date of the outpatient visit day or the date of acceptance of the examination).(Follow-up cycle and required examinations are shown in the follow-up process 9.5.3)

8.4 Overall survival

The overall survival is calculated from the day of surgery until death or until the final follow-up date, whichever occurs first. For survived cases, the end point is the last date that survival was confirmed. If loss to follow-up occurred, the end point is the final date that the survival status could be confirmed.

8.5 Definition of recurrence and recurrence date

The following situations are regarded as "recurrence" and should be recorded as the evidence of "recurrence" in the CRF.

(1) Recurrence identified by any one image examination (X-ray, ultrasound, CT, MRI, PET-CT, endoscopy, etc.) and, if there are a variety of imaging examinations, results without contradiction determined "recurrence". The earliest date that the recurrence is found is defined as the "recurrence date".

(2) For cases that lack the use of imaging or a pathological diagnosis, the date we diagnose the occurrence of clinical recurrence based on clinical history and physical examination is defined as the “recurrence date”.

(3) For cases without imaging or clinical diagnosis but with a cytology or tissue biopsy pathological diagnosis of recurrence, the earliest date confirmed by cytology or biopsy pathology is considered the "recurrence date".

(4) A rise in CEA or other associated tumor markers alone could not be diagnosed as a relapse.

8.6 Determination of surgical outcomes

8.6.1 Operative time: from skin incision to the skin being sutured

8.6.2 Postoperative recovery indexes

8.6.2.1 Time to ambulation, flatus, recovery of liquid diet and semi-liquid diet.

- During the day of surgery to the first discharge, the initial time to ambulation, flatus, liquid diet and semi-liquid diet during the postoperative hospitalization is recorded by hour.
- Flatus on the operation day should be excluded.
- If flatus or resumption of liquid and semi-liquid diet does not occur before hospital discharge, the discharge time should be recorded as the corresponding time.
- The initial time to ambulation, flatus, liquid diet and semi-liquid diet should be recorded according to patients' reports.

8.6.2.2 The maximum temperature

The highest value of body temperature measured at least 3 times a day from the first day to the eighth day after operation is documented.

8.6.3 Percentage of conversion to laparotomy

Among all the patients who underwent surgery, the number of patients planning to receive a laparoscopic surgery per protocol (PP) is used as the denominator, while the number of the patients who convert to open surgery is considered the numerator. The proportion calculated is regarded as the rate of transfer laparotomy. In this study, if the length of the auxiliary incision is more than 10 cm, it is considered a conversion to open surgery.

8.7 Textbook Outcome

Textbook outcome (TO)[19] was defined based on review of existing TO metrics in the literature including outcomes such as complete-potentially curative status, no intraoperative complications, no eventful postoperative complications (Clavien-Dindo grade III or higher)[20-21], 15 lymph nodes(LNs) examined, hospital stay < 21days, no

reintervention (surgical, endoscopic or radiological) within 30 days after surgery, no readmission to the intensive care unit (ICU) within 30 days after surgery, no postoperative mortality within 30 days after surgery, and no hospital readmission within 30 days after discharge. When all nine desired health outcomes were realized, TO was achieved.

8.8 Technical performance

Technical performance was assessed by the Objective Structured Assessments of Technical Skills (OSATS) [22] and the Generic Error Rating Tool(GERT)[23]. Detailed global 5-point rating scale for OSATS was shown in the following scale:

**Respect for tissue**	1<br>Frequently used unnecessary force on tissue or caused damage by inappropriate use of instruments.	2	3<br>Careful handling of tissue but occasionally caused inadvertent damage.	4	5<br>Consistently handled tissues appropriately with minimal damage.
**Time and motion**	1<br>Many unnecessary moves.	2	3<br>Efficient time/motion but some unnecessary moves.	4	5<br>Economy of movement and maximum efficiency.
**Instrument handling**	1<br>Repeatedly makes tentative or awkward moves with instruments.	2	3<br>Competent use of instruments although occasionally appeared stiff or awkward.	4	5<br>Fluid moves with instruments and no awkwardness.
**Knowledge of instruments**	1<br>Frequently asked for the wrong instrument or used an inappropriate instrument.	2	3<br>Knew the names of most instruments and used appropriate instrument for the task.	4	5<br>Obviously familiar with the instruments required and their names.
**Use of assistants**	1<br>Consistently placed assistants poorly or failed to use assistants.	2	3<br>Good use of assistants most of the time.	4	5<br>Strategically used assistant to the best advantage at all times.
**Flow of operation and forward planning**	1<br>Frequently stopped operating or needed to discuss next move.	2	3<br>Demonstrated ability for forward planning with steady progression of operative procedure.	4	5<br>Obviously planned course of operation with effortless flow from one move to the next.
**Knowledge of specific procedure**	1<br>Deficient knowledge. Needed specific instruction at most operative steps.	2	3<br>Knew all important aspects of the operation.	4	5<br>Demonstrated familiarity with all aspects of the operation.

Objective assessment of technical ability of the primary surgeon using the GERT was conducted for the following five main steps of the operation: (1) exploration and separation of adhesions and the greater omentum; (2) dissection of the lymph nodes (LNs) in the suprapancreatic area; (3) exposing the right side of the esophagus; (4) exposing the left gastroepiploic vessels and dissection of the LNs in the splenic hilar area; and (5) exposing the left side of the esophagus.

Surgical procedure	Start (S) and endpoint (E)	Standard skills and tasks observed
**Preoperative Exploration**	Preoperative diagnostic laparoscopy	Assess the extent of intra-abdominal adhesions
**Removal the the Adhesions and Greater Omentum**	S: Identification of the perigastric adhesions and the superior border of the transverse colon near its midpoint<br>E: Completely separating the perigastric adhesions and the attachment of the greater omentum and transverse colon	**Grasping:** the adhesions, the omentum, the transverse colon, and the bowel<br>**Pressing:** stomach and transverse mesocolon<br>**Finding fascia:** the superior border of the transverse colon near its midpoint<br>**Use of energy device/Dissection:**<br>a.the adhesions between the omentum, the small and large bowels, the abdominal wall, and other intra-abdominal organs.<br>b.the greater omentum
**Mobilize the duodenum (only for patients who underwent Billroth type I reconstruction previously).**	Fully bare the wall of duodenum	**Grasping:** the omentum and the duodenum<br>**Pressing:** pancreas<br>**Use of energy device/Dissection:** the omentum and the hepatoduodenal ligament
**Laparoscopic Suprapancreatic Area Lymph Node Dissection for Remnant Gastric Cancer**	S: Opening the left gastropancreatic fold to enter the retropancreatic space<br>E: Completely excise the gastrohepatic ligament	**Grasping:** the lesser omentum, the gastropancreatic fold, the fatty lymphatic tissue, the free pancreatic capsule<br>**Pressing:** pancreas
**Exposing the right side of the esophagus**	S:Separation of diaphragmatic feet<br>E:Exposure of the right side of esophageal hiatus	**Finding fascia:** a. the initial segment of the splenic artery; b. the retropancreatic space; c. the avascular space on the surface of the left and right crura of the diaphragm; d. the origin of the proper hepatic artery; e. retrogastric intrafascial space<br>**Revealing and dissecting the vessels:** the proximal splenic artery, the celiac trunk, the coronary vein, the left gastric artery, the common hepatic artery, the proper hepatic artery (if available)<br>**Cutting:** the coronary vein, the left gastric artery, the right gastric vessels in this area<br>**Use of energy device/Dissection:** the No. 11p, 7, 9, 8a, 12a, 1, and 3 LNs (all or part) LNs

		**Transection and stapling:** using an endoscopic linear stapler to transect the duodenum (only for patients who underwent Billroth-I reconstruction previously).
Laparoscopic Splenic Hilar Area Lymph Node Dissection for Gastric Cancer	**First step: dissection of the LNs in the inferior pole region of the spleen**	**Grasping:** the free greater omentum, the fundus of stomach, the fatty lymphatic tissue
	S: Separation of the gastrosplenic ligament using the ultrasonic scalpel	**Pressing:** pancreas, the vessels of the splenic hilar
	E: Transecting one to two branches of the short gastric vessels issuing from the lower lobe vessel of spleen	**Finding fascia:** the retropancreatic space
	**Second step: dissection of the LNs in the region of the splenic artery trunk**	**Revealing and dissecting the vessels:** the left gastroepiploic vessel, the short gastric vessels, the posterior gastric vessel, the splenic artery, the splenic lobar vessels
	S: Denuding the trunk of the splenic artery along the latent anatomic space on its surface toward the splenic hilar	**Clipping:** the left gastroepiploic vessel, the short gastric vessels, the posterior gastric vessel
	E: Reaching the fork of the splenic lobar arteries	**Cutting:** the left gastroepiploic vessel, the short gastric vessels, the posterior gastric vessel
	**Third step: dissection of the LNs in the superior pole region of the spleen**	**Use of energy device/Dissection:** the No. 4sb, 4sa, 11d, and 10 LNs (optional)
	S: Dissection of the fork of the splenic lobar arteries	
	E: Division of the last short gastric vessel	
**Laparoscopic Left Cardia Area Lymph Node Dissection for Gastric Cancer**	**Dissection of No. 2 LNs**	**Grasping:** stomach
	S: Dissecting the gastrosplenic ligament along the diaphragm from the superior pole of the spleen in the direction of the left side of esophageal hiatus	**Pressing:** stomach<br>**Revealing and dissecting the vessels:** the left inferior phrenic artery<br>**Clipping:** the left inferior phrenic artery
	E: Completing the dissection of the No. 2 LNs	**Cutting:** the left inferior phrenic artery
		**Use of energy device/Dissection:** the No. 2 LNs
**Others**	All time periods outside defined steps	**Abdominal access:** trocar insertion and removal
		**Suction:** as required during hemostasis, fogging, splashing
		**Cleaning the Camera:** lens fogging or pollution
		**Clipping:** hemostasis
		**Suturing:** vascular injury

Error Checklist

**Video code**			**Rater code**		
**Surgical task group**	**Error mode**	**Time of observation**	**Total number**	**Event (description/time)**	**Mechanism of event**
Abdominal access	Too much force/distance				
	Too little force/distance				
	Wrong orientation				
	Inadequate visualization				
Finding fascia	Too much force/distance				
	Too little force/distance				
	Wrong orientation				
	Inadequate visualization				
Use of energy devices and dissection	Too much force/distance				
	Too little force/distance				
	Wrong orientation				
	Inadequate visualization				
Revealing and bare the vessels	Too much force/distance				
	Too little force/distance				
	Wrong orientation				
	Inadequate visualization				
Grasping	Too much force/distance				
	Too little force/distance				
	Wrong orientation				
	Inadequate visualization				
Pressing	Too much force/distance				
	Too little force/distance				
	Wrong orientation				
	Inadequate visualization				
Cutting,	Too much force/distance				
	Too little force/distance				
	Wrong orientation				
	Inadequate visualization				
Transection and stapling	Too much force/distance				
	Too little force/distance				
	Wrong orientation				
	Inadequate visualization				
Clipping	Too much force/distance				
	Too little force/distance				
	Wrong orientation				
	Inadequate visualization				
Use of suction	Too much force/distance				
	Inadequate visualization				
Cleaning the camera	Inadequate visualization				
Other unclassified	Description/time:				

8.9 The Surgery Task Load Index

Surgeons were asked to complete one Surg-TLX questionnaire for each procedure in both studies after surgery[24], consisting of 6 subscales addressing mental, physical, and temporal demands, task complexity, situation, and distrations. All questions were rated on a 20-point scale (0 = low, 20 = high).

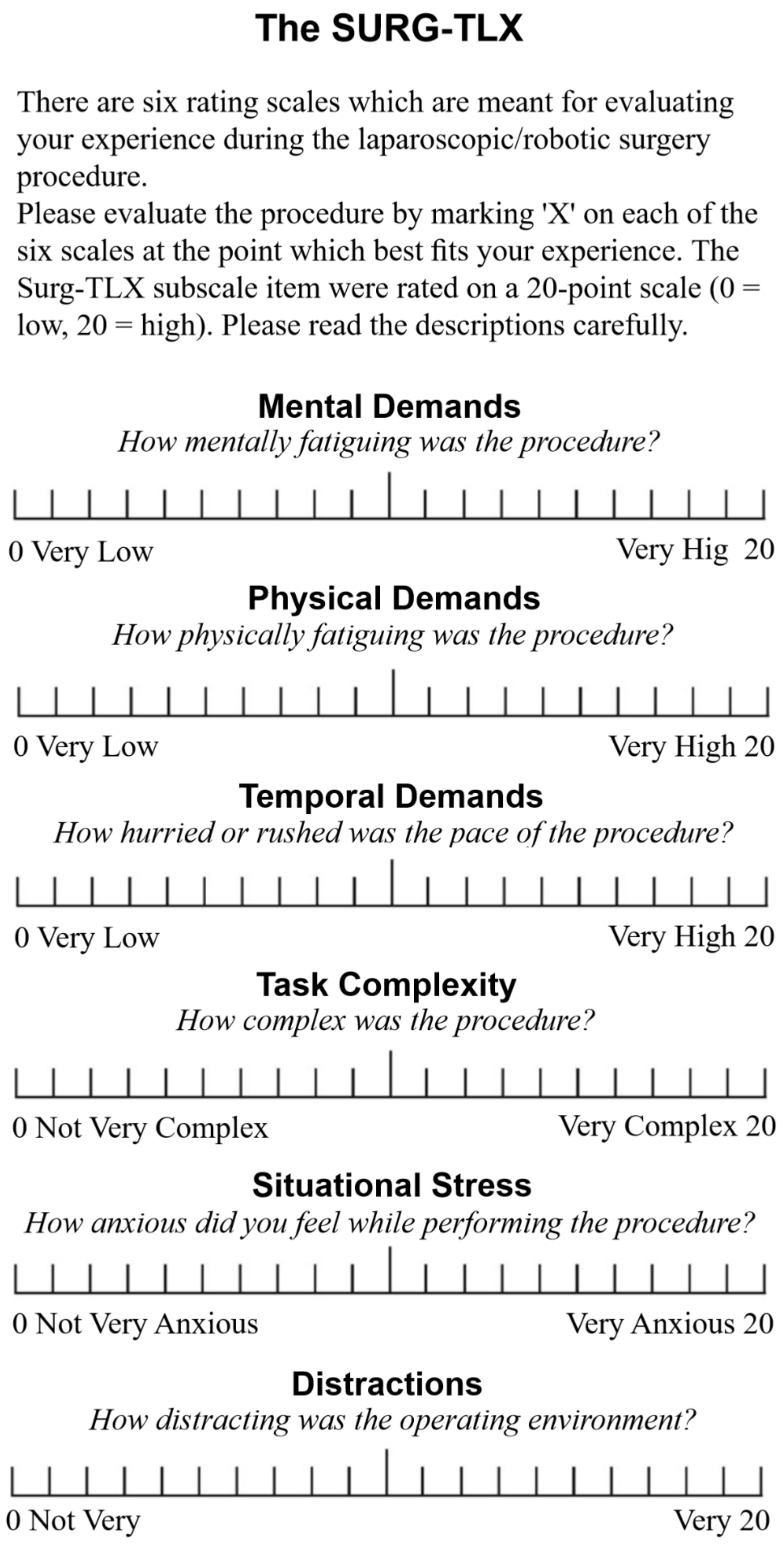

The SURG-TLX

There are six rating scales which are meant for evaluating your experience during the laparoscopic/robotic surgery procedure.
Please evaluate the procedure by marking 'X' on each of the six scales at the point which best fits your experience. The Surg-TLX subscale item were rated on a 20-point scale (0 = low, 20 = high). Please read the descriptions carefully.

Mental Demands
How mentally fatiguing was the procedure?
0 Very Low — Very Hig 20

Physical Demands
How physically fatiguing was the procedure?
0 Very Low — Very High 20

Temporal Demands
How hurried or rushed was the pace of the procedure?
0 Very Low — Very High 20

Task Complexity
How complex was the procedure?
0 Not Very Complex — Very Complex 20

Situational Stress
How anxious did you feel while performing the procedure?
0 Not Very Anxious — Very Anxious 20

Distractions
How distracting was the operating environment?
0 Not Very — Very 20

9 Standard operating procedures (SOP)

9.1 Case selection

9.1.1 Selection assessment items

Clinical examination data of patients conducted from hospital admission to enrollment into this study (time period is usually 2 weeks) will be considered baseline data, and must include:

(1) Systemic status: ECOG score, height, weight

(2) Peripheral venous blood: Hemoglobin, Red blood cell, White blood cell, Lymphocyte, Neutrophils, NEU%, Platelet, Monocyte

(3) Blood biochemistry: albumin, prealbumin, total bilirubin, indirect bilirubin, direct bilirubin, AST, ALT, creatinine, urea nitrogen, Total cholesterol, triglycerides, fasting glucose, potassium, sodium, chlorine, calcium

(4) Serum tumor markers: CEA, CA19-9, CA72-4, CA12-5, AFP

(5) Abdominal CT scan (slice thickness of 10mm or less, in case of allergy to the contrast agent, CT horizontal scanning is allowed only)

(6) Upper gastrointestinal endoscopic ultrasonography (EUS) and biopsy, if no EUS, select ordinary upper gastrointestinal endoscopy and biopsy instead

(7) Chest X-ray (AP and lateral views): cardiopulmonary conditions

(8) Resting 12-lead ECG

(9) Respiratory function tests: FEV1, FVC

9.1.2 Selection application

For cases that meet all inclusion criteria and none of the exclusion criteria, talk to patients and their families and sign informed consent. Application and confirmation of eligibility should be completed preoperatively; postoperative applications will not be accepted.

9.2 Preoperative management

After the eligibility is obtained, surgery should be performed within two weeks (including the 14th day)

- In case of any deterioration of the clinical conditions from the selection time to the expected day of surgery, whether to undergo an elective surgery as planned should be decided in accordance with the judgment of the doctor in charge; if an emergency surgery is required, the case should be withdrawn from PP set according to 4.3 Withdrawal Criteria;

- After the eligibility is obtained, surgery should be performed within two weeks (including the 14th day)
- In case of any deterioration of the clinical conditions from the selected time to the expected day of surgery, whether to undergo an elective surgery as planned should be decided by the judgment of the doctor in charge; if emergency surgery is required, the case should be withdrawn from PP set according to 4.3 Withdrawal Criteria;
- For patients with nutritional risks, preoperative enteral/parenteral nutritional support is allowed.
- For elderly, smokers, high-risk patients with diabetes, obesity, and chronic cardiovascular/cerebrovascular or thromboembolic history, among others, perioperative low-molecular-weight heparin prophylaxis, lower-limb antithrombotic massage, active lower limb massage, training in respiratory function, and other preventive measures are recommended. For other potentially high-risk complications not specified in this study protocol, the doctor in charge can decide on the most appropriate approach according to clinical practice and should record it in the CRF.
- For the operative approach of the surgeries in this study should be selected by the doctor in charge according to his/her experience and the specific intraoperative circumstances.
- Preoperative fasting and water deprivation and other before-anesthesia requirements on patients should follow the conventional anesthesia program, which is not specified in this study.
- For prophylactic antibiotics, the first intravenous infusion should begin 30 minutes before surgery. It is recommended to select a second-generation cephalosporin (there are no provisions on specific brands in this study); the preparation, concentration, and infusion rate should comply with routine practice; and prophylaxis should not exceed postoperative three days at a frequency of one infusion every 12 hours. If a patient is allergic to cephalosporins (including the history of allergy or allergy after cephalosporin administration), other types of antibiotics are allowed according to the specific clinical situation and when used over the same period mentioned.
- Patient data to be collected during the preoperative period also includes CRP.

9.3 Standardization of surgical practice

9.3.1 Principle of Surgical Treatments

9.3.1.1 Anesthesia

The operation is to be carried out with endotracheal intubation under general anesthesia; whether epidural assisted anesthesia is applied or not is left at the discretion of the anesthetist and is not specified in this study protocol.

9.3.1.2 Acquisition of Peritoneal Lavage Cytological Specimens

After laparotomy, peritoneal lavage cytological specimens will be taken first for postoperative examination (specific method: draw ascites if they are found; if no ascites are found, 100 ml of physiological saline will be slowly injected into the abdominal cavity; the irrigation sample will be collected at the pouch of Douglas for examination).

9.3.1.3 Intraoperative exploration

Explore the abdominal cavity for any hepatic, peritoneal, mesenteric, or pelvic metastases and gastric serosal invasion

9.3.1.4 Regulations on the extent of the gastrectomy

Follow the Japanese gastric cancer treatment guidelines 2014 (ver. 4) to perform total gastrectomy under the premise of satisfying the oncological principles.

9.3.1.5 Regulations Regarding Greater Omentum Resection

This study protocol requires total greater omentum resection.

9.3.1.6 Regulations Regarding Digestive Tract Reconstruction

The digestive tract reconstruction method is determined by the surgeon according to his/her own experience and the specific intraoperative situation. If instrumental anastomosis is used, the surgeon determines whether manual reinforced stitching of the anastomotic stoma is to be performed; the study protocol does not specify.

9.3.1.7 Regulations Regarding Surgery-related Equipment and Instruments

The energy equipment, vascular ligation method, digestive tract cutting closure, and digestive tract reconstruction instruments are determined by the surgeon responsible for surgery based on experience and actual needs and are not specified in this study protocol.

9.3.1.8 Regulations Regarding Gastric Canal and Peritoneal Drainage Tube

Whether the gastric canal or peritoneal drainage tube is left after surgery is determined by experience and actual needs and is not specified in this study protocol.

9.3.1.9 Regulations Regarding Concurrent Surgical Treatments

If another organ/system disease is present, the responsible surgeon and the relevant department consultants will jointly decide whether a concurrent operation is required and can be performed. The order is determined according to the clinical routine, but these cases will be excluded from the PP set according to the Exclusion Criteria.

9.3.1.10 Regulations Regarding the Processing of Excluded Patients Identified Intraoperatively

If the patient is judged to meet the exclusion requirements during the operation, the study approach will be suspended, and the responsible surgeon will decide upon the subsequent treatment according to the clinical practice of the research center (the therapeutic decision, such as whether to excise the gastric primary focus or metastases, is determined by the responsible surgeon). Data collection and follow-up are still necessary for the excluded subject and should be incorporated into the ITT analysis.

9.3.1.11 Regulations Regarding Photography/Imagery

Use a digital camera (at least 8 megapixels) to take pictures; the photo content required is as follows (see example):

(1) Field of lymph node dissection (6 pictures)

□ Inferior pylorus region (only for patients underwent previous Billorth type I reconstruction, 1 picture)

□ Right-side area of the superior margin of the pancreas (1 picture)

□ Left-side region of the superior margin of the pancreas (1 picture);

□ Right side of the esophagus (1 picture);

□ Left gastroepiploic vessel dividing position (1 picture);

□ Splenic hilus region (1 picture).

(2) After the skin incision is closed (1 picture, measuring scale serving as a reference object).

(3) Postoperative fresh specimens (4 pictures, measuring scale serving as a reference object); 1 picture before and 3 pictures after dissection (mark focus size; 1 picture each of distal and proximal incisional margins). After the specimen is cut open along the greater gastric curvature, a measuring scale is placed as a reference object before taking

pictures to record the following items: the distance between the tumor edge and the proximal incisional margin (1 picture), the distance between the tumor edge and the distal incisional margin (1 picture), and the focus size and appearance of the mucosal face after the specimen is unfolded (1 picture).

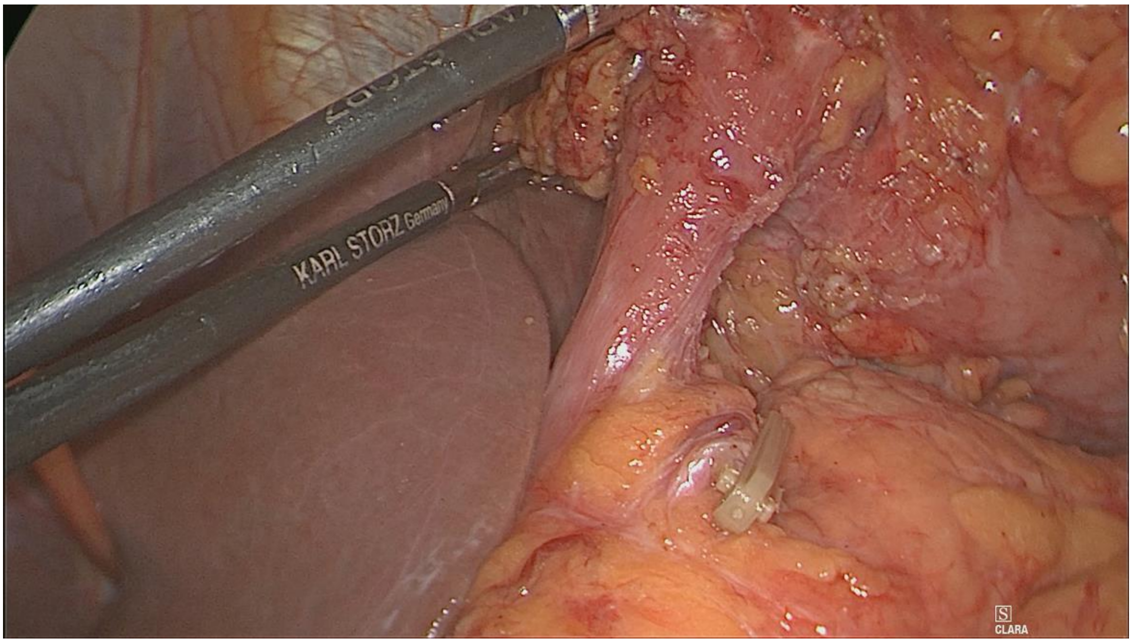

Fig. 2-1 Inferior pylorus area (only for patients underwent previous Billorth type I reconstruction)

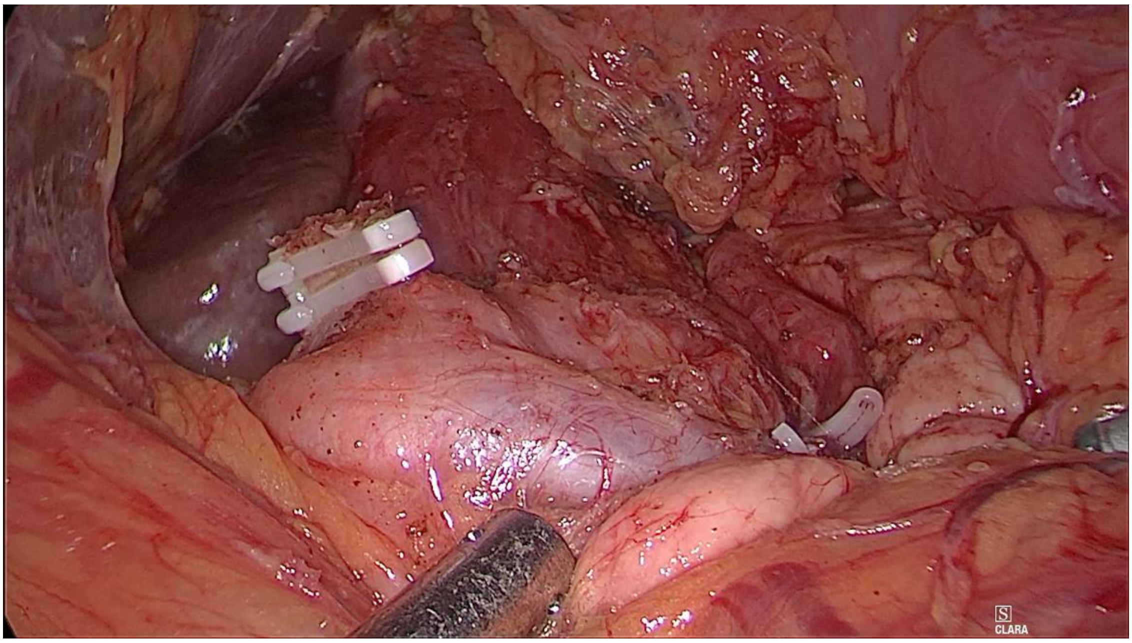

Fig. 2-2 Right-side area of the superior margin of the pancreas

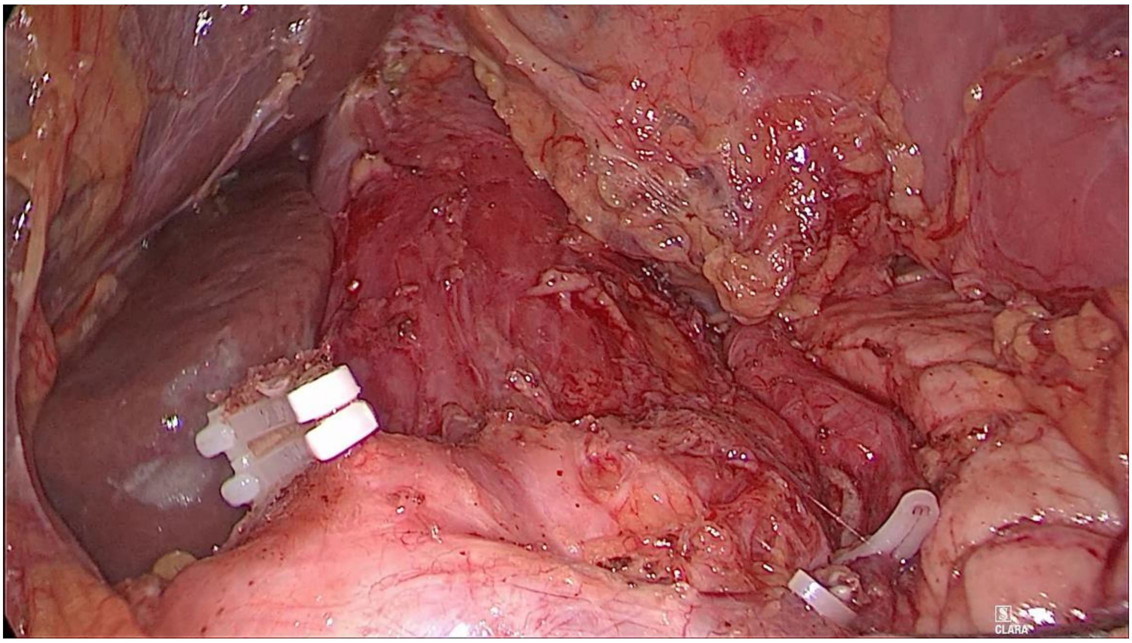

Fig. 2-3 Left-side area of the superior margin of the pancreas

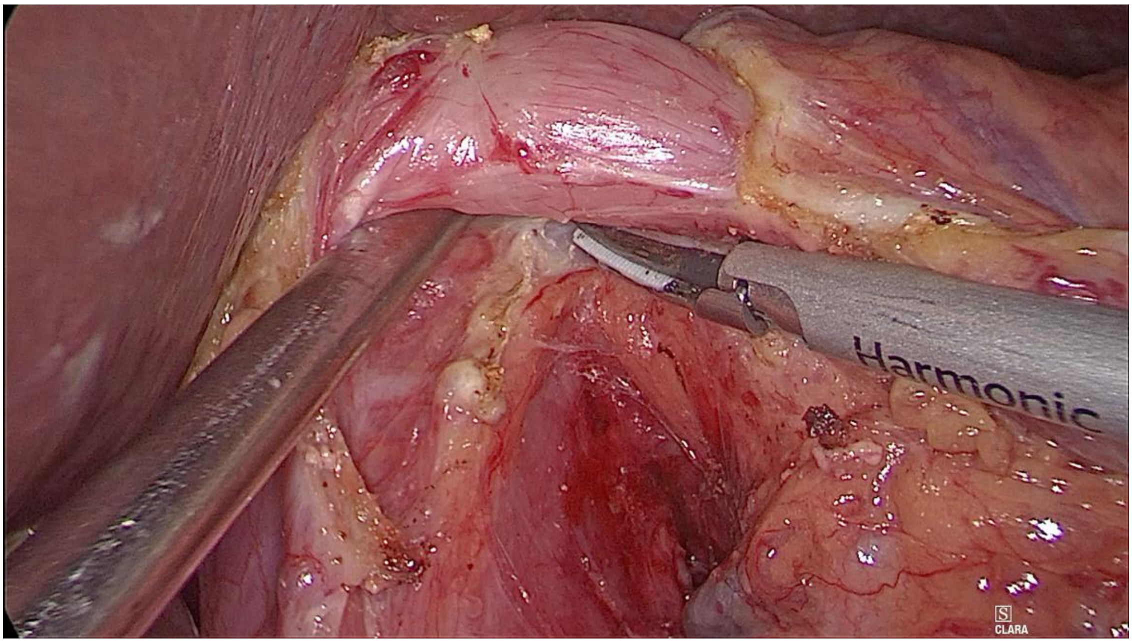

Fig. 2-4 Right side of the esophagus

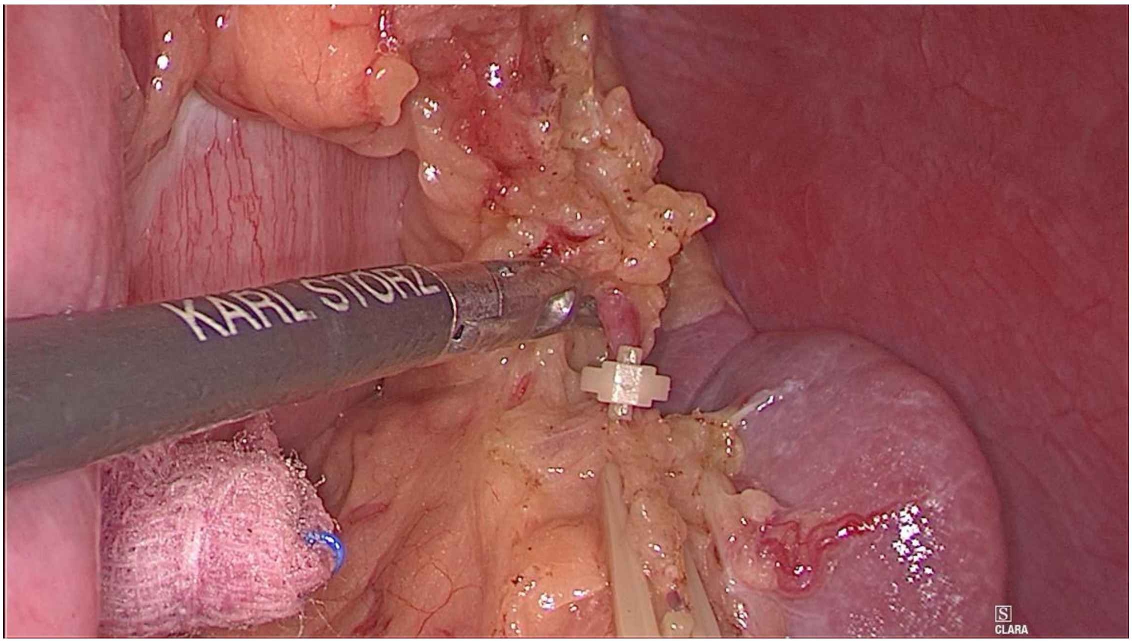

Fig. 2-5 Cut site of the left gastroepiploic vessel

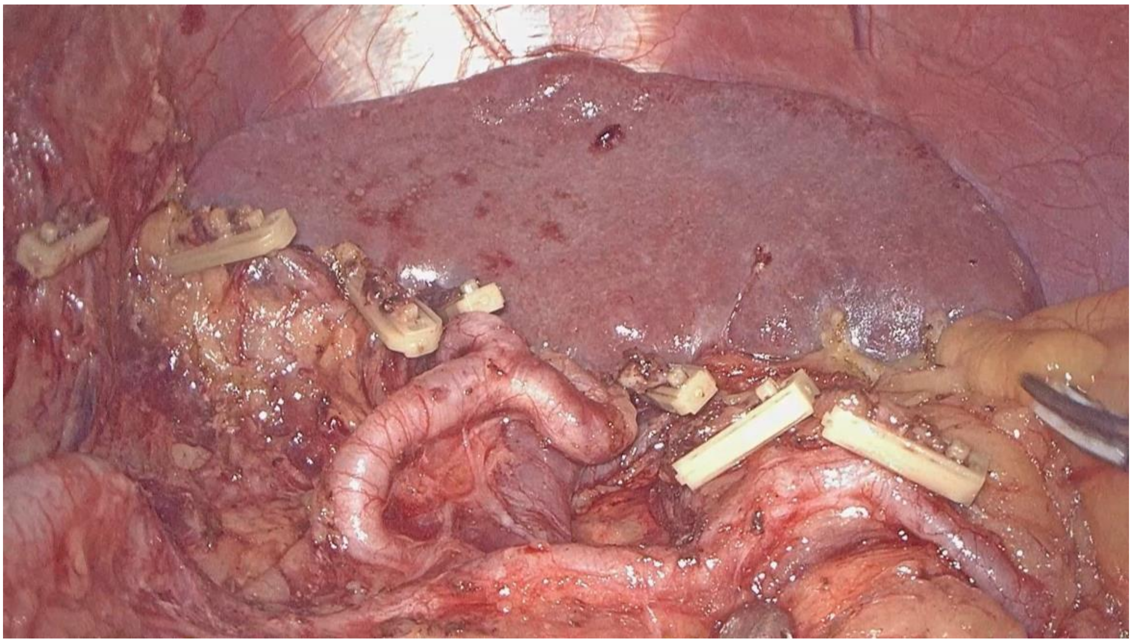

Fig. 2-6 Splenic hilus area (no. 11d and no. 10 lymph nodes)

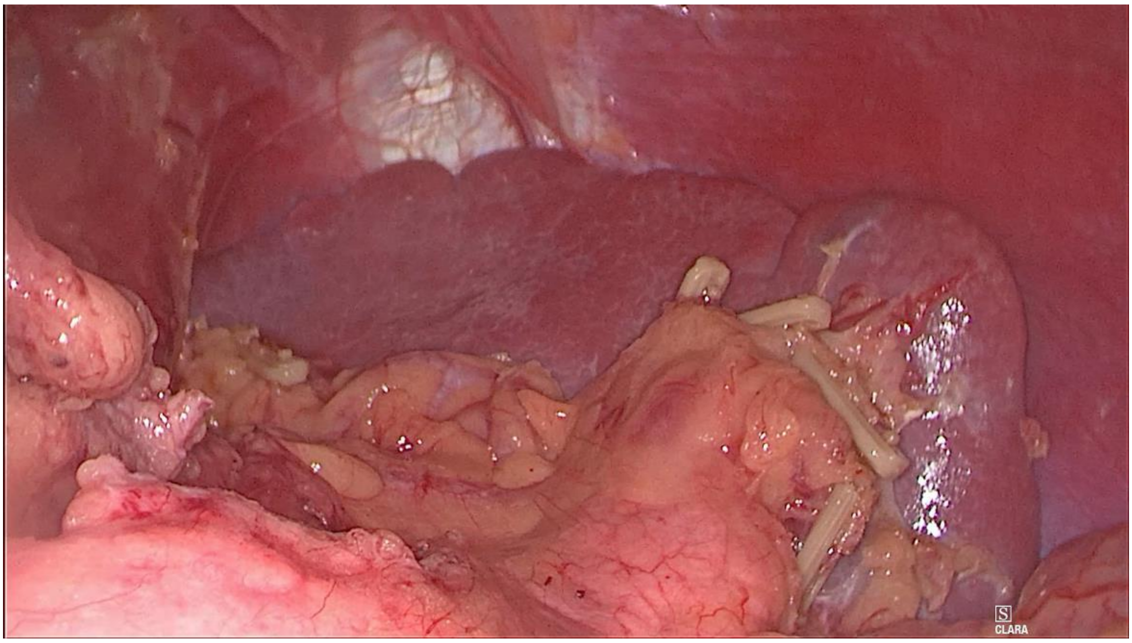

Fig. 2-7 Splenic hilus area (without no. 10 lymph nodes dissection)

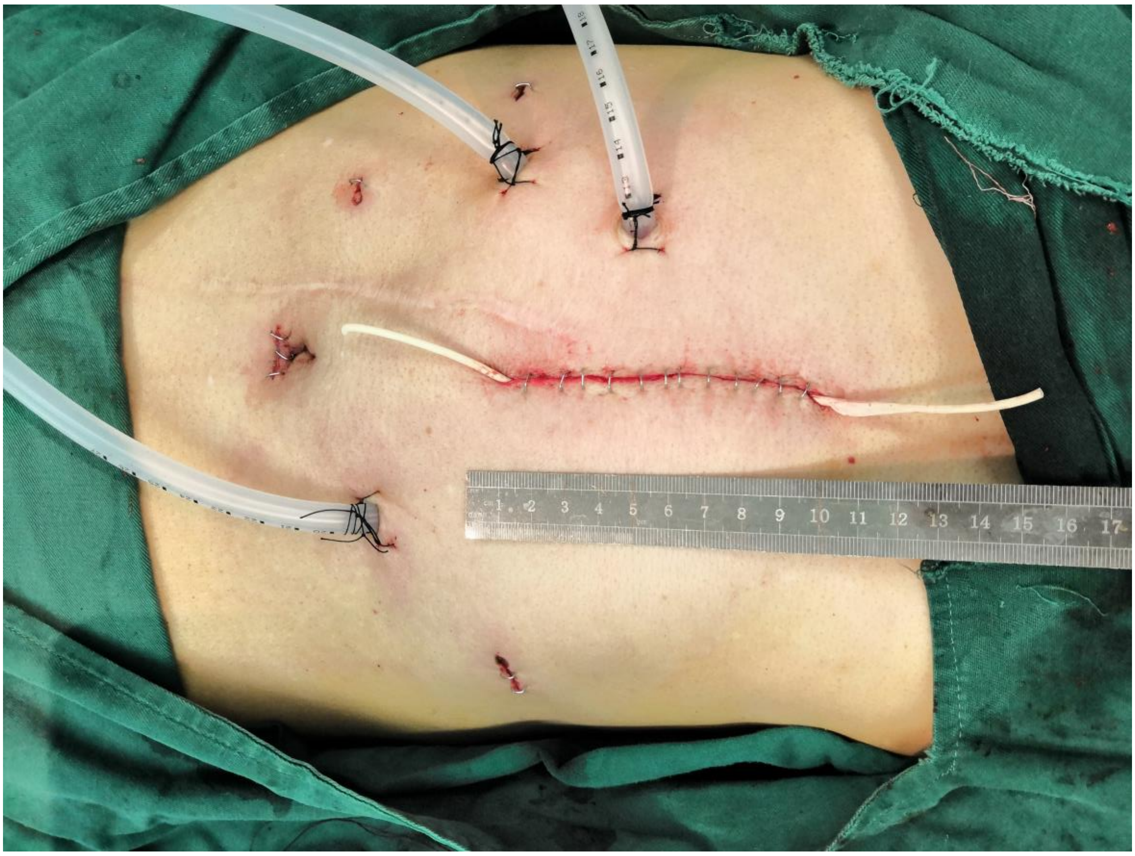

Fig. 2-8 Incision appearance (mark the incision length)

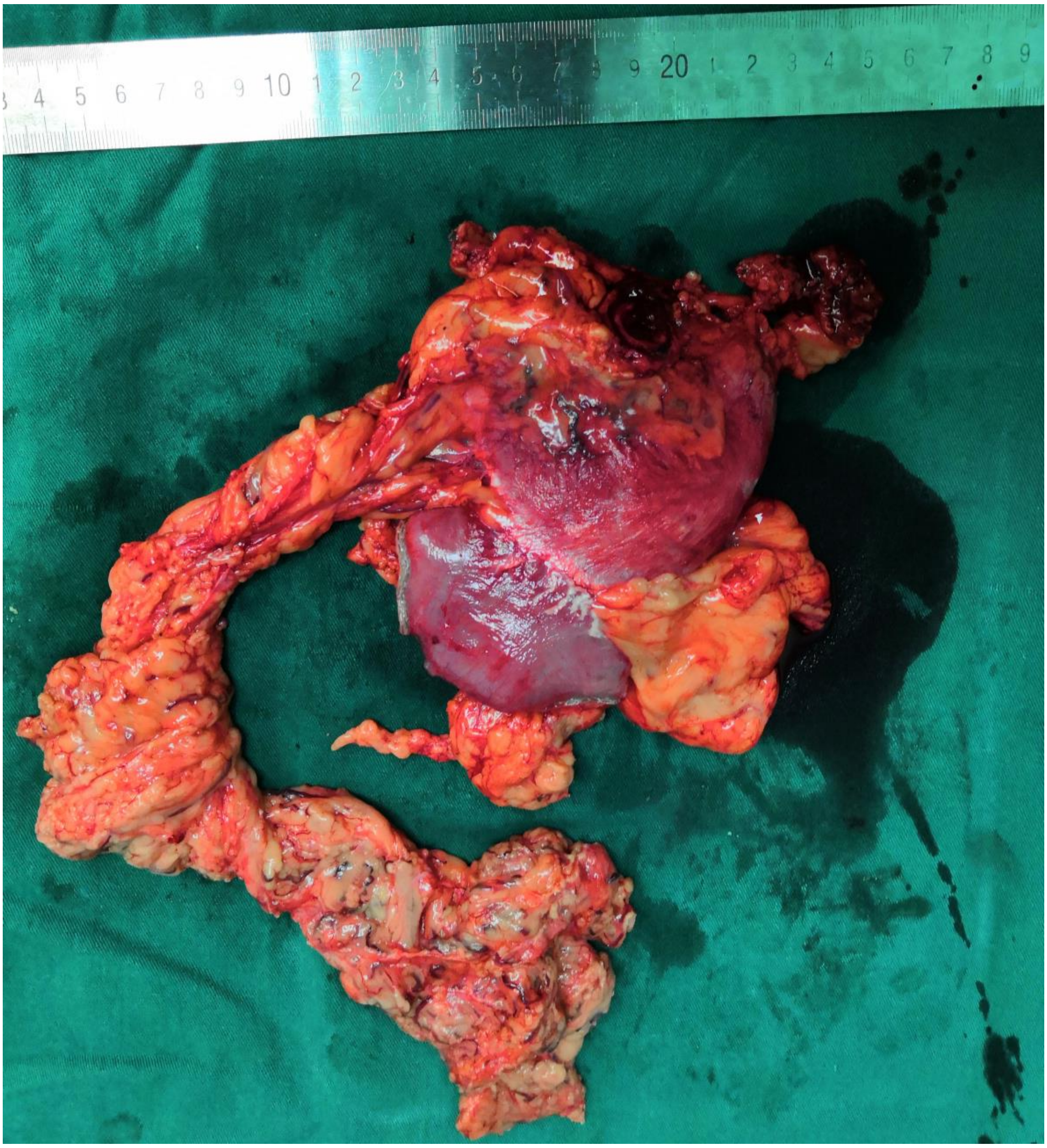

Fig. 2-9 Specimen observation (before dissection)

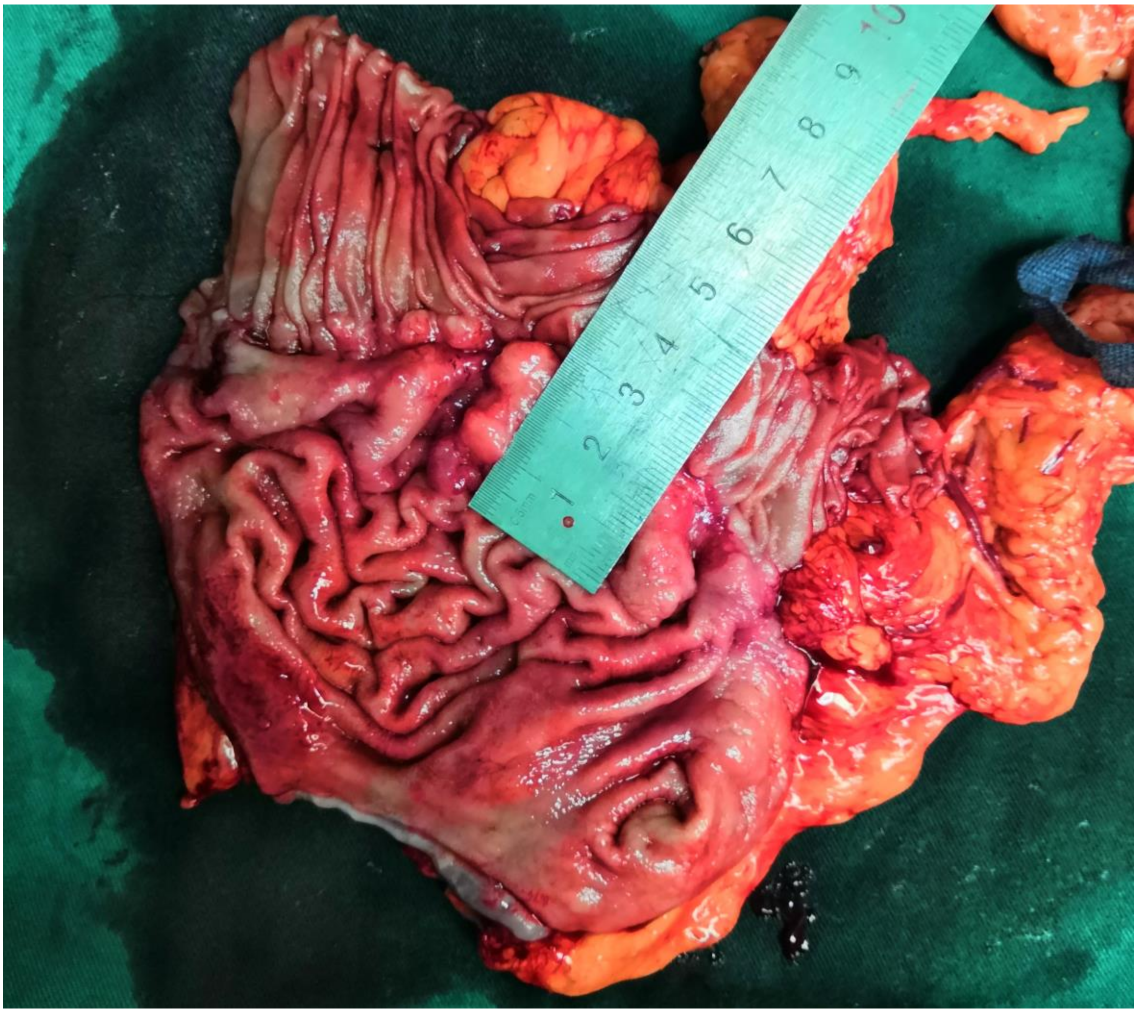

Fig. 2-10 Specimen observation (focus size; the dissection is made along the greater gastric curvature, and the focus and incisional margin on the mucosal face are observed; if the tumor is located at the greater gastric curvature, then the dissection is made along the lesser curvature)

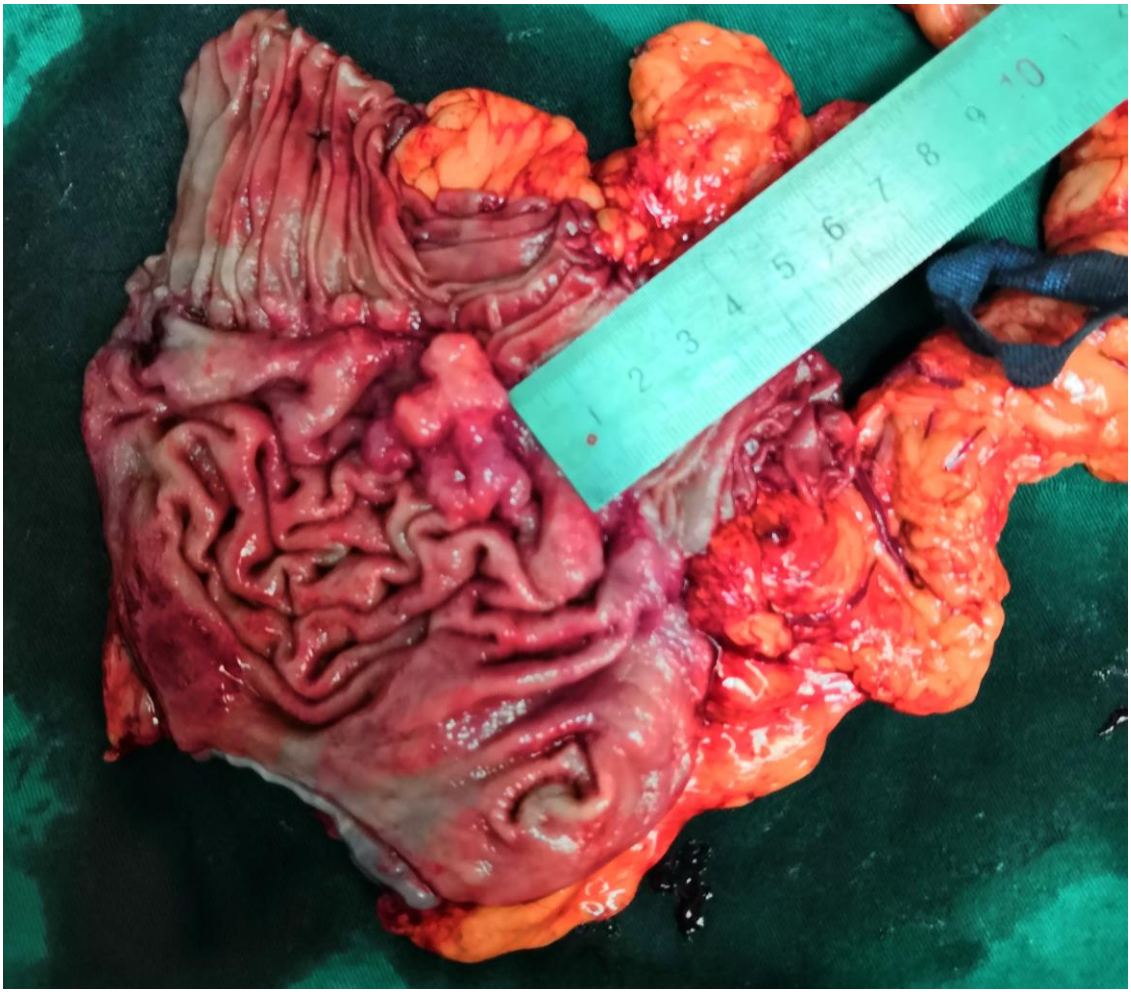

Fig. 2-11 Specimen observation (the distance between the tumor edge and the proximal incisional margin)

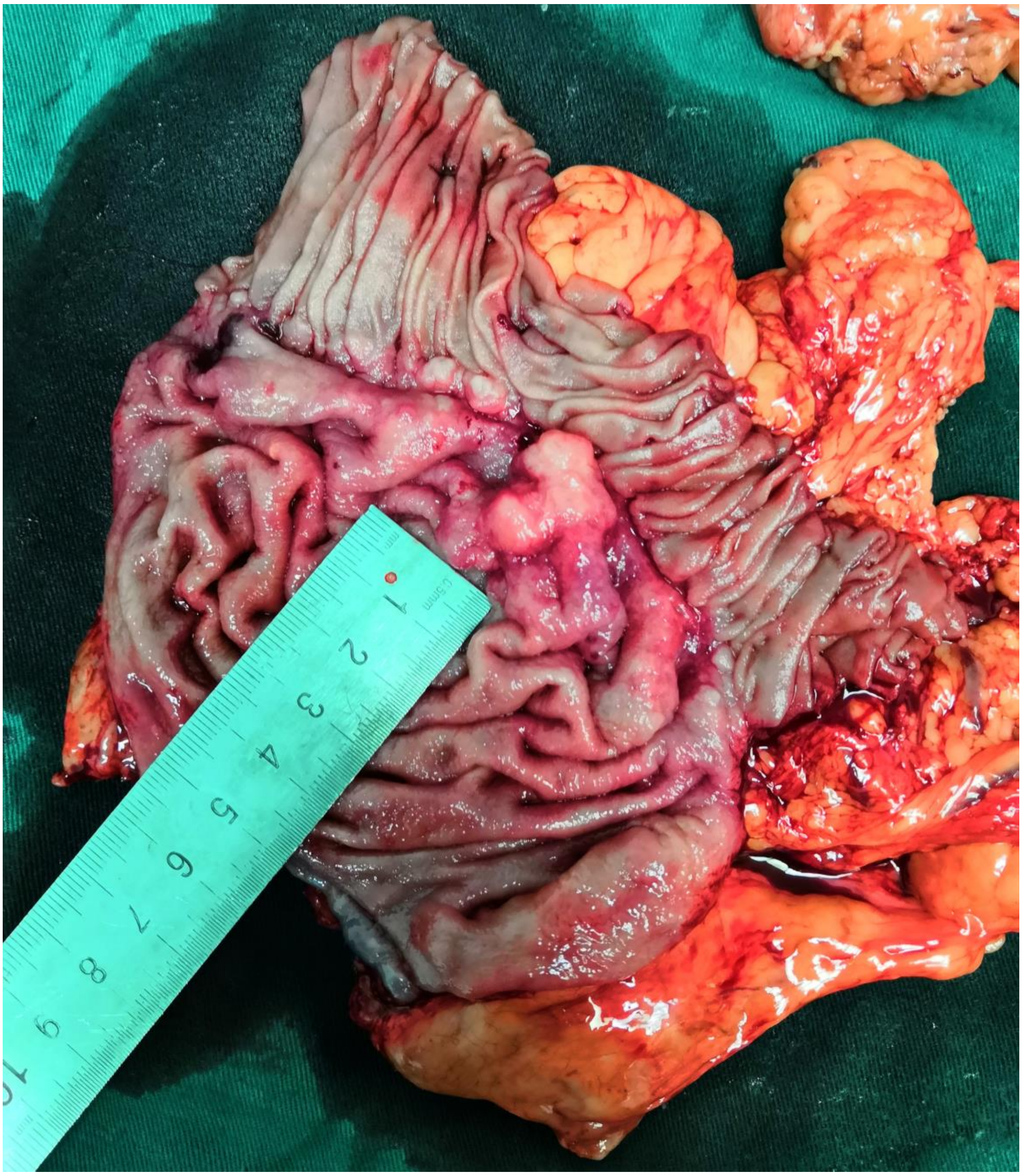

Fig. 2-12 Specimen observation (the distance between the tumor edge and the distal incisional margin)

9.3.1.12 Regulations on the photo/ image privacy protection and naming

No image data shall disclose the personal information of patients.

When the photos/images are viewed or reviewed, the personal information must be processed with mosaics or be covered.

The photographed parts should be marked with unified Chinese name: inferior pylorus area; left gastroepiploic vessel cut site; right-side area of superior margin of the

pancreas; left-side area of superior margin of the pancreas; right side of the esophagus; splenic hilus area; incision appearance; specimen observation (before dissection); specimen observation (focus size); specimen observation (the distance between the tumor edge and the proximal incisional margin); and specimen observation (the distance between the tumor edge and the distal incisional margin).

For example:

Photo Name: [Enrolled number - Inferior pylorus area]

Folder name: [Enrolled number]

9.3.1.13 Criteria for confirming operation quality

To confirm the appropriateness of the surgical procedure, surgery quality, (auxiliary) incision length and specimen integrity will be assessed in the photographs saved (as stated above) The whole laparoscopic surgery procedures will be videotaped, and the unclipped image files will be saved.

9.3.1.14 Saving of imaging data

All photographs and data will be saved in the hard disk or portable digital carrier in digital form, and the surgical video required a specific hard drive to be saved for at least 3 years.

If failure to provide the complete photo according to “Regulations on imagery/photographing” is confirmed, the Research Committee will judge and record the surgery quality as unqualified; however, the case will remain in the PP set data of this study.

9.3.2 Regulations on laparoscopy

9.3.2.1 Regulations on pneumoperitoneum

Carbon dioxide pneumoperitoneum will be used to maintain the pressure at 12-13 mmHg.

9.3.2.2 Regulations on punctures and auxiliary incision

The positions of punctures and auxiliary small incision are not specified; the number of punctures should not exceed 5. There should be only one auxiliary small incision whose length shall not exceed the maximum tumor diameter and necessarily will be less than 10 cm in normal cases. If the auxiliary small incision needs to be longer than 10 cm, the surgeon in charge should make a decision and record the reasons in the CRF.

9.3.2.3 Definition of laparoscopic approach

The operations within the abdominal cavity must be performed with laparoscopic instruments with the support of a camera system. Perigastric disassociation, greater omentum excision, omental bursa excision, lymph node dissection, and blood vessel handling are completed under laparoscopic guidance. For gastrectomy and digestive tract reconstruction use of auxiliary small incisions is allowed and can be completed with an opened abdomen.

9.3.2.4 Regulations on conversion to laparotomy

When intra-abdominal hemorrhage, organ damage and other serious/life-threatening complications which are difficult to control occur during laparoscopic surgery, it is necessary to actively convert to laparotomy. If the anesthesiologist and surgeon consider that intraoperative complications caused by carbon dioxide pneumoperitoneum may threaten the patient’s life, it is necessary to actively convert to open. The surgeon in charge can decide to convert to laparotomy driven by other technical or equipment reasons and will record said reasons. The reasons for the conversion to open must be clearly recorded in the CRF. The incision length of > 10 cm is defined as a case of conversion to open surgery in this study.

9.3.2.5 Subsequent treatment of excluded patients

Whether the patients continue to undergo laparoscopic surgery or converted to open surgery is at surgeon’s discretion according to clinical experience.

9.3.3 Operative parameters (same for both groups)

Completed by the research assistant on the day of the operation. specific projects include:

(1) Name of responsible surgeons

(2) Operation time (min)

(3) Type of operation, digestive tract reconstruction, intraoperative damage and whether the tumor was ruptured during surgery (intact rupture of the capsule)

(4) Length of incision (cm)

(5) Conversion to open surgery or not and the reasons for this decision

(6) Intraoperative estimated blood loss (ml; from skin cutting to stitching, intraoperative blood loss = (postoperative gauze weight, grams - preoperative gauze weight, grams) *1ml/g+ suction fluid, ml)

(7) Blood transfusion (ml): in this study, the blood transfusion event is defined as transfusion of red cell suspension (ml) or whole blood (ml)

(8) Tumor location

(9) Tumor size (maximum tumor diameter, mm)
(10) Distant metastasis (location)

(11) Proximal resected margin (mm), distal resected margin (mm), radicality (R0/R1/R2)

(12) Intraoperative complications (occurring from skin incision to skin closure) including:

surgery-related complications: intraoperative hemorrhage and injury: A. Vascular injury: A vascular injury is defined as a blood vessel with either a blood vessel clamp or a titanium clamp closure and an intra-cavity suture or any other method to control the bleeding. B. Organ damage: maybe including diaphragmatic injury, esophageal injury, duodenal injury, colon injury, small intestine injury, spleen injury (excluding <1/3 spleen ischemia), liver injury, pancreatic injury, gallbladder injury, kidney damage etc.

C. Tumor rupture: tumor envelope integrity damage

Pneumoperitoneum-related complications: high-blood carbonate, mediastinal emphysema, subcutaneous emphysema, air embolism, respiratory circulation instability caused by abdominal pressure.

Anesthesia-related complications: Allergic reactions.

(13) Intraoperative death (occurring during the time period from skin cutting to skin stitching completion) regardless of reason.

9.4 Postoperative management

9.4.1 The use of prophylactic analgesics

Continuous postoperative prophylactic intravenous analgesia is allowable but not mandatory within postoperative 48 hours; its dose, type and rate of infusion should be determined by the anesthesiologist according to clinical practices and specific patient conditions. The repeated use of prophylactic analgesics is not allowed beyond 48 hours after the end of surgery, unless it is judged necessary.

9.4.2 Fluid replacement and nutritional support

Postoperative fluid infusion (including glucose, insulin, electrolytes, vitamins, etc.) or nutritional support (enteral/parenteral) will be performed based on doctor's experience and routine clinical practices and is not specified in this study. After oral feeding, it is allowable to stop or gradually reduce fluid infusion/nutritional support.

9.4.3 Post-operative rehabilitation management

Management methods of incision, stomach and abdominal drainage tube: Follow regular diagnosis and treatment approaches. Eating recovery time, diet transition strategies: Follow regular diagnosis and treatment approaches.

9.4.4 Discharge standard

Patients needed to meet the following criteria for discharge: 1) satisfactory intake of a soft diet. 2) move around of their bed. and 3) absence of complications by routine clinical examinations. This information will be recorded in the CRF.

9.4.5 Postoperative observation items

Definition of “postoperative day n”: One day from 0:00 to up to 24:00. Up to 24:00 on the day of surgery is “postoperative day 0;” the next day from 0:00 to up to 24:00 is “postoperative day 1;” and so on. From the first postoperative day until hospital discharge, the research assistant should timely fill in the following items and specific observation items including:

(1) Pathologic results：

Original lesion tissue typing, Distant metastasis, and parts, NIH Hazard grading, Radical surgery degree (R0/R1/R2)

(2) Postoperative complications：

Postoperative complications are divided into and short-term complications after surgery and long-term complications after surgery. Short-term is defined as within 30 days of surgery or the first discharge if the hospital days > 30 days. Long-term is defined as the period from 30 days or more after the operation, or the first discharge (the hospital days after surgery >30 days) to 3 years after the operation.

Classification and name of complication	Diagnostic criteria
Abdominal bleeding	Intra-abdominal hemorrhage requires blood transfusion, emergency endoscopy or surgical intervention to eliminate anastomotic bleeding
Anastomotic bleeding	The postoperative gastrointestinal decompression tube continued to have fresh red blood outflow; the hemoglobin drops more than 1g/dL
Gastrointestinal anastomotic stoma Fistula	Using gastrointestinal angiography to see contrast agent leak out from the anastomosis, or the blue drainage outflow through tube after oral Methylene blue to eliminate the possibility duodenal stump fistula and intestinal fistula
Duodenal Stump Fistula	Using gastrointestinal angiography to see contrast agent leak out from

	the duodenal stump to eliminate the anastomotic fistula or intestinal fistula
Intestinal fistula	Using gastrointestinal angiography to see the blue drainage outflow through tube after oral Methylene blue to eliminate anastomotic fistula and duodenal stump fistula
Stenosis of Anastomosis	Endoscopic examination with a 9.2-mm endoscopy not passing through the anastomosis to eliminate recurrence of tumors
Input jejunal loop obstruction	Abdominal pain, abdominal distension, vomiting and other symptoms. Abdominal flat to see the right upper abdomen expansion of the intestinal loop, and there is a liquid plane, or a visible input loop jejunum giant expansion by barium meal examination.
Intestinal obstruction after operation	Abdominal X-ray shows a plurality of liquid planes and the phenomenon of intestinal effusion with visible isolated, fixed, swelling of the intestinal loop. Total Abdominal CT showed edema, thickening, adhesion of intestinal wall, accumulation of gas in intestinal cavity, uniform expansion of bowel and intra-abdominal exudation.
Early dumping syndrome	Combined the symptoms of sweating, heat, weakness, dizziness, palpitations, heart swelling feeling, vomiting, abdominal colic or diarrhea with the signs of tachycardia, blood pressure micro-rise, breathing a little faster sign after meal 15-30 minutes, and solid phase radionuclide gastric emptying scanning tips stomach quickly emptying.
Late dumping syndrome	Feeling hungry, flustered, out of sweating 2-3 hours after the meal . Blood sugar is less than 2.9mmol/L, excluding other diseases that cause hypoglycemia
Intestinal ischemia and necrosis	Under the digestive endoscopy, the intestinal mucosa congestion, edema, bruising, mucosal hemorrhage, the mucous membrane being dark red, the vascular network disappearing, can have part mucosal necrosis, following with mucosal shedding, ulcer formation with annular, longitudinal, snake and scattered in the ulcer erosion.
Internal hernia	Postoperative CT findings of cystic or cystic and solid mass, and intestinal aggregation, stretching, translocation, abnormal mesenteric movement, and thickening of the blood vessel.
Alkaline reflux esophagitis	1. Endoscopic examination and biopsy of the upper gastrointestinal tract showed evidence of inflammation of the mucous membranes and gastrointestinal metaplasia; 2. CT scan and gastrointestinal barium meal examination showed no expansion or obstruction of the input

	loop.
Incision splitting	Including partial dehiscence of the incision and full-layer dehiscence
Incisional hernia of abdominal wall	The swelling tumor showing in the surgical scar area or abdominal wall swelling when standing or force. CT shows ventral wall continuity interruption and hernia content extravasation
Incision infection	Thickening of the soft tissue at the incision, in or below the incision of gas, exudation, swelling of the incision or pus from the incision extrusion, or secretion culture of pathogenic bacteria.
Lymphatic leakage	A chyle test when abdominal drainage fluid exceeded 300 ml/day for 5 consecutive days after postoperative day 3.
Pneumonia	Complies with one of the following two diagnostic Criteria: 1. Auscultation/percussion voiced + one of the following: fresh sputum or sputum character changes; blood culture (+); bronchoalveolar lavage fluid, anti-pollution sample brush, biopsy specimens cultured pathogenic bacteria. 2. Chest film hints of new or progressive infiltration + one of the following: fresh sputum or sputum character changes, blood culture (+), bronchoalveolar lavage fluid, anti-pollution sample brush, biopsy specimens cultured pathogenic bacteria; isolate virus or detect IgM, IgG (+) of respiratory viral
Acute pancreatitis	Irritability, abdominal pain, anti-jumping pain, fever, leukocyte increase and blood amylase increased occuring and diagnosed by ultrasound or CT within 3 days after surgery.
Acute cholecystitis	Serum bilirubin exceeding 85μmol/l and ultrsound examination shows gallbladder enlargement, wall thickness, signal and sound shadow of gallbladder stone, bile internal sediment, gallbladder contraction bad etc.
Pleural effusion/infection	CT scan showed the localized fluid low density area of thoracic cavity, which could accompany with gas, and culture pathogenic bacteria in thoracic endocrine.
Abdominal infection	There is at least one of the following evidences in abdominal cavity within 30 days after operation: 1. discharge of pus, with/without microbiological examination; 2. bacterial culture positive; 3. diagnosed by detection, pathology, imaging findings.
Pelvic infection	Symptoms of systemic infection or rectal irritation, combined with a rectal finger examination and touching tenderness, or a married woman with a posterior vault to extract pus-based fluid

Sepsis	The following two conditions are available: 1. There is evidence of active bacterial infection, but the blood culture does not necessarily appear pathogenic bacteria; 2. meeting two of the following four items at the same time: (1). body temperature >39. 0℃ or ＜35.5 ℃ for 3 consecutive days, (2). heart rate > 120 times/min; (3). total white blood cells >12. 0*10^9/L or <4.0*10^9/l, wherein neutrophils >0. 80, or naïve granular cells >0. 10; (4).Respiratory frequency > 28 times/min
Urinary system infection	Symptoms of urine frequency, urgency and urine pain etc. and urine bacteria culture colony count 1000~10 million/ml in the absence of antibiotics; No symptoms of urine frequency, urgency and urine pain etc, urine bacterial culture colony count ≥ 100,000/ml
Pancreatic fistula	The level of amylase in the drainage fluid is three times than normal level.
Bile fistula	Symptoms of abdominal distension, Abdominal pain, tenderness, anti-jumping pain, muscle tension, abdominal puncture or drainage fluid for bile
Celiac fistula	The drainage fluid is milky white, and more than 200ml/d and and does not decrease for 48 hour, the celiac qualitative test is positive, and the level of triglyceride >110 mg/dL at the same time.
Nutritional disorder after gastrectomy	In the presence of weight loss, anemia, malnutrition bone disease, vitamin A deficiency and other symptoms, laboratory tests suggest that the intestinal absorption function test is abnormal, excluding other causes of nutritional disorders
Bone disease after gastrectomy	Lumbar back pain, length shortening, kyphosis, bone fractures and other symptoms. Bone density decreased combining with elevated alkaline phosphatase and serum calcium reduction, the concentration of serum 25-(O1) D3 and 1,25-(O1) 2D3 increasing and the serum parathyroid hormone increasing. Exclusion of bone disease caused by other causes.
Subcutaneous emphysema	visible the irregular speckle shadow under the skin in the horizontal flat sheet.
Mediastinal emphysema	In the posterior and anterior flat fame, a long narrow gas shadow rises to the neck soft tissue along the mediastinal side, forming a thin-line dense shadow. In the lateral flat there was a visible and clear band between the heart and the sternum. The CT examination, if necessary, shows gas density line-like shadow around the mediastinal and

	mediastinal pleura closing to the direction of the lung field.
Postoperative hemorrhage	An amount of hemorrhage exceeding 300 ml.
Postoperative cardiac dysfunction	The symptom of snus tachycardia, sinus bradycardia, supraventricular tachycardia, ventricular tachycardia, and other arrhythmias, or heart failure preoperatively none-existing and postoperatively appearing,.and other causes of the above-mentioned manifestations are excluded.
Hepatic dysfunction	Bilirubin increasing and the levels of AST and ALT >5 times after operation and these symptoms no existing before sugery,
Kidney function failure	Postoperative continuing renal function insufficiency, blood creatinine rising 2mg/dl, or acute renal failure needing dialysis treatment.
Cerebral embolism	Acute onset, hemiplegia, aphasia and other focal neurological function deficits. Embolism site has low-density infarction, of which border is not clear and no obstructional performance within 24-48 hours after the onset.
Pulmonary embolism	Characteristics of dyspnea, chest pain, syncope, shortness of breath, right ventricular insufficiency and hypotension, pulmonary angiography revealed a filling defect.
Venous thrombosis of lower extremities	Local tenderness, swelling, purple skin color, combined with intravenous angiography to show the filling defect
Mesenteric arterial embolization	Patients with acute abdominal pain, vomiting, diarrhea, abdominal x-ray of intestinal tract filling with gas or existing liquid level, abdominal angiography revealed a filling defect.
DIC	1.There are basic diseases easily leading to DIC, 2. There are more than two clinical performances: (1) severe or multiple bleeding tendencies; (2) Microcirculation disorder or shock cannot be explained by the original disease. (3) Extensive skin mucosal embolism, focal ischemic necrosis, shedding and ulcer formation, or unexplained lung, kidney, brain and another organ failure. (4) anticoagulant treatment.is effective. 3. The laboratory meets the following conditions: (1) there are 3 or more experimental abnormalities: platelet count, prothrombin time, activated partial coagulation enzyme time, thrombin time, fibrinogen level, D-two poly, and (2) difficult or special cases for special examination.
Other	Complications other than the above complications, which do not exist before surgery but appear after surgery

Severity of complication is graded according to Clavien–dindo complication scoring system, [19]

IIIA level and above are serious complication

I: Any deviation from the normal postoperative course without the need for pharmacologic treatment or surgical, endoscopic, and radiologic interventions. Allowed therapeutic regimens are drugs as antiemetics, antipyretics, analgesics, and diuretics, and electrolytes and physiotherapy. This grade also includes wound infections opened at the bedside.

II：Requiring pharmacologic treatment with drugs other than such allowed for grade I complications. Blood transfusions and total parenteral nutrition are also included.

III：Requiring surgical, endoscopic, or radiologic intervention

IIIa：Intervention not under general anesthesia

IIIb：Intervention under general anesthesia

IV：Life-threatening complication (including CNS complications) requiring IC(intermediate care)/ICU(intensive care unit) management

IVa：Single organ dysfunction (including dialysis)

IVb：Multiple organ dysfunction

V：Death as a result of complications

(3) Blood test items (At postoperative day 1, 3, 5)

Peripheral blood routine assessment: Hb, RBC, WBC, LYM, NEU, NEU%, PLT, and MONO;

 Blood biochemistry: Albumin, prealbumin, total bilirubin, AST, ALT, creatinine, urea nitrogen, fasting blood glucose, potassium, sodium, chlorine, calcium, and CRP.

(4) Postoperative rehabilitation evaluation：

Time to first ambulation (hours), time to first flatus (hour), time to liquid diet, time to semi-liquid diet (hour), daily body temperature maximum from surgery to out-patient (℃), time to removal of gastric tube (d), daily volume of gastric drainage (ml), time to removal of abdominal drainage tube (d), daily volume of drainage (ml).

Blood transfusion volume (ml) from the end of surgery to postoperative discharge: a transfusion event is defined as infusion of the red blood cell suspension (ml) or whole blood (ml)

Postoperative hospital stays (days): The period from surgery day to first discharge day

9.5 Follow-Up

9.5.1 Follow-up Period and strategy

Follow-up visits will be completed by special persons for all cases selected in this study. All patients are followed up every 3 months during the first 2 years and then every 6 months beyond the third year (1, 3, 6, 9, 12, 15, 18, 21, 24, 30, and 36 months after the operation). This study suggests that the above examinations should be conducted in the same hospital, but does not exclude outer court review. For outer court review, it is recommended to visit a tertiary teaching hospital, and these information will be recorded by the follow-up specialist. The tumor recurrence or metastasis and the survival status of all patients are evaluated and recorded according to the results of the various examinations. Patients who refuse to follow the protocol should be recorded as lost to follow-up, and at the end of the study, these cases should be analyzed together with cases lost to follow-up in line with the criteria of this study.

9.5.2 Assessment items during the follow-up

(1) Systematic physical examination:

The doctor in charge will regularly conduct a systematic physical examination at the time of each follow-up, giving particular attention to superficial lymph nodes, abdomen, and signs of metastases, and so on.

(2) Blood test items:

 Peripheral blood routine assessment: Hb, RBC, WBC, LYM, NEU, NEU%, PLT, MONO

 Biochemistry: Albumin, pre-albumin, total bilirubin, Indirect bilirubin, direct bilirubin, AST, ALT, creatinine, urea nitrogen, Total cholesterol, triglycerides, fasting blood glucose, potassium, sodium, chlorine, calcium, serum tumor markers: CEA, CA19-9, CA72-4, CA12-5, AFP.

(3) Imaging items:

 Abdomen CT scan (including cavity) (thickness of 10 mm or less, in case of contrast agent allergy, CT horizontal scanning is only allowable or conversion to MRI). Upper gastrointestinal endoscopy (histopathological biopsy, endoscopic ultrasonography when necessary). Chest X-ray (AP and lateral views): lung field condition. Other means of evaluation: gastrointestinal radiography, ultrasonography of other organs, whole body bone scanning, and PET-CT, and so on.

9.5.3 Follow-up process

Postoperative	3 months	6 months	9 months	12 months	15 months	18 months	21 months	2 years	2 years and a half	3 years
Date of actual visit										
Physical examination										
Blood routine test										
Blood biochemistry										
Tumor markers										
Chest X-ray										
Upper digestive tract endoscopy										
Abdominal CT										
Abdominal ultrasound										
Others (if necessary)										

9.6 Postoperative adjuvant therapy

9.6.1 Indications for postoperative adjuvant chemotherapy

After completion of the surgical treatment, according to the pathological results, patients with T2 and above or N_+ are administered postoperative adjuvant chemotherapy according to the provisions of this program.

9.6.2 Postoperative adjuvant chemotherapy

Chemotherapy regimen is based on 5-FU (5-fluorouracil). The adjuvant chemotherapy cycle is half a year (6 months after surgery).

In cases of good physical and tolerable conditions, chemotherapy is first started within 8 weeks after surgery and then according to the regularity of the chemotherapy cycle.

During the chemotherapy period, follow-up plan should be executed as regular.

When tumor recurrence occurs during chemotherapy, the adjuvant chemotherapy

regimen of this study is discontinued. The subsequent treatment is decided by the research center according to the clinical treatment guidelines. This study does not make regulations, but the cause and subsequent treatment plan should be recorded in the CRF.

□□If there is no recurrence during chemotherapy, adjuvant chemotherapy is terminated after the completion of chemotherapy, and the follow-up plan continues.

□□Adjuvant chemotherapy requires written approval from the patient.

□□Subjects that refuse postoperative adjuvant chemotherapy or do not complete the adjuvant chemotherapy are not excluded from this study, but the cause is marked and recorded in the CRF.

□□For elderly patients (80 years and older), considering differences in the physical fitness of the elderly and ensuring the safety of patients, the research center decides according to the clinical experience. This study does not recommend or stipulate any chemotherapy regimen for patients of this age.

□□Patients who choose adjuvant chemotherapy, irregular chemotherapy, or a non-first-line regimen are not excluded from the study, but the FUGES-004 Efficacy and Safety Evaluation Committee is obliged to monitor patient safety during follow-up. The patients' chemotherapy medication must be recorded in the CRF.

□□The principles of processing in terms of the method of administration of adjuvant chemotherapy, toxic reactions, and dose adjustment with intolerance are implemented according to the original literature on drug toxicity and dose adjustment for each chemotherapy regimen. This study does not regulate these principles.

9.6.3 Safety Evaluation Indicators of Postoperative Adjuvant Chemotherapy

The safety evaluation indicators for patients enrolled in the study should be immediately filled out by the investigators before and after each postoperative adjuvant chemotherapy cycle, with specific items including the following:

(1) Performance Status (ECOG)

(2) Subjective and objective status (according to the records of CTCAE v3.0 Short Name)

(3) Blood tests:

□□Peripheral venous blood assessment: Hb, RBC, WBC, LYM, NEU, NEU%, PLT,

MONO.

□□Blood biochemistry: albumin, prealbumin, total bilirubin, AST, ALT, creatinine, urea nitrogen, fasting blood glucose, serum tumor markers (CEA, CA19-9, CA72-4, CA12-5, AFP)

(4) Safety evaluation items to be implemented during chemotherapy when necessary (refer to CTCAE v3.0):

□□Neurotoxicity

□□Cardiovascular system (cardiac toxicity, ischemic heart disease, etc.)

□□Bone marrow suppression and infections due to immune dysfunction

□□Others

9.7 Study calendar

Observation Stage	Performance Status	Blood biochemistry	Tumor markers	Electrocardiogram, respiratory function	Upper gastrointestinal endoscopy	Chest X-ray, full abdominal CT Or ultrasound	Eligbility confirmation notice	Preoperative, postoperative complications	Adverse chemotherapy events	CRF- Preoperative	CRF-Intraoperative	CRF- Postoperative	CRF- treatment end report	CRF- follow-up observation surgery
Selection Application	○	○	○	○	○	○								
After selection and prior to surgery							○			○				
Intraoperative period								○			○			
Early postoperative period								○				○	○	
Before postoperative first chemotherapy	○	○	○			○								
Regular chemotherapy	○	○	○						○					

Follow-up period Postoperative advanced stage	At postoperative 1 month (±7 days)	○	○	○			○		○						○
	At postoperative 3 months (±15 days)	○	○	○					○						○
	At postoperative 6 months (±15 days)	○	○	○			○		○						○
	At postoperative 9 months (±15 days)	○	○	○					○						○
	At postoperative 1 year (±15 days)	○	○	○			○		○						○
	At postoperative 15 months (±15 days)	○	○	○					○						○
	At postoperative 18 months (±15 days)	○	○	○			○		○						○
	At postoperative 21 months (±15 days)	○	○	○					○						○
	At postoperative 2 years (±15 days)	○	○	○				○	○						○
	At postoperative 2 years (±15 days)	○	○	○				○	○						○
	At postoperative 3 years (±15 days)	○	○	○				○	○						○

○: must do

9.8 Definitions involved in SOP

9.8.1 ECOG performance status score

According to the simplified performance status score scale developed by the ECOG, the patients' performance status can be classified into 6 levels, namely 0-5, as follows:

0: Fully active, able to carry on all pre-disease performance without restriction

1: Restricted in physically strenuous activity but ambulatory and able to carry out work of a light or sedentary nature, e.g., light housework, office work

2: Ambulatory and capable of all self-care but unable to carry out any work activities. Up and about more than 50% of waking hours

3: Capable of only limited self-care, confined to bed or chair more than 50% of waking hours

4: Completely disabled. Cannot carry on any self-care. In total, confined to bed or chair

5: Dead

Patients at levels 3, 4 and 5 are generally considered to be unsuitable for surgical treatment or chemotherapy.

9.8.2 ASA classification

According to the patients' physical status and surgical risk before anesthesia, the American Society of Anesthesiologists (ASA) has categorized patients into 6 levels (I-VI levels): □□

Class I: A normal healthy patient.

Class II: A patient with mild systemic disease.

Class III: A patient with a severe systemic disease that is not life-threatening.

Class IV: A patient with a severe systemic disease that is a constant threat to life.

Class V: A moribund patient who is not expected to survive without the operation. The patient is not expected to survive beyond the next 24 hours without surgery.

Class VI: A brain-dead patient whose organs are being removed with the intention of transplanting them into another patient.

Generally, Class I/II patients are considered good for anesthesia and surgical tolerance, with a smooth anesthesia process. Class III patients are exposed to some anesthesia risks;

therefore, good preparations should be fully made before anesthesia, and effective measures should be taken to prevent potential complications during anesthesia. Class IV patients are exposed to the most risks, even if good preoperative preparations are made, and have a very high perioperative mortality rate. Class V patients are moribund patients and should not undergo an elective surgery.

9.8.3 Oncology-related definitions

In this study, tumor staging is based on 8th AJCC staging system; surgical treatment follows the Japanese Gastric Cancer Treatment Guidelines, Physicians Edition, 4rd Edition, 2014.05, and other writing and recording principles follow the Japanese Gastric Cancer Statute 15th.

9.8.3.1 Primary focus location

The greater and lesser curvature of the stomach are divided into three equal parts, the U (upper), M (middle) and L (lower) areas, connected to the corresponding points. Esophagus and duodenum infiltration are recorded as E (esophagus), and D (duodenum), respectively. If the lesions are located in two or more adjacent areas, they should be recorded in the order of the main portions of the lesions.

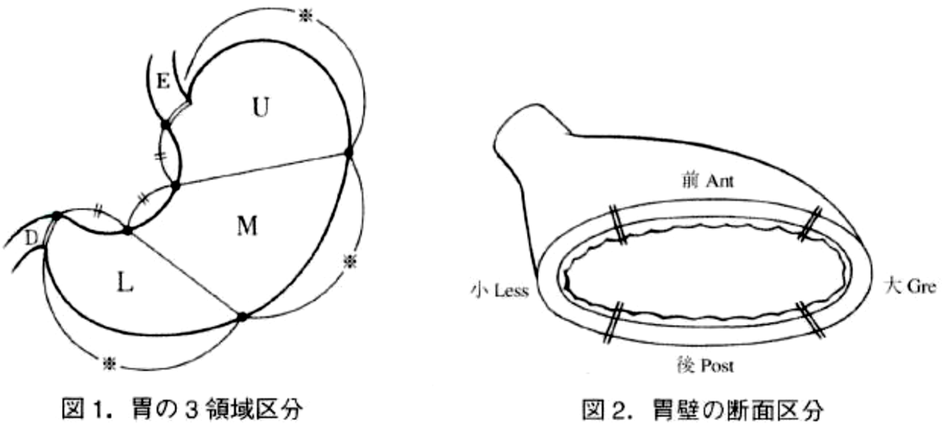

Fig. 3. Division of the Three Areas of the Stomach

9.8.3.2 Tumor staging record

9.8.3.2.1 Recording principle

The two staging records for clinical classification and pathological classification involve T (invasion depth), N (regional lymph node) and M (distant metastasis), which are expressed in Arabic numerals and denoted as x if indefinite.

Clinical classification	Pathological classification
Physical examination X-ray, endoscopy, diagnostic imaging<br>laparoscopy, intraoperative observations (laparotomy/laparoscopy), biopsy, cytology, biochemistry, biology examination	Pathological diagnosis of the endoscopic/surgical specimens<br>Intraperitoneal exfoliative cytology

9.8.3.2.2 Records of tumor invasion depth

Tumor invasion depth is defined as follows:

□□TX: Unknown tumor invasion depth

□□T0: No evidence of primary tumor

□□T1: Tumor invasion is only confined to the mucosa (M) or the submucosal tissue (SM)

- T1a: Tumor invasion is only confined to the mucosa (M)
- T1b: Tumor invasion is confined to the submucosal tissue (SM)

□□T2: Tumor invasion exceeds the submucosal tissue but is only confined to the inherent muscular layer (MP)

□□T3: Tumor invasion exceeds the inherent muscular layer (MP) but is only confined to the subserosal tissue (SS)

□□T4: Tumor invasion involves the serosa (SE) or direct invasion of adjacent structures (SI)

- T4a: Tumor invasion involves only the serosa (SE)
- T4b: Tumor directly invades the adjacent structures (SI)

9.8.3.2.3 Records of tumor metastasis

(1) Lymph node metastasis:

□□NX: Number of lymph node metastases is unknown

□□N0: No lymph node metastasis

□□N1: Lymph node metastasis of 1-2 areas

□□N2: Lymph node metastasis of 3-6 areas

□□N3: Lymph node metastasis of 7 and more areas

- N3a: Lymph node metastasis of 7-15 areas
- N3b: Lymph node metastasis of 16 and more areas

Lymph node numbers are defined as follows:

No.	Name	Definition
1	Cardia right	Lymph nodes around the gastric wall first branch (cardia branch) of ascending branches of the left gastric artery and those at the cardia sides
2	Cardia left	Lymph nodes at the left side of the cardia and those along the cardia branch of the lower left diaphragmatic artery esophagus
3a	Lesser gastric curvature (along the left gastric artery)	Lymph nodes at the lesser curvature side along the left gastric artery branch, below the cardia branch
3b	Lesser gastric curvature (along the right gastric artery)	Lymph nodes at the lesser curvature side along the right gastric artery branch, partial left side of the 1st branch in the lesser curvature direction
4sa	Left side of the greater gastric curvature (short gastric artery)	Lymph nodes along the short gastric artery (excluding the root)
4sb	Left side of the greater gastric curvature (along the left gastroepiploic artery)	Lymph nodes along the left gastroepiploic artery and the first branch of the greater curvature (refer to the definition of No. 10)
4d	Right side of the greater gastric curvature (along the right gastroepiploic artery)	Lymph nodes at the partial left side of the first branch in the greater gastric curvature direction along the right gastroepiploic artery
5	Superior pylorus	Lymph nodes along the right gastric artery and around the first branch in the lesser gastric curvature direction
6	Inferior pylorus	Lymph nodes from the root of the right gastroepiploic artery to the first branch in the greater gastric curvature direction and those at the junction of the right gastroepiploic veins and superior anterior pancreaticoduodenal veins (including the junction portion)
7	Left gastric artery trunk	Lymph nodes from the root of the left gastric artery to the branch portion of the ascending branches
8a	Anterior upper part of the common hepatic artery	Lymph nodes at the anterior upper part of the common hepatic artery (from the branch portion of the splenic artery to the branch portion of the gastroduodenal artery)
8p	Posterior part of	Lymph nodes at the posterior part of the common hepatic artery

	the common hepatic artery	(from the branch portion of the splenic artery to the branch portion of the gastroduodenal artery)
9	Surrounding of the celiac artery	Lymph gland that is in the surroundings of the celiac artery or that is a part of each root of the left artery of the stomach, common hepatic artery and splenic artery as well as that related to the celiac artery
10	Splenic hilum	Lymph gland that is in the surroundings of the celiac artery and splenic hilum far away from the end of the pancreas, including the first greater gastric curvature in the root of the short gastric artery and the left gastroepiploic artery
11p	Splenic artery proximal	Lymph gland at the splenic artery proximal (in a location that divides the distance between the root of the splenic artery and the end of the pancreas into two equal parts, including the proximal side)
11d	Splenic artery distal	Lymph gland at the splenic artery distal (in a location that divides the distance between the root of the splenic artery and the end of the pancreas into two equal parts, inclining to the end of the pancreas)
12a	Within the hepatoduodenal ligament (along the proper hepatic artery)	Lymph gland that is below a location that divides the height of the confluence portions of the left and right hepatic ducts and the bile duct in the upper margin of the pancreas into two equal parts and is along the proper hepatic artery (as stated in No. 12a2 of the regulations for bile duct carcinoma)
12b	Within the hepatoduodenal ligament (along the bile duct)	Lymph gland that is below a location that divides the height of the confluence portions of the left and right hepatic ducts and the bile duct in the upper margin of the pancreas into two equal parts and is along the proper hepatic artery (as stated in No. 12b2 of the regulations for bile duct carcinoma)
12p	Within the hepatoduodenal ligament (along the portal vein)	Lymph gland that is below a location that divides the height of the confluence portions of the left and right hepatic ducts and the bile duct in the upper margin of the pancreas into two equal parts and is along the proper hepatic artery (as stated in No. 12p2 of the regulations for bile duct carcinoma)
13	Back of the pancreatic head	Lymph gland adjacent to the head of the duodenal papilla at the back of the pancreatic head (No. 12b in the surroundings of the hepatoduodenal ligament)
14v	Along the superior mesenteric vein	Lymph gland that is in the front of the superior mesenteric vein, with the inferior margin of the pancreas on the upper side, the right gastroepiploic vein and confluence portion of the superior pancreaticoduodenal vein to the right, the left margin of the mesenteric vein to the left and the branch of the middle colic vein in the lower margin
14a	Along the superior mesenteric	Lymph gland along the superior mesenteric artery

	artery	
15	Surroundings of the colon middle artery	Lymph gland that is in the surroundings of the colon middle artery
16a1	Surroundings of the abdominal aorta a1	Lymph gland that is in the surroundings of the aorta gap (4 to 5 cm wide in the surroundings of the medial crus of the diaphragm)
16a2	Surroundings of the abdominal aorta a2	Lymph gland that is in the surroundings of the aorta from the upper margin of the abdominal artery root to the lower margin of the left renal vein
16b1	Surroundings of the abdominal aorta b1	Lymph gland that is in the surroundings of the aorta from the lower margin of the left renal vein to the upper margin of the inferior mesenteric artery root
16b2	Surroundings of the abdominal aorta b2	Lymph gland that is in the surroundings of the aorta from the upper margin of the inferior mesenteric artery root to the branch of aorta
17	Front of the pancreatic head	Lymph gland that is in the front of the pancreatic head, next to the pancreas and under the pancreatic capsule
18	Below the pancreas	Lymph gland that is in the lower margin of the pancreas
19	Below the diaphragm	Lymph gland that is in the cavity of the diaphragm and along the lower side of the diaphragmatic artery
20	Hiatal part of the gullet	Lymph gland that connects the hiatal part of diaphragm to the gullet
110	Beside the lower gullet	Lymph gland that departs from the diaphragm and is next to the lower gullet
111	Above the diaphragm	Lymph gland that is in the cavity of the diaphragm and departs from the gullet (No. 20 that connects to the diaphragm and gullet)
112	Posterior mediastinum	Lymph gland of the posterior mediastinum departed from the gullet and its hiatal portion

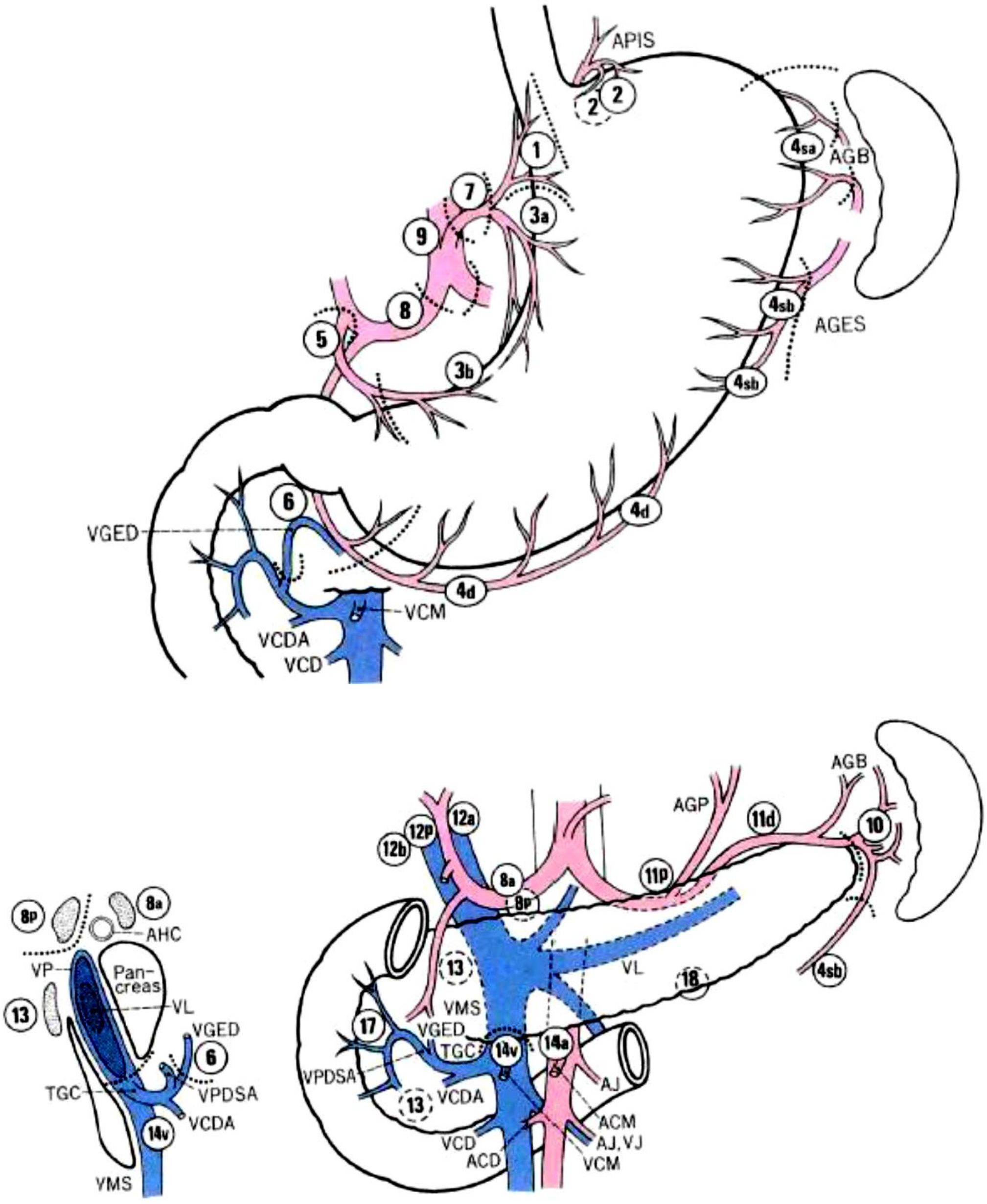

血管名
略字説明

APIS	A. phrenica inferior sinistra	AHC	A. hepatica communis
AGB	Aa. gastricae breves	VP	V. portae
AGES	A. gastroepiploica sinistra	VL	V. lienalis
VGED	V. gastroepiploica dextra	VMS	V. mesenterica superior
VCDA	V. colica dextra accessoria	VPDSA	V. pancreaticoduodenalis superior anterior
VCM	V. colica media	VCD	V. colica dextra
TGC	Truncus gastrocolicus	VJ	V. jejunalis
ACM	A. colica media	AGP	A. gastrica posterior
AJ	A. jejunalis	ACD	A. colica dextra

Fig. 4. Lymph node grouping

(2) Distant metastasis

□□M0: No distant metastasis outside of the regional lymph nodes

□□M1: Distant metastasis outside of the regional lymph nodes

□□MX: Presence of distant metastasis is unclear

Record the specific sites under the M1 condition: peritoneum (PER), liver (HEP), lymph node (LYM), skin (SKI), lung (PUL), bone marrow (MAR), bone (OSS), pleura (PLE), brain (BRA) and meninges (MEN), intraperitoneal exfoliated cells (CY), and others (OTH). Note: A positive examination result for intraperitoneal exfoliated cells is recorded as M1.

9.8.3.2.4 Tumor Staging

Pathological (pTNM)					
T/M	N0	N1	N2	N3a	N3b
T1	IA	IB	IIA	IIB	IIIB
T2	IB	IIA	IIB	IIIA	IIIB
T3	IIA	IIB	IIIA	IIIB	IIIC
T4a	IIB	IIIA	IIIA	IIIB	IIIC
T4b	IIIA	IIIB	IIIB	IIIC	IIIC
M1	IV	IV	IV	IV	IV

9.8.3.3 Pathologic types and classifications

9.8.3.3.1 Type

□□Papillary adenocarcinoma

□□Tubular adenocarcinoma

□□Mucinous adenocarcinoma

□□Signet ring cell carcinoma

□□Poorly differentiated carcinoma

9.8.3.3.2 Grading

□□GX classification is not possible to assess

□□G1 well-differentiated

□□G2 moderately differentiated

□□G3 poorly differentiated

□□G4 undifferentiated

9.8.3.4 Evaluation of Radical Level (Degree)

9.8.3.4.1 Recording the Presence or Absence of Tumor Invasion on the Resection Stump

(1) Proximal incisional margin (PM: proximal margin)

PM (-): No tumor invasion found on the proximal incisional margin

PM (+): Tumor invasion found on the proximal incisional margin

PM X: Unknown tumor invasion on the proximal incisional margin

(2) Distal incisional margin (DM: distal margin)

DM (-): No tumor invasion found on the distal incisional margin

DM (+): Tumor invasion found on the distal incisional margin

DM X: Unknown tumor invasion on the distal incisional margin

9.8.3.4.2 Radical Records

Postoperative residual tumor, denoted with R (residual tumor): R0: curative resection; R1, R2: non-curative resection.

RX: cannot be evaluated

R0: no residual tumor

R1: microscopic residual tumor (positive margins, peritoneal lavage cytology positive)

R2: macroscopic residual tumor

10 Statistical analysis

10.1 Definition of the population

(1) ITTP, intent-to-treat population

(2) MITTP, modified intent-to-treat population

(3) PPP, per-protocol population

(4) SAP, safety analysis population

10.2 Statistical analysis plan

- Statistical software: We will use Epidata3.0 to establish a database and to input data， and we will use SPSS statistical software, version 18.0 (SPSS Inc), and the R software environment (R Foundation for Statistical Computing) to perform statistical analyses.
- Basic principle： The method of differential testing was adopted. The safety population of the study consists of the patients who receive safety evaluation data after the intervention. Descriptive statistics and two-sided tests were conducted for the safety indicators and the incidence of adverse reactions. A p-value <0.05 is considered statistically significant. The confidence interval of the parameters is

estimated with a 95% confidence interval.

- Shedding analysis: Total shedding rate of two groups and loss rate due to adverse events will be compared using pearsonχ^2 test
- Statistical analysis of population division: baseline data and effective analysis using MITT analysis. The main therapeutic indicators are analyzed using both MITT and PP analysis. But based on the conclusion of MITT analysis. If MITT analysis and PP analysis of the conclusions are consistent, it can increase the credibility of the conclusion. The data of laboratory examination, adverse events and adverse reactions were analyzed by SAP. The incidence rate of adverse reactions uses SAP as the denominator.
- Method of outlier determination: the observation value is greater than P75 or less than P25, and the exceed value more than 3 times of the quartile spacing (=p75-p25), which will be sentenced to outlier data. During the analysis, the sensitivity analysis is used for outlier data, namely analyzing outcomes including or excluding, outlier's data. and if the results are not contradictory, the data is retained; if the contradiction, it depends on the specific circumstances.
- Descriptive statistics: The measurement data gives the mean, the standard deviation and the confidence interval, and the minimum value, the maximum value, the P25, the median and the P75 are given when necessary; matched data also gives the mean and standard deviation of the gap-value, and the median and average rank of the Non-parametric method. The nominal-scale data gives the frequency distribution and the corresponding percentages. The level data gives the frequency distribution and the corresponding percentages, as well as the median and the average rank. Qualitative data give positive rate, positive number, and denominator numbers. The survival data gives the number of events, the number of deletions, the median survival time, and the survival rate.
- Subgroup analysis：Sub-group analysis is to find the factors that may affect prognostic according to the specific circumstances of the data。
- Missing values handling：This study does not fill in missing values
- Effective analysis: Using Log-rank test for single factor analysis of Survival Time Data, using Cox regression model Analysis for multi-factor analysis. Quantitative data using t test or t' Test (variance is not homogeneous), qualitative data using

Pearson χ^2 test, grade data using Wilcoxon rank test.

- Safety analysis: counting adverse responds incidence and incidence of adverse events and make a list to describe the adverse events occurring in the study. describe the results of the laboratory tests before and after the normal/abnormal changes and the relationship between the abnormal changes and drugs in the research, and make a list on the "normal/abnormal" changes occurred in the study.. More detailed statistical analysis is shown in the statistical analysis plan.

11 Data management

11.1 Case Report Form (CRF)

11.1.1 CRF Types and Submission Deadline

CRFs used in this study and their submission deadlines are as follows:

(1) Case Screening: 7 days prior to surgery (time frame of three days)

(2) Enrolling: submitted to the data center at one day prior to surgery

(3) Surgery: within 1 day after surgery

(4) Postoperative discharge: within three days after the first discharge

(5) Follow-up records: 7 days after each specified follow-up time point

11.1.2 Method of transmission of CRF

In this study, the paper CRF form are used for information and data transmittal.

11.1.3 Revision of CRF

After the start of the study, if the CRF is found to lack items that are then deemed pertinent, under the premises of ensuring the amendment of the CRF does not cause medical and economic burden and increased risks to the selected patients, the CRF can be modified after the Research Committee adopt it through discuss at the meeting. If the amendment of the CRF requires no changes to this study protocol, the latter will not be modified.

11.2 Monitoring and Supervising

To assess whether study implementation follows protocol and data are being collected properly, monitoring should be conducted every February during the follow-up period. Monitoring is to complete through visiting a hospital and comparing the original Data.

11.2.1 Monitoring item

- Data Collection Completion Status: By selected registration numbers (cumulative and for each time period)

- Eligibility: Not eligible patients/potentially ineligible patients
- Different end of treatment, the reasons for suspension/end of the study protocol
- Background factors, pre-treatment report factors, post-treatment report factors when selected for registration
- Severe adverse events
- Adverse events/adverse reactions
- Laparoscopic surgery completion percentage
- Proportion of conversion to laparotomy
- Protocol deviation
- Disease-free survival /overall survival (all enrolled Patients)
- Progress and safety of the study, other issues

11.2.2 Acceptable range of adverse events

Treatment-related death and life-threatening complications caused by operations occur relatively rarely and partly are dependent on the qualifications of the participated hospitals and their staff; a rate of over 3% is considered unacceptable. If treatment-related death is suspected or non-hematologic Grade 4 toxicity having a causal relationship with the surgery is determined, adverse events should be reported to the Efficacy and Safety Evaluation Committee. If the number of treatment-related deaths or the number of patients with determined non-hematologic Grade 4 toxicity having a causal relationship with the surgery reached 2, the final incidence proportion of adverse events would be expected to exceed 3%, and therefore the inclusion of patients must be immediately suspended. Whether the study can continue should be determined by the Efficacy and Safety Evaluation Committee.

12 Relevant Provisions on adverse events

12.1 Surgery-related adverse events

See the adverse events mentioned for surgical complications in 8.1 Definition of the study endpoint.

12.2 Various forms of adverse events caused by original incidence

Adverse events relating to various forms of deterioration in primary diseases should be recorded according to Short Name of CTCAEv3.0.

12.3 Evaluation of adverse events

•□Evaluation of adverse event/adverse reaction are based on[Accordion Severity Grading System] and [CTCAE v3.0].

•□Adverse events will be graded 0 ~ 4 as per definition. For treatment-related death, fatal adverse events are classified as Grade 5 in the original CTCAE.

•□Toxicity items specified in the [surgery-related adverse events], Grade and the discovery date of Grade should be recorded in the treatment process report. For other toxicity items observed, observed Grade 3 toxicity items are only recorded in the freedom registration column of the treatment process report, as well as Grade and the discovery date of Grade. Grade recorded in the treatment process report must be recorded in the case report form.

•□CTCAE v3.0, the so-called “Adverse Event”, “all observed, unexpected bad signs, symptoms and diseases (abnormal value of clinical examination are also included) in the treatment or disposal, regardless of a causal relationship with the treatment or handling, including determining whether there is a causal relationship or not”.

•□Therefore, even if events were “obviously caused by primary disease (cancer)” or caused by supportive therapy or combination therapy rather than the study regimen treatment (protocol treatment), they are “adverse events”.

•□For adverse event data collection strategy, the following principles should be complied with in this study:1) Adverse events within 30 days from the last treatment day of the study regimen treatment (protocol treatment), regardless of the presence or absence of a causal relationship should be completely collected. (When adverse events are reported, the causality and classification of adverse events are separately discussed) 2) Adverse events within 30 days from the last treatment day of the study regimen treatment (protocol treatment), regardless of the presence or absence of a causal relationship should be completely collected. (When adverse events are reported, the causality and classification of adverse events are separately discussed).

12.4 Reporting of Adverse Events

- When “severe adverse events” or “unexpected adverse events” occur, the Research Responsible Person of research participating unit should report them to the Research Committee (Chang-Ming Huang).

•□Based on the relevant laws and regulations, adverse events should be reported to the province (city) Health Department at the location of the research center. Severe

adverse events based on clinical research-related ethical guideline should be reported to the person in overall charge of the medical institution. The appropriate reporting procedures should be completed in accordance with the relevant provisions of all medical institutions at the same time. The person in charge of research participating hospital should hold accountability and responsibility for the emergency treatment of patients with any degree of adverse events to ensure patient safety.

12.4.1 Adverse Events with Reporting Obligations

12.4.1.1 Adverse Events with Emergency Reporting Obligations

Any of the following adverse events should be reported on an emergent basis:

- All patients who die during the course of treatment or within 30 days from the last treatment day, regardless of the presence or absence of a causal relationship with the study regimen treatment. Also, cases of discontinuation of treatment, even if within 30 days from the last treatment day, those patients are also emergent reporting objects. ("30 days" refers to day 0, the final treatment day, 30 days starting from the next day)
- Those patients with unexpected Grade 4 non-hematologic toxicity (CTCAE v3.0 adverse events other than the blood/bone marrow group), having a causality of treatment (any of definite, probable, possible) who emergent reporting objects are.

12.4.1.2 Adverse Events with Regular Reporting Obligations

One of the following adverse events are regular reporting objects:

(1) After 31 days from the last treatment day, deaths for which a causal relationship with treatment cannot be denied, including suspected treatment-related death; death due to obvious primary disease is included.

(2) Expected Grade 4 non-hematologic toxicity (CTCAE v3.0 adverse events other than the blood/bone marrow group).

(3) Unexpected Grade 3adverse events: Grade 3 adverse events are not recorded in the 12.1 expected adverse events.

(4) Other significant medical events: adverse events that the study group deems cause Important and potentially permanent, significant impact on their offspring (MDS myelodysplastic syndrome, except for secondary cancer) Adverse events among above (2)-(4), determined to have a causal relationship (any of definite, probable, possible)

with the study regimen are regular reporting objects.

12.4.2 Reporting Procedure

12.4.2.1 Emergency Reporting

- In case of any adverse event on emergency study reporting objects, the doctor in charge will quickly report it to the Research Responsible Person of the research participating hospitals. When the Research Responsible Person of the hospital cannot be contacted, the coordinator or the doctor in charge of the hospital must assume the responsibility on behalf of the Research Responsible Person of the hospital.
- First Reporting: Within 72 hours after the occurrence of adverse events, the Research Responsible Person of the hospital should complete the “AE/AR/ADR first emergency report” and send it to the Research Committee by email and telephone.
- Second Reporting: The research responsible person of the research participating hospital completes the “AE/AR/ADR Report” and a more detailed case information report (A4 format), and then faxes the two reports to the Research Committee within 15 days after the occurrence of adverse events. If any autopsy examination, the autopsy result report should be submitted to the Research Committee.

12.4.2.2 General Reports

- The research responsible person of research participating hospital completes the “AE/AR/ADR report”, and then faxes it to the Research Committee within 15 days after the occurrence of adverse events.

12.5 Review of Efficacy and Safety Evaluation Committee

The Efficacy and Safety Evaluation Committee reviews and discusses the report in accordance with the procedures recorded in the *Clinical Safety Information Management Guideline*, and makes recommendations in writing for the Research Responsible Person, including whether to continue to include study objects or to modify the study protocol.

13 Ethical Considerations

13.1 Responsibilities of researchers

The investigators are responsible for the conduction of this study. The investigators will ensure the implementation of this study in accordance with the study

protocol and in compliance with the Declaration of Helsinki, as well as domestic and international ethical guiding principles and applicable regulatory requirements. It is specially noted that, the investigators must ensure that only subjects providing informed consent can be enrolled in this study.

13.2 Information and Informed Consent of Subjects

An unconditional prerequisite for subjects to participate in this study is his/her written informed consent. The written informed consent of subjects participating in this study must be given before study-related activities are conducted.

Therefore, before obtaining informed consent, the investigators must provide sufficient information to the subjects. In order to obtain the informed consent, the investigators will provide the information page to subjects, and the information required to comply with the applicable regulatory requirements. While providing written information, the investigators will orally inform the subjects of all the relevant circumstances of this study. In this process, the information must be fully and easily understood by non-professionals, so that they can sign the informed consent form according to their own will on the basis of their full understanding of this study.

The informed consent form must be signed and dated personally by the subjects and investigators. All subjects will be asked to sign the informed consent form to prove that they agree to participate in the study. The signed informed consent form should be kept at the research center where the investigator is located and must be properly safe kept for future review at any time during audit and inspection throughout the inspection period. Before participating in the study, the subjects should provide a copy of signed and dated informed consent form.

At any time, if important information becomes available that may be related to the consent of the subjects, the investigators will revise the information pages and any other written information which must be submitted to the IEC/IRB for review and approval. The revised information approved will be provided to each subject participating the study. The researchers will explain the changes made to the previous version of ICF to the subjects

13.3 Identity and Privacy of Subjects

After obtaining an informed consent form, each selected subject is assigned a subject number (Allocation Number). This number will represent the identity of the subject during the entire study and for the clinical research database of the study. The

collected data of subjects in the study will be stored in the ID.

Throughout the entire study, several measures will be taken to minimize any breaches of personal information, including: 1) only the investigators will be able to link to the research data of the subjects to themselves through the identify table kept at the research center after authorization; 2) during onsite auditing of raw data by the supervisors of this study, as well as relevant inspection and inspection visits by the supervision departments, the personnel engaging in the above activities may view the original medical information of subjects that will be kept strictly confidential.

Collection, transmission, handling and storage of data on study subjects will comply with the data protection and privacy regulations. This information will be provided to the study subjects when their informed consent is being obtained for treatment procedures in accordance with national regulations.

13.4 Independent Ethics Committee or Institutional Review Committee

Before beginning the study, the research center will be responsible for submitting the study protocol and relevant documents (informed consent form, subject information page, CRF, and other documents that may be required) to the Independent Ethics Committee (IEC)/ Institutional Review Board (IRB) to obtain their favorable opinion/approval. The favorable opinions/approval documents of the IEC/IRB will be archived in the research center folders of the investigators.

Before beginning the study at the center, the investigators must obtain written proof of favorable opinions/approval by the IEC/IRB, and should provide written proof of the date of the favorable opinions/approval meeting, written proof of the members presenting at the meeting and voting members, written proof of recording the reviewed study, protocol version and Informed Consent Form version, and if possible, a copy of the minutes.

In case of major revisions to this study, the amendment of the study protocol will be submitted to the IEC/IRB prior to performing the study. In the course of the study, the relevant safety information will be submitted to the IEC/IRB in accordance with national regulations and requirements.

13.5 Supervising

The research approach of the authorities and any associated files (such as the research protocol, subjects' informed consent) will be in accordance with the requirements of the ethical review board of biomedical research involving humans trial

(2007) and the applicable Chinese laws and regulations. Studies should provide the main references or inform the ethics review guidance advisory organization of the provincial health administrative department in the province the research center is in.

14 Organizations and Responsibilities of Study

14.1 Research Committee

- Responsible for developing study protocol, auditing eligibility for inclusion and guiding the interpretation of informed consent; also responsible for the collection of adverse event reports, guiding the clinical diagnosis and treatment of such events and the emergency intervention of serious adverse events.
- Person in Charge of Research Committee: Chang-Ming Huang (Department of Gastric Surgery, Fujian Medical University Union Hospital)

  Add: Department of Gastric Surgery, Fujian Medical University Union Hospital, No.29 Xinquan Road, Fuzhou 350001, Fujian Province, China; Post code:350001; Tel: 0591-83357896-8011; Fax: 0591-83363366; Mobile: 13805069676; E-mail: hcmlr2002@163.com
- Chief Statistical Expert of Research Committee: Zhi-Jian Hu (Department of Preventive Medicine statistics, School of Public health, Fujian Medical University)

14.2 Efficacy and Safety Evaluation Committee

Responsible for the supervision/monitoring of treatment safety and efficacy of this study.

- Person in Charge of Efficacy and Safety Evaluation Committee: Chang-Ming Huang (Department of Gastric Surgery, Fujian Medical University Union Hospital)

14.3 Independent Ethics Committee/Institutional Review Board (IEC/IRB)

 Responsible for evaluating this study to determine if risks to which subjects are exposed have been duly minimized and whether these risks are reasonable compared to expected benefits. (IRB number: 2016YF009-02)

15 References

1.Lin J X, Huang C M, Zheng C H, et al. Evaluation of laparoscopic total gastrectomy for advanced gastric cancer: results of a comparison with laparoscopic distal gastrectomy[J]. Surgical endoscopy, 2016, 30: 1988-1998.

2.Kim H S, Kim B S, Lee I S, et al. Laparoscopic gastrectomy in patients with previous

gastrectomy for gastric cancer: a report of 17 cases[J]. Surgical Laparoscopy Endoscopy & Percutaneous Techniques, 2014, 24(2): 177-182.

3.Kwon I G, Cho I, Guner A, et al. Minimally invasive surgery for remnant gastric cancer: a comparison with open surgery[J]. Surgical endoscopy, 2014, 28: 2452-2458.

4.Ota M, Ikebe M, Shin Y, et al. Laparoscopic total gastrectomy for remnant gastric cancer: A single-institution experience and systematic literature review[J]. in vivo, 2020, 34(4): 1987-1992.

5.Bray F, Ferlay J, Soerjomataram I, Siegel RL, Torre LA, Jemal A. Global cancer statistics 2018: GLOBOCAN estimates of incidence and mortality worldwide for 36 cancers in 185 countries. CA: a cancer journal for clinicians. 2018.

6.Chen W, Zheng R, Baade PD, et al. Cancer statistics in China, 2015. CA: a cancer journal for clinicians. 2016;66(2):115-132.

7.Welvaart K, Warnsinck H M. The incidence of carcinoma of the gastric remnant [J]. J Surg Oncol, 1982, 21(2): 104-106.

8.Nozaki I, Nasu J, Kubo Y, et al. Risk factors for metachronous gastric cancer in the remnant stomach after early cancer surgery [J]. World J Surg, 2010, 34(7): 1548-1554.

9.Kaneko K, Kondo H, Saito D, et al. Early gastric stump cancer following distal gastrectomy [J]. Gut, 1998, 43(3): 342-344.

10.Sinning C, Schaefer N, Standop J, et al. Gastric stump carcinoma - epidemiology and current concepts in pathogenesis and treatment [J]. Eur J Surg Oncol, 2007, 33(2): 133-139.

11.Yamada H, Kojima K, Yamashita T, et al. Laparoscopy-assisted resection of gastric remnant cancer [J]. Surg Laparosc Endosc Percutan Tech, 2005, 15(4): 226-229.

12.Imada T, Rino Y, Hatori S, et al. Clinicopathologic differences between early gastric remnant cancer and early primary gastric cancer in the upper third of the stomach [J]. Hepatogastroenterology, 2000, 47(34): 1186-1188.

13.Inomata M, Shiraishi N, Adachi Y, et al. Gastric remnant cancer compared with primary proximal gastric cancer [J]. Hepatogastroenterology, 2003, 50(50): 587-591.

14.Son S Y, Lee C M, Jung D H, et al. Laparoscopic completion total gastrectomy for remnant gastric cancer: a single-institution experience [J]. Gastric Cancer, 2015, 18(1): 177-182.

15.Nagai E, Nakata K, Ohuchida K, et al. Laparoscopic total gastrectomy for remnant gastric cancer: feasibility study [J]. Surg Endosc, 2014, 28(1): 289-296.

16.Jiang X, Hiki N, Nunobe S, et al. Laparoscopy-assisted subtotal gastrectomy with very small remnant stomach: a novel surgical procedure for selected early gastric cancer in the upper stomach [J]. Gastric Cancer, 2011, 14(2): 194-199.

17.Tsunoda S, Okabe H, Tanaka E, et al. Laparoscopic gastrectomy for remnant gastric cancer: a comprehensive review and case series[J]. Gastric Cancer, 2016, 19: 287-292.

18.Park Y K, Yoon H M, Kim Y W, et al. Laparoscopy-assisted versus open D2 distal gastrectomy for advanced gastric cancer: results from a randomized phase II multicenter clinical trial (COACT 1001)[J]. 2018.

19.Busweiler L A D, Schouwenburg M G, van Berge Henegouwen M I, et al. Textbook outcome as a composite measure in oesophagogastric cancer surgery[J]. Journal of British Surgery, 2017, 104(6): 742-750.

20.Clavien PA, Barkun J, de Oliveira ML, Vauthey JN, Dindo D, Schulick RD, et al. The Clavien-Dindo classification of surgical complications: five-year experi ence. Ann Surg 2009;250(2):187e96.

21.Wei S, Radwan A, Mueck KM, Wan C, Wan DQ, Millas SG, et al. Validation of the adapted clavien-dindo in trauma (ACDiT) classifications in medical and surgical management of acute diverticulitis. Ann Surg 2022;275(2):e415e9. https://doi.org/10.1097/SLA.0000000000003888

22.Martin JA, Regehr G, Reznick R, et al. Objective structured assessment of technical skill (OSATS) for surgical residents. The British journal of surgery. 1997;84(2):273-278.

23.Bonrath EM, Zevin B, Dedy NJ, Grantcharov TP. Error rating tool to identify and analyse technical errors and events in laparoscopic surgery. The British journal of surgery. 2013;100(8):1080-1088.

24.Wilson MR, Poolton JM, Malhotra N, Ngo K, Bright E, Masters RSW. Development and Validation of a Surgical Workload Measure: The Surgery Task Load Index

(SURG-TLX). World Journal of Surgery. 2011;35(9):1961-1969.

16 Annex

16.1 Informed Consent Form
