# Supplementary material for: The safety, feasibility, and oncological outcomes of laparoscopic completion total gastrectomy for remnant gastric cancer: a prospective study with 3-year follow-up (FUGES-004 study)
Source: Int J Surg. 2024 Apr 9;110(6):3382–91. doi: 10.1097/JS9.0000000000001388 (PMC11175827; doi:10.1097/JS9.0000000000001388)
Supplement: Supplementary file 3 [file js9-110-3382-s003.docx]

**Supplementary Definition and Statistical method**

**Study Patients**

The **FUGES-004** study enrolled patients scheduled to radical LCTG with D2 lymphadenectomy. Eligibility criteria included age between 18 and 75 years; an Eastern Cooperative Oncology Group score of 0 (asymptomatic) or 1 (symptomatic but completely ambulatory); an American Society of Anesthesiology score of class I, II, or III; presence of carcinomas arising from the remnant stomach following distal gastrectomy, irrespective of the histology of the primary lesion (benign or malignant) or risk of recurrence, extent of resection, or method of reconstruction (Billorth-I or Billorth-II) and diagnosed as preoperative clinical stage of cT1 to cT4a, N0/+, M0 according to the AJCC 7^th^ Manual^1^. Exclusion criteria encompassed distant metastasis; history of previous upper abdominal surgery (except laparoscopic cholecystectomy, previous gastrectomy, endoscopic mucosal resection, or endoscopic submucosal dissection); other malignant diseases (except gastric cancer) within the past 5 years; and enlarged regional lymph nodes exceeding 3 cm in diameter on preoperative imaging. The details on eligibility criteria are summarized in **eTable 1**.

**Routine follow-up procedures**

Follow-up appointments were conducted every 3 months for the first 2 years and every 6 months for the subsequent 3 years. Routine follow-up procedures included (1) physical examination and blood testing at each follow up; (2) chest radiography and abdominal computed tomography scans every 6 months for 3 years; and (3) upper gastrointestinal endoscopy annually for 3 years. Positron emission tomography/computed tomography was performed for patients with suspected recurrence. Recurrence was identified through medical history, physical examination, imaging, cytology, and tissue biopsy (when feasible). Those exhibiting symptomatic indicators such as abdominal mass, weight loss, or obstruction concurrent with recurrence were evaluated regardless of their scheduled follow up.

**Statistical Analysis**

***Inverse Probability of Treatment Weighting***

Inverse probability of treatment weighting (IPTW) was used to adjust for confounding between the intervention and control groups^2-5^. First, calculating the probability (so called propensity score) of assigning to the intervention group based on patients’ characteristics. Second, the propensity scores were weighted to each patient to balance the baseline characteristics of the intervention and control groups. The application of these weights to the study population creates a pseudopopulation in which confounders are equally distributed across groups. In this study, 28 covariates (**eTable 5**), including patient characteristics and tumor findings, were identified. Investigators blinded to the outcome reviewed and checked the medical records, stored images, and laboratory data of all patients. A blinded biostatistician (LY.Z.) performed the IPTW using generalized boosted method (GBM)^6^ to estimate the propensity score of each patient based on the following variables: age, histology, pathological T stage, pathological N stage, tumor location, tumor size, neoadjuvant chemotherapy and adjuvant chemotherapy. Each patient was weighted by propensity score when comparing the short- and long-term oncological outcomes between the LCTG and OCTG groups. The balance of covariates was assessed using a standardized mean difference (SMD) approach. Factors with an imbalance between the two groups were defined as SMD>0.1.

The adjusted Kaplan–Meier curves and log-rank test, based on IPTW, were computed to compare 3-year OS rates, 3-year DFS rates, and cumulative hazard of recurrence between the two groups. Hazard ratios (HRs) comparing the LCTG and OCTG were estimated using Cox regression after confirming the proportional hazards assumption. Multivariable Cox regression analyses were performed to evaluate the effect of the surgery type on survival and recurrence after adjusting for clinicopathological covariates that were significantly associated with outcomes in univariable analyses. Subgroup analyses were performed to investigate the HR of LCTG versus OCTG for DFS, OS, and recurrence based on age, sex, comorbidities, tumor location, histology, pathological T and N stages, and adjuvant chemotherapy. Additionally, concurrent comparisons were performed to eliminate time-frame bias between LCTG and OCTG.

Statistical analysis were analyzed through *SPSS statistical* software, version 25.0 (SPSS Inc), and the *R software* version 4.2.0 (R Foundation for Statistical Computing) from March to September 2023.

**Reference**

1.Edge  SB, Byrd  DR, Compton  CC, Fritz  AG, Greene  FL, Trotti  A, eds.  AJCC Cancer Staging Manual. 7th ed. Springer; 2010.

2.Austin PC. An introduction to propensity score methods for reducing the effects of confounding in observational studies. Multivariate Behav Res 2011; 46: 399-424.

3.Austin PC. Variance estimation when using inverse probability of treatment weighting (IPTW) with survival analysis.[J] .Stat Med, 2016; 35: 5642–55.

4.Bonnot PE, Piessen G, Kepenekian V, et al. Cytoreductive Surgery With or Without Hyperthermic Intraperitoneal Chemotherapy for Gastric Cancer With Peritoneal Metastases (CYTO-CHIP study): A Propensity Score Analysis. J Clin Oncol. 2019;37(23):2028-2040.

5.Shin HJ, Son SY, Wang B, Roh CK, Hur H, Han SU. Long-term Comparison of Robotic and Laparoscopic Gastrectomy for Gastric Cancer: A Propensity Score-weighted Analysis of 2084 Consecutive Patients. Ann Surg. 2021;274(1):128-137.

6.Hu L, Gu C, Lopez M, Ji J, Wisnivesky J. Estimation of causal effects of multiple treatments in observational studies with a binary outcome. Stat Methods Med Res. 2020;29(11):3218-3234.
