# Supplementary material for: The safety, feasibility, and oncological outcomes of laparoscopic completion total gastrectomy for remnant gastric cancer: a prospective study with 3-year follow-up (FUGES-004 study)
Source: Int J Surg. 2024 Apr 9;110(6):3382–91. doi: 10.1097/JS9.0000000000001388 (PMC11175827; doi:10.1097/JS9.0000000000001388)
Supplement: Supplementary file 4 [file js9-110-3382-s004.docx]

# The Safety, Feasibility and Oncological Outcomes of Laparoscopic Completion Total Gastrectomy for Remnant Gastric Cancer:

# *A prospective study* (FUGES-004 study)

**eTable 1.** Eligibility Criteria in the FUGES-004 Study.

**eTable 2.** The distribution of patients in each center.

**eTable 3.** Checklist for determination of success about D2 lymphadenectomy.

**eTable 4.** Short-term outcomes between FJMUUH and Other external cohorts.

**eTable 5.** Basic Information of previous literature.

**eTable 6.** Preoperative Items Isolated to Estimate Propensity Scores.

**eTable 7.** Surgical Outcomes and Recovery of patients with RGC after IPTW.

**eTable 8.** Uni-variate and multi-variate analysis of the Overall Survival for RGC patients.

**eTable 9.** Mean recurrence time of patients with RGC before and after IPTW.

**eTable 10.** Uni-variate and multi-variate analysis of the Recurrence-free Survival for RGC patients.

**eTable 11.** Concurrent comparison of postoperative morbidity between LCTG and OCTG after IPTW.

**eTable 12.** Concurrent comparison of Surgical Outcomes and Recovery between LCTG and OCTG after IPTW.

**eTable 13.** Concurrent comparison of mean recurrence time between LCTG and OCTG before and after IPTW.

**eTable 14.** Per-protocol analysis of postoperative morbidity between LCTG and OCTG after IPTW.

**eTable 15.** Per-protocol analysis of Surgical Outcomes and Recovery between LCTG and OCTG after IPTW.

**eTable 1. Eligibility Criteria in the FUGES-004 Study**

| **Inclusion Criteria** |
| --- |
| Age between 18 and 75 years |
| Carcinomas arise in the remnant stomach following distal gastrectomy, irrespective of the histology of the primary lesion (benign or malignant) or its risk of recurrence, the extent of resection, or method of reconstruction (Billorth-I or Billorth-II). |
| cT1-4a, N-/+, M0 at preoperative evaluation according to the AJCC Cancer Staging Manual, 7th Edition |
| Performance status of 0 or 1 on ECOG scale |
| ASA class I, II, or III |
| Underwent radical LCTG with D2 lymphadenectomy |
| Written informed consent |
| **Exclusion Criteria** |
| Distant metastasis in the preoperative examinations |
| Previous upper abdominal surgery (except laparoscopic cholecystectomy, previous gastrectomy, endoscopic mucosal resection or endoscopic submucosal dissection) |
| Other malignant diseases (except gastric cancer) within the past 5 years |
| Enlarged or bulky regional lymph node (diameter over 3cm) supported by preoperative imaging including enlarged or bulky No.10 lymph nodes |
| Women during breast-feeding or pregnancy |
| Severe mental disorder |
| Unstable myocardial infarction, angina, or cerebrovascular accident within the past 6 months |
| History of continuous systematic administration of corticosteroids within one month |
| FEV1<50% of predicted values |
| Requirement of simultaneous surgery for other disease |
| Emergency surgery due to complication (bleeding, obstruction or perforation) caused by gastric cancer |

Abbreviations: AJCC, American Joint Committee on Cancer; ASA, American Society of Anesthesiology; ECOG PS, Eastern Cooperative Oncology Group performance status; FEV1, forced expiratory volume in 1 second.

**eTable 2. The distribution of patients in each center**

| **Participated tertiary referral teaching hospital** | **LCTG (N=46)** | **OCTG (N=160)** |
| --- | --- | --- |
| Fujian Medical University Union Hospital | 46 (**FUGES-004** trial) | 79 |
| First Affiliated Hospital of Fujian Medical University | / | 40 |
| Zhangzhou Affiliated Hospital of Fujian Medical University | / | 5 |
| Longyan First Affiliated Hospital of Fujian Medical University | / | 6 |
| First Affiliated Hospital of Xiamen University | / | 4 |
| Shanxi Provincial Cancer Hospital | / | 26 |

**eTable 3. Checklist for determination of success about D2 lymphadenectomy**

| Scoring Method for D2 Lymph Node Dissection (if available, not all following steps are included in the LCTG for patients with RGC) | Complete Incomplete None  10 5 0 |
| --- | --- |
| 1. Properly full omentectomy  2. Ligation of left gastroepiploic artery at origin  3. Ligation of right gastroepiploic artery at origin  4. Full exposure of common hepatic artery  5. Ligation of right gastric artery at origin  6. Exposure of portal vein  7. Exposure of splenic artery to branch of posterior gastric artery  8. Identification of splenic vein  9. Ligation of left gastric artery at origin  10. Exposure of gastroesophageal junction | □ □ □  □ □ □  □ □ □  □ □ □  □ □ □  □ □ □  □ □ □  □ □ □  □ □ □  □ □ □ |

1. Properly full omentectomy

a. Omentectomy was performed close to transverse colon

b. Omentectomy was performed from hepatic flexure to splenic flexure

c. Anterior layer of transverse colonic mesentery and pancreatic anterior peritoneum was dissected.

2. Ligation of left gastroepiploic artery at origin

3. Ligation of right gastroepiploic artery at origin

4. Full exposure of common hepatic artery

a. More than half of anterior part in the common hepatic artery were exposed.

5. Ligation of right gastric artery at origin

6. Exposure of portal vein

7. Exposure of splenic artery

a. Anterior part in splenic artery was exposed.

b. Splenic artery was exposed from celiac trunk to the terminal branch of the splenic artery

8. Identification of splenic vein

9. Ligation of left gastric artery at origin

10. Exposure of gastroesophageal junction

a. Anterior, posterior, left and right side of the abdominal esophagus were exposed.

- D2 lymphadenectomy was accepted if all randomly assigned three investigators rated more than 80% of total points regarding checklists in unedited video review.

**eTable 4. Short-term outcomes between FJMUUH and Other centers**

|  | **Other centers (N=81)** | **FJMUUH cohort (N=79)** | **P-value** |
| --- | --- | --- | --- |
| **Surgical Outcome** |  |  |  |
| Blood loss, ml | 216.1 ± 192.3 | 238.0 ± 219.9 | 0.892 |
| The number of harvest lymph nodes, n | 16.1 ± 11.2 | 15.0 ± 7.3 | 0.658 |
| Combined organ resection, n (%) | 13 (16.0%) | 14 (17.7%) | 0.778 |
| **Overall morbidity, n (%)** | 32 (39.5%) | 26 (32.9%) | 0.386 |
| **Surgical morbidity** |  |  |  |
| Anastomotic leak, n (%) | 7 (8.6%) | 4 (5.1%) | 0.371 |
| Wound infection, n (%) | 12 (14.8%) | 6 (7.6%) | 0.148 |
| Ileus and gastroparesis, n (%) | 3 (3.7%) | 4 (5.1%) | 0.674 |
| Abdominal or anastomotic bleeding, n (%) | 4 (4.9%) | 1 (1.3%) | 0.182 |
| Abdominal abcess, n (%) | 3 (3.7%) | 5 (6.3%) | 0.446 |
| **Medical morbidity** |  |  |  |
| Urinary complications, n (%) | 1 (1.2%) | 1 (1.3%) | 0.986 |
| Respiratory complication, n (%) | 20 (24.7%) | 17 (21.5%) | 0.634 |
| Cerebrocardiovascular complication, n (%) | 1 (1.2%) | 1 (1.3%) | 0.986 |
| **Mortality, n (%)** | 0 (0.0%) | 1 (1.3%) | 0.310 |

**eTable 5. Basic Information of previous literature**

| **Author** | **Nation** | **Study Type** | **Journal** | **Publication** | **Study Interval** | **Sample Size**  **(LG vs. OG)** | |
| --- | --- | --- | --- | --- | --- | --- | --- |
| ***Current study*** | ***China*** | ***Prospective*** | ***/*** | ***/*** | ***2003-2020*** | ***46*** | ***160*** |
| Ryuhei Aoyama^13^ | Japan | Retrospetive | Annals of Surgical Oncology | 2022 | 2005-2020 | 132 (93) | 195 (93) |
| Junya Kitadani^14^ | Japan | Retrospetive | Medicine | 2021 | 2008-2018 | 23 | 15 |
| Eisuke Booka^3^ | Japan | Retrospetive | Asian J Endosc Surg | 2019 | 2007-2017 | 8 | 23 |
| Masaki Kaihara^15^ | Japan | Retrospetive | Surg Case Rep | 2019 | 2007-2017 | 6 | 15 |
| Mitsuhiko Ota^16^ | Japan | Retrospetive | In Vivo | 2019 | 2005-2018 | 7 | 15 |
| Su Jung Choi^17^ | Korea | Retrospetive | J Minim Invasive Surg | 2019 | 2008-2016 | 21 | 139 |
| Y U Nakaji^18^ | Japan | Retrospetive | Anticancer Res | 2019 | 2004-2014 | 4 | 18 |
| Ryota Otsuka^19^ | Japan | Retrospetive | Ann Gastroenterol Surg | 2018 | 2008-2017 | 7 | 20 |
| Luo GD^20^ | China | Retrospetive | Int J Clin Exp Med. 8 | 2015 | 2007-2014 | 9 | 9 |
| Sang-Yong Son^21^ | Korea | Retrospetive | Gastric Cancer | 2015 | 2003-2012 | 17 | 17 |
| In Gyu Kwon^4^ | Korea | Retrospetive | Surg Endosc | 2014 | 2005-2012 | 18(10) | 58 |
| Nagai et al.^22^ | Japan | Retrospetive | Surg Endosc | 2013 | 1996-2012 | 12 | 10 |
| Park et al.^23^ | Korea | Retrospetive | J Korean Surg Soc | 2008 | / | 4 | 4 |
| Erica Sakamoto et al^.24^ | Brazil | Case series | J Laparoendosc Adv Surg Tech A | 2021 | 2009-2019 | 8 | / |
| Tsunoda S et al.^25^ | Japan | Case series | Gastric Cancer | 2016 | 2005-2014 | 10 | / |
| Yamamoto M et al.^26^ | Japan | Case series | Hepatogastroenterology | 2015 | 2008-2014 | 3 | / |
| Korehisa S et al.^27^ | Japan | Case series | Anticancer Res | 2015 | / | 4 | / |
| Pan, Y et al.^28^ | China | Case series | World J Surg Oncol | 2014 | / | 3 | / |
| Kim, H S et al.^29^ | Korea | Case series | Surg Laparosc Endosc Percutan Tech | 2014 | 2008-2012 | 17 | 50 |
| Liu HB et al.^30^ | China | Case series | Chin J Dig Surg | 2013 | / | 18 | / |
| Shinohara et al.^31^ | Japan | Case series | Langenbecks Arch Surg | 2012 | 2010-2012 | 5 | / |
| Li P et al.^32^ | China | Case series | J Dig Oncol. | 2011 | 2008-2010 | 16 | / |
| Qian F et al.^33^ | China | Case series | Surg Endosc | 2010 | 2006-2009 | 15 | / |
| Cho HJ et al. ^34^ | Korea | Case series | Surg Laparosc Endosc Percutan Tech | 2009 | / | 2 | / |
| Corcione F et al. ^35^ | Italy | Case series | Surg Laparosc Endosc Percutan Tech | 2008 | 2003-2005 | 3 | / |
| Hiroyuki Yamada et al.^5^ | Japan | Case series | Surg Laparosc Endosc Percutan Tech | 2005 | 2005 | 1 | / |

**eTable 6. Preoperative Items Isolated to Estimate Propensity Scores**

| **Patient information** |
| --- |
| Age |
| Gender |
| Performance status (ECOG) |
| ASA physical status classification |
| Height |
| Body weight |
| Operating history |
| FEV1 (% predicted) |
| **Risk, comorbidities or symptoms** |
| Diabetes melitus |
| Anemia |
| Stenosis |
| Pain |
| Fever |
| Pulmonary function disorder |
| Respiratory disorder |
| Renal function disorder |
| Liver function disorder |
| Neurological disorder |
| Hypertension |
| Gallbladder stone |
| Other operative risks |
| History of smoking |
| History of alcohol use |
| **Tumour characteristics** |
| Location (upper or middle) |
| Cross-sectional location (anterior, posterior, lesser curvature, or multiple) |
| Tumour size |
| Macroscopic type |
| Histological type of biopsy specimens |

Abbreviations: American Society of Anesthesiologist; ECOG PS, Eastern Cooperative Oncology Group performance status; FEV1, Forced expiratory volume in 1 second.

**eTable 7. Surgical Outcomes and Recovery of patients with RGC after IPTW**

| **Mean (SD)** | **OCTG (N=158.9)** | **LCTG (N=43.4)** | **P value** |
| --- | --- | --- | --- |
| **Surgical Outcomes** |  |  |  |
| Blood loss, ml | 220.4 ± 197.7 | 59.7 ± 86.7 | **<0.001** |
| Time of operation, min | 225.7 ± 104.9 | 163.9 ± 37.1 | **<0.001** |
| The number of harvest lymph nodes, n | 15.6 ± 9.5 | 19.2 ± 11.0 | **0.030** |
| Multiviceral resections, n (%) | 26.3 (16.6%) | 3.6 (8.3%) | 0.288 |
| **Recovery from surgery** |  |  |  |
| Removal of intra-abdominal drains, days | 11.8 ± 11.0 | 7.7 ± 3.0 | **0.014** |
| Initiation of solid food intake, days | 6.8 ± 7.3 | 4.9 ± 2.9 | 0.095 |
| Time to Flatus, days | 4.1 ± 2.5 | 3.5 ± 2.1 | 0.123 |
| Discharge from the hospital (postoperative), days | 18.7 ± 11.3 | 11.9 ± 6.0 | **<0.001** |

**eTable 8. Uni-variate and multi-variate analysis of the Overall Survival for RGC patients**

|  | **Univariate analysis** | | | **Multivariate analysis** | | | |
| --- | --- | --- | --- | --- | --- | --- | --- |
|  | **HR** | **95% CI** | **P value** | **HR** | **95% CI** | **P value** | |
| **Age** |  |  |  |  |  |  | |
| <65 years | 1 |  | |  |  |  | |
| ≥65 years | 1.18 | 0.8-1.75 | 0.408 |  |  |  | |
| **Sex** |  |  | |  |  |  | |
| Male | 1 |  | |  |  |  | |
| Female | 1.64 | 0.97-2.76 | 0.065 |  |  |  | |
| **Comorbidity** |  |  | |  |  |  | |
| No | 1 |  | |  |  |  | |
| Yes | 1.04 | 0.7-1.53 | 0.856 |  |  |  | |
| **ASA scores** |  |  | |  |  |  | |
| I | 1 |  | |  |  |  | |
| II-III | 0.80 | 0.47-1.37 | 0.413 |  |  |  | |
| **Surgical approaches** |  |  | |  |  |  | |
| OCTG | 1 |  | |  |  |  | |
| LCTG | 0.77 | 0.45-1.30 | 0.323 |  |  |  | |
| **Histology** |  |  | |  |  |  | |
| Differentiated | 1 |  | | 1 |  |  | |
| Undifferentiated | 2.06 | 1.33-3.2 | **0.001** | 1.70 | 1.07-2.71 | **0.025** | |
| **Pathologic T Stage** |  |  | |  |  |  | |
| T1 | 1 |  | | 1 |  |  | |
| ≥T2 | 3.43 | 1.5-7.83 | **0.003** | 2.22 | 0.92-5.35 | 0.075 | |
| **Pathologic N Stage** |  |  | |  |  |  | |
| N0 | 1 |  | | 1 |  |  | |
| N+ | 0.46 | 0.3-0.69 | **<0.001** | 0.63 | 0.40-1.00 | **0.048** | |
| **Tumor location** |  |  | |  |  |  | |
| Anastomoic site | 1 |  | |  |  |  | |
| Non-anastomoic site | 1.01 | 0.68-1.49 | 0.964 |  |  |  | |
| **Tumor Size** | 1.02 | 1.01-1.02 | **0.001** | 1.01 | 1.00-1.02 | 0.258 | |
| **LVI** |  |  | |  |  |  | |
| No | 1 |  |  |  |  |  | |
| Yes | 1.58 | 1.07-2.33 | **0.023** | 1.02 | 0.66-1.58 | 0.921 | |
| **Neoadjuvant chemotherapy** |  |  | |  | |  |  |
| No | 1 |  | |  | |  |  |
| Yes | 1.14 | 0.65-2 | 0.653 |  | |  |  |
| **Adjuvant chemotherapy n (%)** |  |  | |  | |  |  |
| No | 1 |  | |  | |  |  |
| Yes | 1.37 | 0.92-2.04 | 0.125 |  | |  |  |

**eTable 9. Mean recurrence time of patients with RGC before and after IPTW**

|  | **Time to recurrence, months** | | | | | |
| --- | --- | --- | --- | --- | --- | --- |
|  | **Before IPTW** | | | **After IPTW** | | |
|  | **OCTG (N=160)** | **LCTG (N=46)** | **P value** | **OCTG (N=158.9)** | **LCTG (N=43.4)** | **P value** |
| Overall | 22.5 ± 29.4 | 13.8 ± 8.7 | 0.568 | 22.1 ± 29.1 | 15.0 ± 9.1 | 0.357 |
| Local recurrence | 29.5 ± 39.4 | 6.0 ± 1.4 | 0.116 | 28.4 ± 38.2 | 5.7 ± 2.0 | 0.514 |
| Peritoneal recurrence | 22.5 ± 36.6 | 16.7 ± 10.2 | 0.589 | 20.6 ± 35.1 | 16.8 ± 9.9 | 0.769 |
| Distant metastasis | 22.4 ± 26.7 | 12.2 ± 7.9 | 0.238 | 21.7 ± 26.0 | 11.3 ± 7.7 | 0.233 |

**eTable 10. Uni-variate and multi-variate analysis of the Recurrence-free Survival for RGC patient**

|  | Univariate analysis | | | Multivariate analysis | | |
| --- | --- | --- | --- | --- | --- | --- |
|  | HR | 95% CI | P value | HR | 95% CI | P value |
| Age |  |  |  |  |  |  |
| <65 years | 1 |  |  |  |  |  |
| ≥65 years | 0.95 | 0.59-1.53 | 0.845 |  |  |  |
| Sex |  |  |  |  |  |  |
| Male | 1 |  |  |  |  |  |
| Female | 1.62 | 0.85-3.09 | 0.142 |  |  |  |
| Comorbidity |  |  |  |  |  |  |
| No | 1 |  |  |  |  |  |
| Yes | 0.99 | 0.61-1.59 | 0.956 |  |  |  |
| ASA scores |  |  |  |  |  |  |
| I | 1 |  |  |  |  |  |
| II-III | 0.81 | 0.42-1.60 | 0.551 |  |  |  |
| Surgical approaches |  |  |  |  |  |  |
| OCTG | 1 |  |  |  |  |  |
| LCTG | 0.89 | 0.50-1.59 | 0.697 |  |  |  |
| Histology |  |  |  |  |  |  |
| Differentiated | 1 |  |  |  |  |  |
| Undifferentiated | 2.08 | 1.21-3.55 | **0.008** | 1.64 | 0.93-2.88 | 0.089 |
| Pathologic T Stage |  |  |  |  |  |  |
| T1 | 1 |  |  |  |  |  |
| ≥T2 | 3.79 | 1.38-10.42 | **0.010** | 1.91 | 0.63-5.78 | 0.255 |
| Pathologic N Stage |  |  |  |  |  |  |
| N0 | 1 |  |  |  |  |  |
| N+ | 0.48 | 0.29-0.79 | **0.004** | 0.72 | 0.42-1.26 | 0.251 |
| Tumor location |  |  |  |  |  |  |
| Anastomoic site | 1 |  |  |  |  |  |
| Non-anastomoic site | 1.1 | 0.69-1.76 | 0.685 |  |  |  |
| Tumor Size | 1.02 | 1.01-1.03 | **<0.001** | 1.01 | 1.00-1.02 | 0.124 |
| LVI |  |  |  |  |  |  |
| No | 1 |  |  |  |  |  |
| Yes | 1.7 | 1.06-2.72 | **0.028** | 1.12 | 0.66-1.90 | 0.665 |
| Neoadjuvant chemotherapy |  |  |  |  |  |  |
| No | 1 |  |  |  |  |  |
| Yes | 1.59 | 0.85-2.97 | 0.144 |  |  |  |
| Adjuvant chemotherapy n (%) |  |  |  |  |  |  |
| No | 1 |  |  |  |  |  |
| Yes | 2.08 | 1.25-3.47 | **0.005** | 1.50 | 0.86-2.59 | 0.150 |

**eTable 11. Concurrent comparison of postoperative morbidity between LCTG and OCTG after IPTW**

|  | **OCTG (N=46)** | **LCTG (N=43.4)** | **P value** |
| --- | --- | --- | --- |
| **Overall morbidity, n (%)** | 17.4 (37.8%) | 12.2 (28.0%) | 0.318 |
| **Surgical morbidity** |  |  |  |
| Anastomotic leak, n (%) | 3.7 (8.0%) | 2.5 (5.7%) | 0.662 |
| Wound infection, n (%) | 5.8 (12.7%) | 1.4 (3.2%) | 0.097 |
| Ileus and gastroparesis, n (%) | 2.4 (5.2%) | 2.6 (5.9%) | 0.889 |
| Abdominal or anastomotic bleeding, n (%) | 3.3 (7.2%) | 3.7 (8.6%) | 0.804 |
| Abdominal abcess, n (%) | 3.1 (6.7%) | 1.0 (2.4%) | 0.330 |
| **Medical morbidity** |  |  |  |
| Urinary complications, n (%) | 0 (0%) | 0.7 (1.5%) | 0.398 |
| Respiratory complication, n (%) | 12.6 (27.3%) | 6 (13.9%) | 0.114 |
| Cerebrocardiovascular complication, n (%) | 0.8 (1.8%) | 0 (0%) | 0.362 |
| Clavien-Dindo Grade≥III, n (%) | 7.0 (14.9%) | 2.2 (5.0%) | 0.117 |

**eTable 12. Concurrent comparison of Surgical Outcomes and Recovery between LCTG and OCTG after IPTW**

| **Mean (SD)** | **OCTG (N=46)** | **LCTG (N=43.4)** | **P value** |
| --- | --- | --- | --- |
| **Surgical Outcome** |  |  |  |
| Blood loss, ml | 185.5 ± 125.8 | 59.7 ± 86.7 | **<0.001** |
| Time of operation, min | 179.2 ± 50.8 | 163.9 ± 37.1 | 0.104 |
| The number of harvest lymph nodes, n | 19.4 ± 11.8 | 19.2 ± 11.0 | 0.935 |
| Multiviceral resections, n (%) | 5.2 (11.3%) | 3.6 (8.3%) | 0.698 |
| **Recovery from surgery** |  |  |  |
| Removal of intra-abdominal drains, days | 12.0 ± 6.5 | 7.7 ± 3.0 | **<0.001** |
| Initiation of solid food intake, days | 8.9 ± 8.1 | 4.9 ± 2.9 | **0.002** |
| Time to Flatus, days | 4.4 ± 2.5 | 3.5 ± 2.1 | 0.051 |
| Discharge from the hospital (postoperative), days | 16.4 ± 8.4 | 11.9 ± 6.0 | **0.004** |

**eTable 13. Concurrent comparison of mean recurrence time between LCTG and OCTG before and after IPTW**

|  | **Time of recurrence, months** | | | | | |
| --- | --- | --- | --- | --- | --- | --- |
|  | **Before IPTW** | | | **After IPTW** | | |
|  | **OCTG (N=46)** | **LCTG (N=46)** | **P value** | **OCTG (N=46)** | **LCTG (N=43.4)** | **P value** |
| Overall | 8.4 ± 4.8 | 13.8 ± 8.7 | 0.164 | 8.1 ± 4.9 | 15.0 ± 9.1 | 0.054 |
| Local recurrence | 5.3 ± 0.6 | 6.0 ± 1.4 | 0.519 | 5.3 ± 0.6 | 5.7 ± 2.0 | 0.731 |
| Peritoneal recurrence | 6.8 ± 2.8 | 16.7 ± 10.2 | 0.170 | 6.8 ± 2.8 | 16.8 ± 9.9 | 0.095 |
| Distant metastasis | 9.0 ± 6.0 | 12.2 ± 7.9 | 0.372 | 8.5 ± 5.6 | 11.3 ± 7.7 | 0.379 |

**eTable 14. Per-protocol analysis of postoperative morbidity between LCTG and OCTG after IPTW**

|  | **OCTG (N=158.9)** | **LCTG (N=41.7)** | **P value** |
| --- | --- | --- | --- |
| **Overall morbidity, n (%)** | 55.6 (35.0%) | 10.4 (25.0%) | 0.300 |
| **Surgical morbidity** |  |  |  |
| Anastomotic leak, n (%) | 10.0 (6.3%) | 2.5 ( 6.0%) | 0.953 |
| Wound infection, n (%) | 17.3 (10.9%) | 1.4 ( 3.4%) | 0.126 |
| Ileus and gastroparesis, n (%) | 6.7 (4.2%) | 1.5 ( 3.6%) | 0.869 |
| Abdominal or anastomotic bleeding, n (%) | 5.1 (3.2%) | 3.1 ( 7.4%) | 0.362 |
| Abdominal abcess, n (%) | 7.5 (4.7%) | 0.0 ( 0.0%) | 0.150 |
| **Medical morbidity** |  |  |  |
| Urinary complications, n (%) | 1.9 (1.2%) | 0.7 ( 1.6%) | 0.817 |
| Respiratory complication, n (%) | 35.8 (22.5%) | 6.0 (14.5%) | 0.348 |
| Cerebrocardiovascular complication, n (%) | 1.7 (1.1%) | 0.0 ( 0.0%) | 0.472 |
| Clavien-Dindo Grade≥III, n (%) | 13.0 (8.2%) | 1.5 ( 3.6%) | 0.283 |

**eTable 15. Per-protocol analysis of Surgical Outcomes and Recovery between LCTG and OCTG after IPTW**

| **Mean (SD)** | **OCTG (N=158.9)** | **LCTG (N=41.7)** | **P value** |
| --- | --- | --- | --- |
| **Surgical Outcomes** |  |  |  |
| Blood loss, ml | 220.4 ± 197.7 | 60.1 ± 88.5 | **<0.001** |
| Time of operation, min | 225.7 ± 104.9 | 163.5 ± 37.2 | **<0.001** |
| The number of harvest lymph nodes, n | 15.6 ± 9.5 | 19.2 ± 11.2 | **0.033** |
| Multiviceral resections, n (%) | 26.3 (16.6%) | 3.6 (8.7%) | 0.200 |
| **Recovery from surgery** |  |  |  |
| Removal of intra-abdominal drains, days | 11.8 ± 11.0 | 7.3 ± 2.3 | **<0.001** |
| Initiation of solid food intake, days | 6.8 ± 7.3 | 4.6 ± 2.3 | **0.001** |
| Time to Flatus, days | 4.1 ± 2.5 | 3.2 ± 0.7 | **0.001** |
| Discharge from the hospital (postoperative), days | 18.7 ± 11.3 | 11.7 ± 5.9 | **<0.001** |
